# Fine Tuning of Tyrosine-Derived Amphiphilic and Bolaamphiphilic Gelators for the Formation of pH-Responsive Supramolecular Gels

**DOI:** 10.1021/prechem.5c00326

**Published:** 2026-01-21

**Authors:** Fabia Cenciarelli, Demetra Giuri, Silvia Pieraccini, Sofia Chinelli, Devis Montroni, Simone D’Agostino, Claudia Tomasini

**Affiliations:** Dipartimento di Chimica Giacomo Ciamician, 9296 Università di Bologna, Via Piero Gobetti, 85, Bologna 40129, Italy

**Keywords:** amphiphilic gelators, bolaamphiphilic gelators, circular dichroism, crystals, gels, rheology, tyrosine

## Abstract

Low-molecular-weight gelators (LMWGs) are small organic molecules that self-assemble through noncovalent interactions into fibrous networks capable of immobilizing solvents to create supramolecular gels that can be used in biomedical applications and other fields. Unlike robust polymeric gels, LMWGs offer tunability, stimuli-responsiveness, and easy degradation. Designing effective LMWGs requires a delicate balance of their weak, noncovalent interactions to form fibers instead of crystals. We investigated the critical molecular features required for the formation of a stimuli-responsive supramolecular gel derived from *O*-benzyl tyrosine. The amino moiety of the amino acid was coupled with six different fatty acids, yielding a series of compounds. Our screening revealed that Adi-[Tyr­(Bn)]_2_ and Aze-[Tyr­(Bn)]_2_ emerged as successful gelators, forming robust and responsive gels, while the other homologues formed less stable gels or precipitates, often yielding crystalline materials. The viscoelastic and self-assembly properties of the gels were comprehensively analyzed by rheology, electronic circular dichroism (ECD), and scanning electron microscopy (SEM), while single-crystal x-ray diffraction (SC-XRD) was used to confirm and elucidate their structural features. Interestingly, the ECD profile of Aze-[Tyr­(Bn)]_2_ was reminiscent of collagen-mimetic assemblies, which are of interest for applications in tissue engineering and the design of biomaterials.

## Graphical abstract

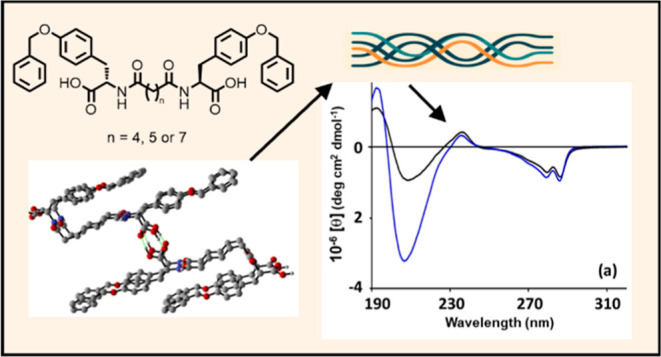

## 1 Introduction

Low-molecular-weight gelators (LMWGs) are small organic molecules capable of self-assembling into fibrous networks through noncovalent interactions such as hydrogen bonding, π–π stacking, and hydrophobic effects, giving rise to a versatile class of supramolecular materials.^1–3^ Upon triggering (by pH change or solvent variation), these molecules can immobilize large amounts of solvent, forming gels that behave as soft solids. Owing to their biocompatibility, modularity, and structural simplicity, supramolecular gels have found applications across diverse fields, ranging from biomedical and environmental technologies to catalysis and cosmetic formulations. Amphiphilic and bolaamphiphilic lipoamino acids belong to a well-established class of low-molecular-weight gelators in which self-assembly is governed by the balance between hydrophobic alkyl chains and amino-acid-based polar or charged units.^4–7^ Variations in molecular architecture, including chain length, symmetry, and headgroup composition, have been shown to strongly influence the aggregation pathways and gelation behavior. Closely related peptide- and lipopeptide-based amphiphiles, including bolaamphiphilic analogues, have been extensively studied as supramolecular gelators and provide important structure–property insights relevant to these systems. Several review articles summarize the design principles and gelation behavior of lipoamino-acid-based and bolaamphiphilic gelators.^8–10^

The comparison with traditional polymeric gelators^11–13^ present advantages and disadvantages. The main advantage of polymeric gelators is the robustness and the versatility of the gel formation that is strong and stable under several conditions, but these gels are slightly responsive to external stimuli and usually lack biodegradability.^14–17^ In contrast, the main advantage of supramolecular gelators is their tunability, their response to external stimuli, and their ability to easily degrade by cleavage of the weak interactions that are responsible for the fiber formation.^18–20^ Thus, the rational design of LMWGs capable of self-assembling into supramolecular architectures represents a crucial challenge in modern soft matter chemistry, as even minor molecular modifications can drastically alter their self-assembly and macroscopic behavior.^21–24^

For this reason, it is crucial to fine-tune each aspect of the process of gel formation, including solvent, temperature, time, and pH (if the solvent is water). Nevertheless, the gelator structure is the most important aspect that must be analyzed. Several authors demonstrated that specific properties are essential for an organic molecule to function as a gelator.^25–27^ Their capacity to form weak, noncovalent bonds—including π–π stacking, van der Waals interactions, hydrogen bonds, and electrostatic interactions—is necessary for the formation of the required fibrous structures. Crucially, however, the successful formation of fibers, rather than crystals or amorphous solids (which also rely on weak interactions), depends on a delicate and effective balance among these different noncovalent contributions.

With the aim of elucidating the optimal balance among these contributions, we synthesized a series of six lipoamino acids (Figure 1, Schemes S1 and S2). Each compound contained *O*-benzyl-l-tyrosine and a long-chain carboxylic acid derivative.^28^ The *O*-benzyl-l-tyrosine core was selected because it provides two key features: π–π stacking interactions via its two phenyl rings linked by a methylenoxy group and hydrogen bonds via its carboxyl group.

**Figure 1:**
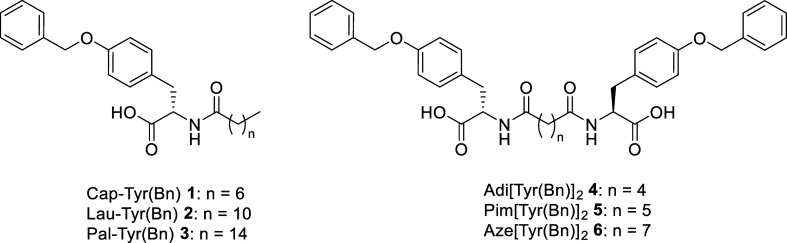
Chemical structures of the gelators **1–6** described in this work.

The amidation of this core with a long-chain carboxylic acid introduced both an amide functionality, capable of additional hydrogen bonding, and a hydrophobic alkyl chain, further contributing van der Waals interactions to the molecular self-assembly process.^29,30^ To systematically investigate the effect of chain length, we selected two groups of fatty acids:

- Monocarboxylic acids: caprylic acid (C_8_), lauric acid (C_12_), and palmitic acid (C_16_), which have increasing alkyl chain lengths for the formation of amphiphilic gelators;^31–34^

- Dicarboxylic acids: adipic acid (C_6_), pimelic acid (C_7_), and azelaic acid (C_9_), to broaden the scope of our structural analysis for the formation of bolaamphiphilic gelators.^35–37^

This work was inspired by the recent results that we obtained coupling l-Phe or l-Dopa­(Bn)_2_ with various fatty acids.^38,39^ In both cases, we discovered that the size of the aliphatic chain in our gelators critically determines their gelation ability, particularly when the pH is lowered. This pH change causes the acid group(s) to become protonated, a modification that can either trigger self-assembly into fibers or, in other cases, simply cause the molecule to precipitate. This highlights the delicate and often unpredictable balance between the molecular structure and self-assembly. It is a fine line that separates an effective gelator from a nongelling precipitate.

## 2 Results and Discussion

### 2.1 General Description

The molecules used in this study were prepared through a simple coupling reaction between the protected amino acid l-Tyr­(Bn)-OMe and the selected fatty acid. After purification by flash chromatography and by mild basic hydrolysis of the methyl ester (see Supporting Information for details), the final products **1–6** were obtained as white solids with final yields ranging around 80%, and they may all be prepared on a multigram scale. Compounds **1–6** were characterized by standard techniques, such as melting point, specific rotation, ^1^H and ^13^C NMR, FT-IR, and HPLC-MS (see Supporting Information for details).

To assess if these molecules may be ascribed to the family of gelators or to the family of nongelling precipitate, we tested their gelation ability in water. For the first attempt, we used all of the molecules at 1.0% w/v concentration.

The molecules were dissolved in water at pH ∼11, as under these conditions all molecules are water-soluble, even if they have long aliphatic chains. In some cases, the dissolution process required more time and was facilitated by the use of an ultrasound bath. Then, the solutions were subjected to pH variation until pH ∼4 by the addition of glucono-δ-lactone (GdL, a pH modifier).^40^ If the molecule is a gelator, as the pH is reduced, it self-assembles, forming fibers that, in turn, trap the solvent and form the supramolecular material. If the molecule is not a gelator, it simply precipitates (Figure 2).

**Figure 2:**
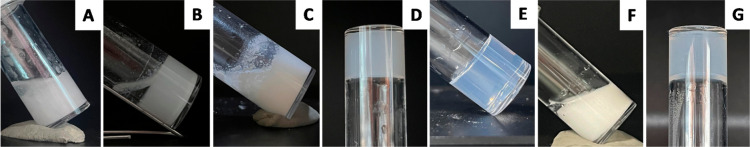
Photographs of the outcome after pH reduction from pH ∼11 to pH ∼4 of compounds **1–6**: (A) formation of precipitates from Cap-Tyr­(Bn) **1**; (B) formation of precipitates from Lau-Tyr­(Bn) **2**; (C) formation of precipitates from Pal-Tyr­(Bn) **3**; (D) formation of gel from Adi-[Tyr­(Bn)]_2_ **4**; (E) formation of gel from Pim-[Tyr­(Bn)]_2_ **5** after 3 h; (F) formation of precipitate from Pim-[Tyr­(Bn)]_2_ **5** after 18 h; and (G) formation of gel from Aze-[Tyr­(Bn)]_2_ **6**.

Despite sharing structural similarities, these molecules show distinctly different behaviors. Indeed, the amphiphilic tyrosine derivatives **1–3** form all nongelling precipitates at reduced pH, without including the solvent in the solid. In contrast, the bolaamphiphilic tyrosine derivatives **4–6** form gels, even if they exhibit a behavior that is strongly dependent on the chain length. While **4** and **6** form stable gels, **5** forms a gel after 3 h (Figure 2E) that quickly turns into a white solid that slowly precipitates (Figure 2F).

Globally, the behavior of these compounds is very different from what we obtained with amphiphilic derivatives of Phe^39^ and of Dopa­(Bn)_2_,^38^ even if, in principle, looking at their structures, their properties should be placed in between the other two sets of molecules that we previously studied. For example, both Pal-Phe and Pal-Dopa­(Bn)_2_ form gels, while Pal-Tyr­(Bn) certainly belongs to the family of nongelling precipitates. In particular, we demonstrated that Pal-Phe is a supergelator and is able to form gels at 0.025% w/v concentration.^41–43^

The rationalization of this aspect is quite challenging and may be ascribed to the subtle equilibrium between the soluble and insoluble products. For example, the addition of a benzyloxy group on the aromatic moieties, transforming Phe into Tyr­(Bn) reduces the water solubility, thus modifying their ability to self-assemble in aqueous solution. The addition of an additional benzyl group on the aromatic moieties, transforming Tyr­(Bn) into Dopa­(Bn)_2_ may further reduce the water solubility, strongly modifying the molecular behavior in a nonintuitive way. Odd–even effects in carbon-chain length have been reported to influence self-assembly and gelation behavior in certain low-molecular-weight gelators.^44–47^ However, in the present system, the available experimental data do not allow a clear correlation between the parity of the linker and the observed gelation profiles. In particular, the anomalous behavior of gelator **5** cannot be unambiguously ascribed to an odd–even effect based on the results obtained.

To understand how these compounds form gels, we first studied their properties in basic water. Their long hydrophobic chains make them insoluble in acidic solutions, where the carboxyl groups are protonated. However, at a pH of around 11, the carboxyl groups become deprotonated, which allows the molecules to dissolve in water. This dissolution process can take some time and may require an ultrasound bath because the compounds can self-assemble into structures like micelles or bilayers in water. We analyzed the aqueous basic solutions using several techniques prior to lowering the pH to an acidic value to investigate the self-assembly.

First, we determined the molecules’ apparent p*K*_a_ values by titrating a basic solution with hydrochloric acid (HCl).^38,48^ The p*K*_a_ of these compounds can change depending on their concentration and aggregation state, which is a common characteristic of amphiphilic and bolaamphiphilic molecules. This can lead to p*K*_a_ values that differ significantly from those of fully soluble acids such as acetic acid.

Figure 3 shows the results of titrating the six different molecules at a 0.5% w/v concentration. We started at pH 11 and added 0.1 M HCl until the pH reached 3. Each experiment was performed in triplicate to ensure accuracy and reproducibility. The p*K*_a_ was defined as the pH at which 50% of the molecules are protonated. In most cases, this value appeared as a plateau in the pH titration curve.

**Figure 3:**
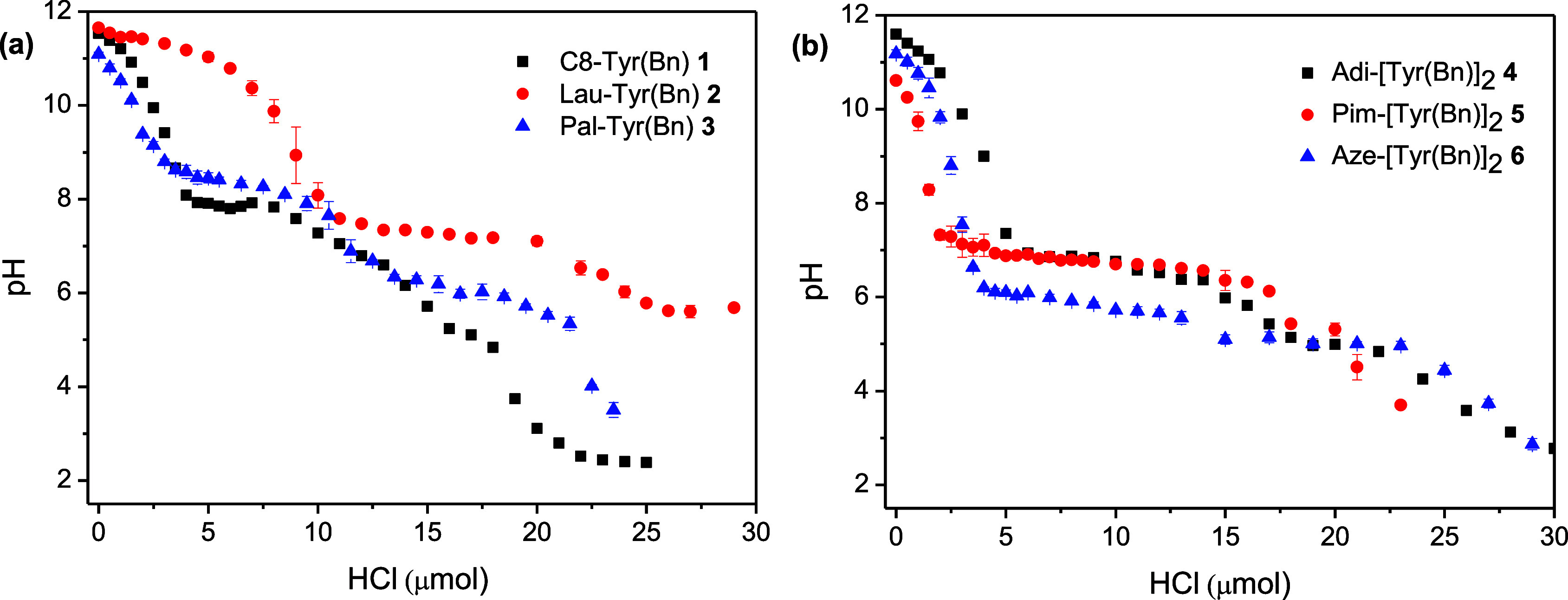
(a) Titration curves (pH versus added 0.1 M HCl) of compounds **1–3** and (b) titration curves (pH versus μmol of added HCl 0.1 M) of compounds **4–6**. The experiments were repeated in triplicate, and results are expressed as mean ± standard deviation.

A general insight into the titration plots suggests that all the molecules show an apparent p*K*_a_ well above the theoretical pH value of about 3.9, typical of small carboxylic acids that are not able to self-assemble.^49,50^ Among the amphiphilic molecules **1**, **2,** and **3**, the three molecules show roughly two plateaus that may be assigned at 8 and 6 for **1** and **3** and at 11 and 8 for **2**. In any case, well above 4. The behavior of **4**, **5,** and **6** is slightly different, as these three molecules show a single plateau ranging between 6 and 7, even if they all contain two carboxylic groups.

The self-assembly attitude in solution was further explored by ECD/UV spectroscopy, which is sensitive to both molecular and supramolecular chirality.^51–53^ For all six compounds, spectra were initially recorded in methanol, yielding clear samples. For the amphiphilic derivatives **1–3,** nearly superimposable curves were obtained (Figure S5a). Below 250 nm, absorption is dominated by the carboxyl and amide chromophores,^51^ although aromatic transitions may contribute.^54^ In this range, a positive ECD signal appeared at 228 nm. At the same time, in the 300–250 nm region, a weak negative–positive signal is observed, consistent with weak intramolecular exciton coupling between the aromatic chromophores.^51,53^ The phenyl rings are then supposed to adopt, on average, a slightly twisted arrangement in the methanol solution. For the bolaamphiphilic compounds **4–6**, similar band shapes were recorded in the near-UV range (Figure S5b, solid lines). The approximately 2-fold increase in intensity, compared to **1–3**, is ascribable to the doubling of the chromophoric units, contributing additively to the ECD signals. In the aromatic region (Figure S5b, dashed lines), **5** and **6** still display a negative exciton coupling, which is more intense compared to **1–3**. The ca. 10 nm red-shifting of the crossover may be indicative of a larger torsion angle and/or increased separation of the coupled aromatic moieties along the molecule.^51,53^ Conversely, derivative **4** exhibits an almost monosignal positive band. These spectral behaviors suggest that, for the bolaamphiphilic compounds, the linker length controls the overall molecular conformation in solution, which in turn affects ECD spectra. In particular, the longer linkers in **5** and **6** provide increased flexibility, allowing for stable exciton interactions between the terminal aromatic units. In contrast, the shorter linker in derivative **4** prevents such folding, and the observed optical activity primarily reflects the intrinsic chirality of the individual aromatic units. Dilution experiments were performed from 0.5 to 0.005% (w/v) (Figure S6). For most derivatives, only minor variations in ECD/UV band shapes and intensities were observed, compatible with the experimental error or instrumental noise. These results support that the observed signals primarily reflect molecular ECD, and aggregation effects are negligible in these conditions. However, for the bolaamphiphilic derivatives **4** and **6**, a marked hyperchromic effect in the UV absorption around 275 nm appears upon dilution, increasing in magnitude from **4** to **6** (see red traces in Figures S6d,f). This behavior may reflect disassembly of aggregates present at the higher concentrations.^52^ By moving to a basic aqueous medium, the nongelling amphiphilic compounds **1–3** gave highly opalescent systems, preventing the acquisition of reliable spectral data. By contrast, the spectra of derivatives **4–6** could be successfully recorded (Figures 4 and S7, red lines). Compared to the traces obtained in methanol (Figures 4 and S7, black lines), a slight hypochromic effect was observed for all UV absorption bands together with an increase in intensity of the ECD signals. Moreover, the positive band at 228 nm was blue-shifted to 224 nm. These variations can be attributed to self-association processes, possibly involving micelle formation. The intensity enhancement of the ECD aromatic bands increases noticeably from **6** to **5** to **4**, likely reflecting the self-aggregation propensity in an alkaline aqueous medium. This finding suggests that during the early stages of gelation at neutral or basic pH, the self-assembly of compound **6** proceeds more gradually, potentially giving rise to more regular supramolecular correlations. This aspect could account for the formation of a more highly ordered 3D network and a stronger hydrogel (see below).

**Figure 4:**
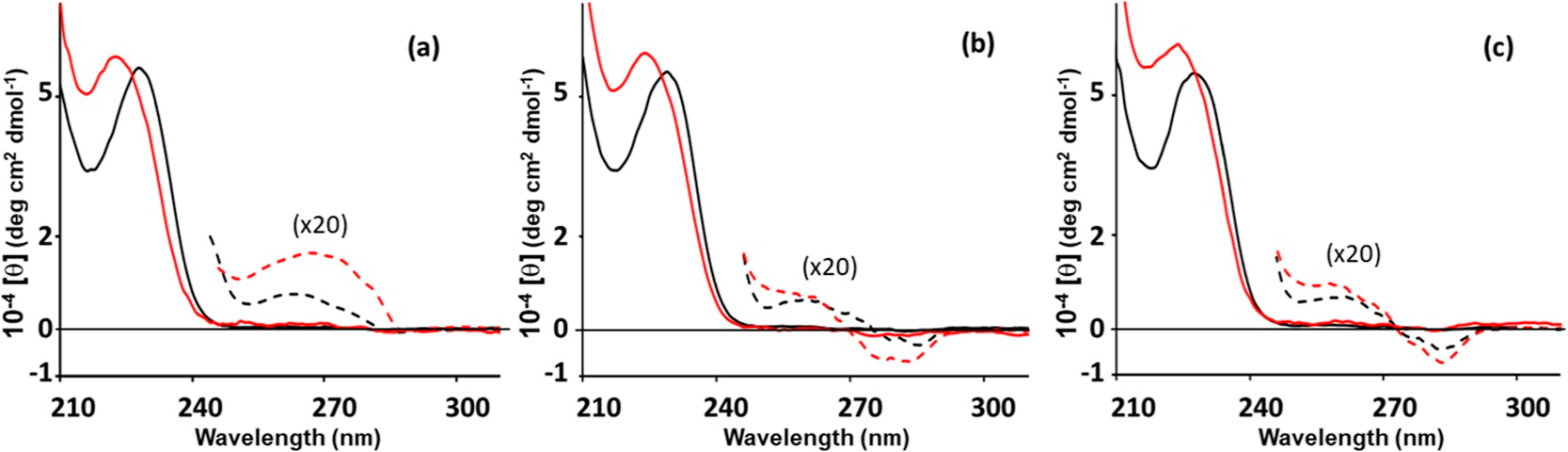
ECD spectra recorded on derivatives **4** (7.66 mM, (a)), **5** (7.49 mM, (b)), and **6** (7.19 mM, (c)) at 0.5% w/v in methanol (black traces) and alkaline water (red traces). Measurements were performed in a 0.01 cm cell (solid lines) and a 0.1 cm cell (dashed lines, multiplied by a factor of 20 for clarity).

### 2.2 Amphiphilic Derivatives of Tyr­(Bn)

The three compounds Cap-Tyr­(Bn) **1**, Lau-Tyr­(Bn) **2,** and Pal-Tyr­(Bn) **3** are not able to form hydrogels with the pH variation technique, as they simply self-aggregate, giving syneresis, without trapping the solvent. The solid may be crystalline or amorphous. The formation of crystals cannot always be reached for several reasons, including the correct molecular packing that often is not obtained.

The three compounds **1**, **2**, and **3** differ only in the chain length of the fatty acid, with each successive compound containing four additional carbon atoms. This small variation results in markedly different behaviors in crystal formation: compound **1** does not form crystals despite numerous attempts, whereas crystals were successfully obtained from compounds **2** and **3**. Compound **2** crystallizes upon the slow evaporation of a dichloromethane/ethyl acetate (DCM/AcOEt) solution, while compound **3** crystallizes upon the slow evaporation of a methanol solution.

Compound Lau-Tyr **2** crystallizes in the monoclinic system with the P2_1_ space group (see Table S2 for crystallographic details), containing one molecule in the asymmetric unit (Figure 5A). As shown in Figure 6A, within the crystal lattice, molecules of Lau-Tyr **2** engage in multiple hetero-intermolecular hydrogen-bonding interactions involving N–H···O and O–H···O contacts between the amide and carboxylic functions, respectively, giving rise to an extended three-dimensional network [N_N–H_···O_COOH_ = 2.87(2) Å and O_COOH_···O_NCO_ = 2.87(2) Å].

**Figure 5:**
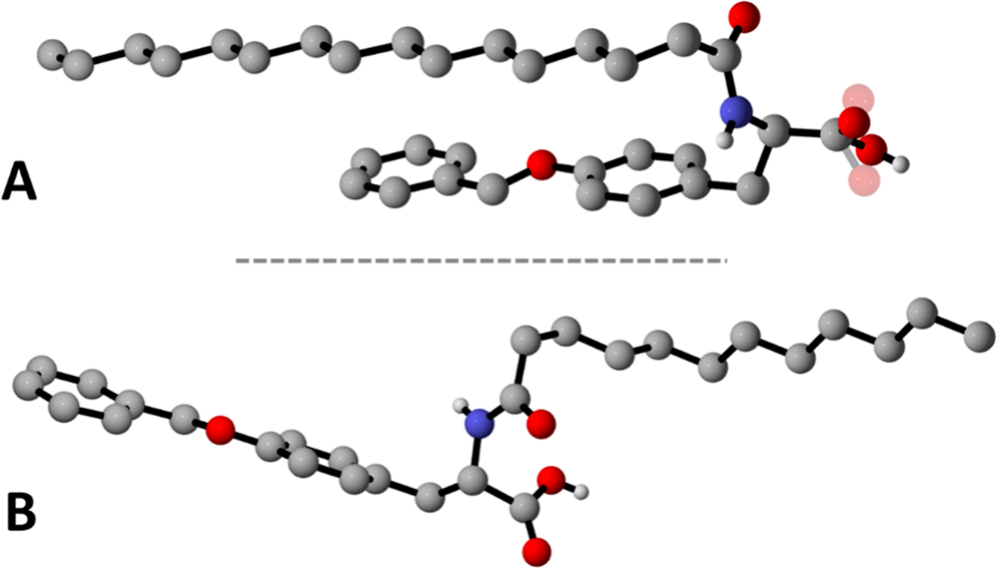
Molecular structure of Lau-Tyr­(Bn) **2** (A) and Pal-Tyr­(Bn) **3** (B) as determined from single SC-XRD data. Disorder affecting the COOH groups of **2** is depicted in transparency. H_CH_ is omitted for clarity.

**Figure 6:**
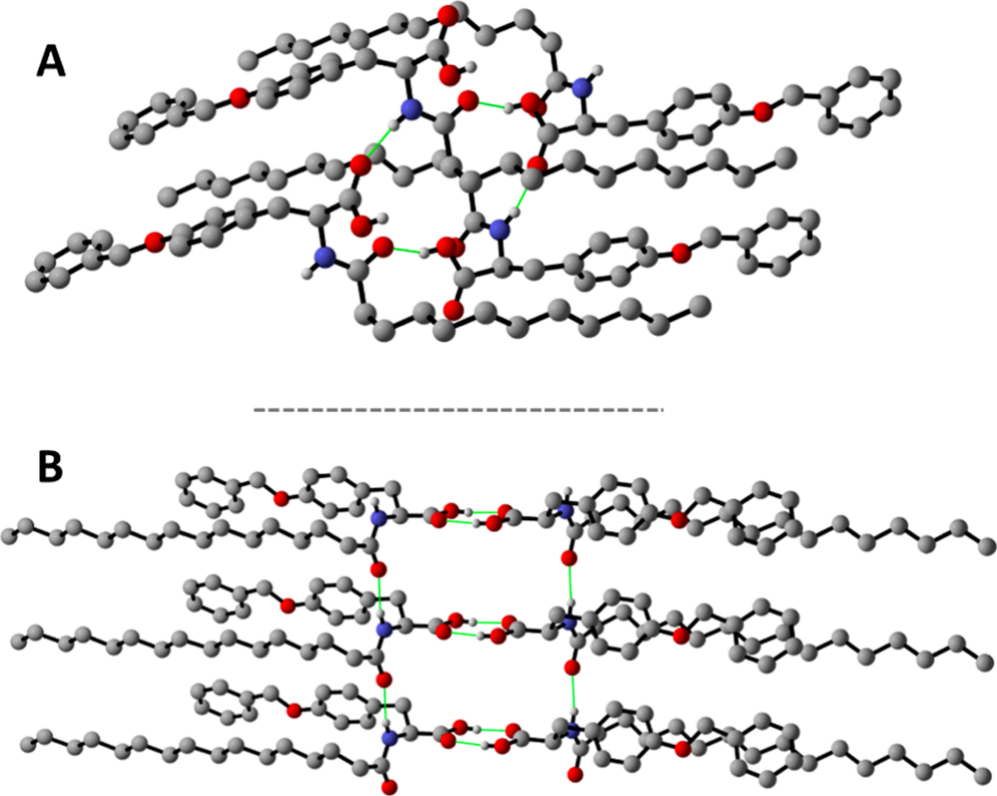
Intermolecular hydrogen bonding interactions detected within crystalline: (A) Lau-Tyr­(Bn) **2** and (B) Pal-Tyr­(Bn) **3**. Hydrogen bonds are depicted in green, disorder in **3** not shown, and H_CH_ is omitted for clarity.

Compound Pal-Tyr **3** crystallizes in the monoclinic system with the C2 space group (see Table S2 for details), also with one molecule in the asymmetric unit (Figure 5B). In this case, structural disorder affecting the carboxylic groups was also observed. Unlike compound Lau-Tyr **2**, which forms an extensive 3D hydrogen-bonded network held together by hetero-intermolecular interactions, crystalline Pal-Tyr **3** exhibits a less cross-linked supramolecular architecture leading to the formation of 1D tapes as the result of homo-intermolecular hydrogen-bonding interactions between pairs of carboxylic and amide functions (Figure 6B). Specifically, carboxylic acid groups engage in the typical cyclic dimeric motif [O_COOH_···O_COOH_ = 3.16(5)–2.84(5) Å], while additional hydrogen bonds between amide functionalities further stabilize the overall packing arrangement [N_N–H_···O_C=O_ = 2.880(7) Å].

### 2.3 Bolaamphiphilic Derivatives of Tyr­(Bn)

In contrast to other derivatives, the bolaamphiphilic compounds Adi-[Tyr­(Bn)]_2_ **4**, Pim-[Tyr­(Bn)]_2_ **5**, and Aze-[Tyr­(Bn)]_2_ **6** act as gelators, forming hydrogels under acidic conditions (as shown in Figure 2). To gain deeper insight into their self-assembly mechanism, we analyzed and compared their critical aggregation concentration (CAC) (Figure S2),^55^ their propensity to form nanostructures using dynamic light scattering (DLS) (Figures S3 and S4),^56,57^ and their minimum gelation concentration (MGC) (Table S1 and Figure S1). These findings are summarized in Table 1, with full details provided in the Supporting Information.

**Table 1:** Results Obtained for the Study of the Self-Assembly of Bolaamphiphilic Derivatives Adi-[Tyr­(Bn)]_2_ **4**, Pim-[Tyr­(Bn)]_2_ **5** and Aze-[Tyr­(Bn)]_2_ **6**

	pH ∼11				pH ∼4	
gelator	critical aggregation concentration		dynamic light scattering		minimum gelation concentration	
Adi-[Tyr(Bn)]_2_ **4**	1.5 × 10^–2^ mM	1.0 × 10^–3^% w/v	6 × 10^–2^ mM	3.8 × 10^–3^% w/v	9.5 mM	0.6% w/v
Pim-[Tyr(Bn)]_2_ **5**	2.0 × 10^–2^ mM	1.3 × 10^–3^% w/v	10 × 10^–2^ mM	6.7 × 10^–3^% w/v	14.9 mM	(1.0% w/v) metastable
Aze-[Tyr(Bn)]_2_ **6**	1.5 × 10^–2^ mM	1.0 × 10^–3^% w/v	20 × 10^–2^ mM	14 × 10^–3^% w/v	1.4 mM	0.1% w/v

The hydrogel formation process begins with molecular aggregation. The CAC analysis indicates similar initial aggregation behavior for all three molecules, with aggregation detected at approximately 10^–2^ mM.

Nanostructure formation, measured by DLS, occurs at a concentration roughly 10-fold higher than the CAC. Nanostructures with an average diameter of approximately 100 nm were detected, with the required concentration ranging from 6 × 10^–2^ mM for Adi-[Tyr­(Bn)]_2_ **4** to 20 × 10^–2^ mM for Aze-[Tyr­(Bn)]_2_ **6**. These data indicate that the tendency to self-assemble increases slightly in the order **4** > **5** > **6**, a trend supported by ECD results obtained under alkaline aqueous conditions (see above). No nanostructure formation was detected at concentrations below this range.

As expected, the subsequent formation of fibers via nanostructure self-aggregation is detected at higher concentrations after the pH is lowered.

Crucially, Aze-[Tyr­(Bn)]_2_ **6**, the derivative with the longest chain, proved to be the most effective gelator, forming a transparent, self-supporting gel at the lowest MGC of 1.4 mM (0.1% w/v). The derivative with the shortest chain, Adi-[Tyr­(Bn)]_2_ **4**, required a much higher MGC (9.5 mM, 0.6% w/v). The intermediate derivative, Pim-[Tyr­(Bn)]_2_ **5**, was the poorest gelator; its MGC was 14.9 mM (1.0% w/v), and the resulting gel lacked stability, turning opaque and releasing liquid within approximately 16 h (Figure 2).

These data suggest that the chain length is a critical factor in this system. The added flexibility afforded by the longer chain appears to facilitate better self-aggregation under acidic conditions, an effect that was not observed under basic conditions, where the CAC and DLS results for the three compounds were comparable, although compound **4** is the most prone to form nanostructures, as detected with DLS.

To have a deeper insight into the self-aggregation of Adi-[Tyr­(Bn)]_2_ **4**, Pim-[Tyr­(Bn)]_2_ **5** and Aze-[Tyr­(Bn)]_2_ **6** under acidic conditions, we analyzed the gels using rheological techniques. These techniques allow us to measure the mechanical properties of a material in terms of the storage modulus (*G*′) and of the loss modulus (*G*″).^58–61^ When the storage modulus is higher than the loss modulus, the sample is a solid (including a gel); in the opposite situation, the sample is a liquid. The higher the value of the storage modulus, the stronger the gel. First, we analyzed the gel formation by measuring the variation of the *G*′ and of the *G*″ from the beginning of the gel formation for about 16 h, that is, the normal time after which we consider the gel formation concluded. To better compare their rheological properties, all the samples were prepared in 1.0% w/v concentration.

The rheological time sweep data, shown in Figure 7, clearly illustrate the distinct aggregation mechanisms initiated by the three compounds. Compound **4** exhibits rapid gelation, with the storage and loss moduli immediately increasing upon mixing. The gel continuously strengthens over the 16 h period, ultimately achieving a medium stiffness (*G*′ ≈10^3^ Pa) (Figure 7a). In contrast, the time sweep for compound **5** (Figure 7b) reveals a more complex evolution. An initial stiffness maximum is reached after approximately 3 h, yielding a gel strength similar to that of **4**. Critically, the minimal separation between the storage modulus (*G*′) and the loss modulus (*G*″) at this peak suggests a viscoelastic state very close to the liquid–solid transition boundary, unlike the highly elastic nature of gel **4**. This initial peak is attributed to the formation of a weak metastable gel network. Following this, both moduli decrease after 5–6 h but subsequently show a sharp increase around 10 h, where *G*′ becomes significantly larger than *G*″, confirming the formation of a solid phase, likely crystalline in nature. The continuous decline of both parameters after this peak is ascribed to the precipitation of this crystalline material, which effectively reduces the solute concentration in the remaining solution. This rheological profile perfectly aligns with macroscopic observations of the transparent gel transforming into a white solid over the course of a few hours. The time sweep analysis for compound **6** (Figure 7c) shows yet another mechanism. Gel formation is accompanied by an initial, continuous increase in both *G*′ and *G*″, which reaches a peak after roughly 6 h. Following a minor drop, both moduli increase again, concluding the 16 h sweep at the same final *G*′ and *G*″ values. This biphasic increase suggests a potential gel-to-gel transition occurring over time, resulting in the formation of a highly stable hydrogel.

**Figure 7:**
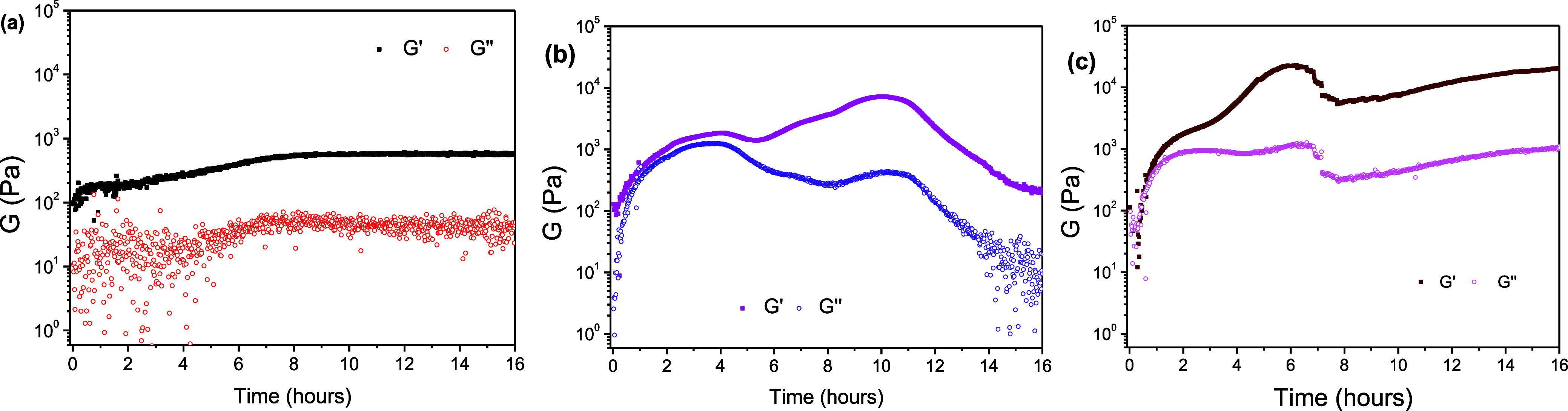
Time sweep analysis of 1.0% w/v samples: (a) Adi-[Tyr]_2_ **4**, (b) Pim-[Tyr]_2_ **5,** and (c) Aze-[Tyr]_2_ **6**.

The viscoelastic properties of the stable hydrogels formed by compounds **4** and **6** (at 1% w/v concentration) were confirmed by recording the amplitude sweep profiles after 16 h of preparation (Figure 8a,c). Both samples demonstrate excellent viscoelastic behavior, characterized by a linear viscoelastic region (LVR) that extends to high strain values before the crossover point, where the storage modulus (*G*′) becomes lower than the loss modulus (*G*″). For compound **4** (Figure 8a), the crossover point is close to a strain (γ) of 100%. For compound **6** (Figure 8c), the crossover occurs at approximately γ = 75%. A comparison of the moduli at a low strain (γ = 1%) reveals the relative stiffness:

- Compound **4** gel: *G*′ = 1.87 kPa and *G*″ = 0.23 kPa.
- Compound **6** gel: *G*′ = 28.34 kPa and *G*″ = 6.28 kPa.

**Figure 8:**
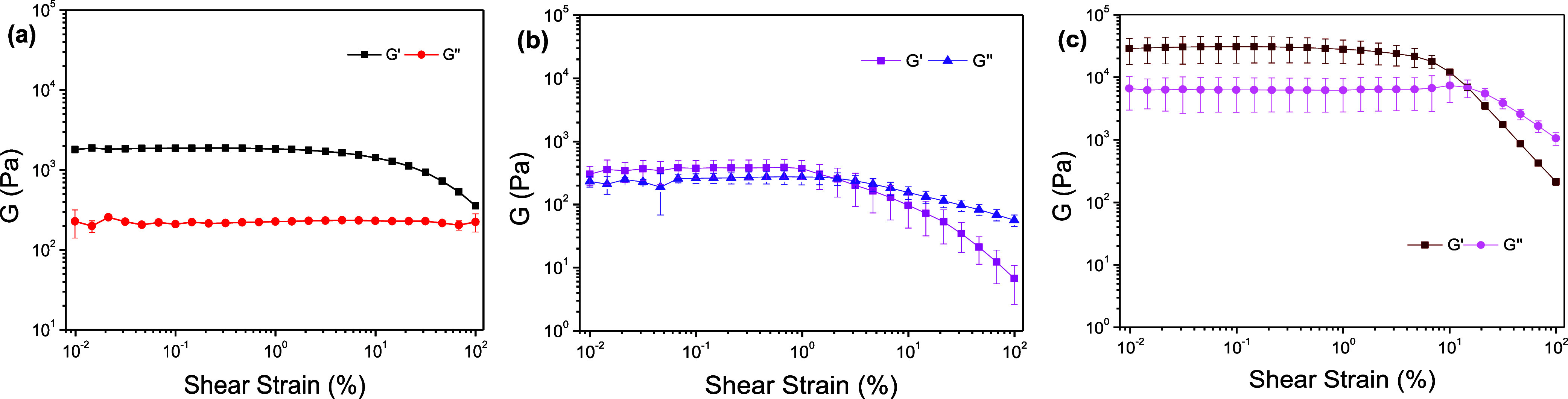
Amplitude sweep test of the hydrogels at 1.0% w/v. From left to right: (a) Adi-[Tyr]_2_ **4**; (b) Pim-[Tyr]_2_ **5**, recorded 3 h after the addition of GdL; and (c) Aze-[Tyr]_2_ **6**. The experiments were repeated in triplicate, and results are expressed as mean ± standard deviation.

The viscoelastic properties of the metastable gel obtained from compound **5** were analyzed after 3 h (before the gel-to-crystal transition) (Figure 8b). The amplitude sweep confirms the poor structural integrity previously observed in the time sweep (Figure 7b). At low strain (γ = 1%), the *G*′ and *G*″ values are very low and very close (*G*′ = 0.39 and *G*′′ = 0.27 KPa). Furthermore, the rapid loss of solid-like behavior is indicated by the crossover point occurring at a very low strain of γ = 2.6%. This limited LVR confirms the poor mechanical properties of this gel, placing it at the borderline between a viscoelastic solid and a viscous liquid.

IR spectral analysis of the hydrogels and aerogels (data not shown) did not reveal any significant shifts in the stretching bands that could aid in elucidating the self-assembly mechanism. Gelation of **6** was further investigated by ^1^H NMR spectroscopy in an NMR tube. Immediately after the addition of GdL (*t* = 0), the ^1^H NMR spectrum closely resembled that of the basic solution. In contrast, after 18 h, the signals associated with the gelator were no longer detectable, consistent with fiber formation (Figure S8).

The supramolecular organization of the three gel phases was investigated by an ECD analysis. Spectra recorded on hydrogels **4** (black trace) and **6** (blue trace) after 18 h are shown in Figure 9a. Based on the relative intensities and positions of the positive and negative peaks below 250 nm, the ECD curves resemble those reported for helical arrangements of collagen-inspired short-peptide hydrogels.^62–64^ Natural collagen displays a typical ECD signature with a small positive peak at ca. 225 nm and a large minimum near 200 nm, indicative of a polyproline type II helix.^62^ In the present case, a positive signal occurs at ca. 235 nm, while the negative band is centered around 208 nm. While these features do not allow an unambiguous structural assignment, they are noteworthy in comparison with related systems. Indeed, analogous aromatic derivatives studied previously exhibited negative minima at 218–220 nm, commonly associated with β-sheet structures.^38,39^ Moreover, a benzylated dityrosine dipeptide, reminiscent of our bolaamphiphilic gelators, has been shown to form β-sheet arrangements either in single crystals or in solution, as evidenced by a broad negative ECD band at 218 nm.^65^ In our hydrogels, however, the canonical β-sheet minimum is absent, thus pointing to a different supramolecular organization.

**Figure 9:**
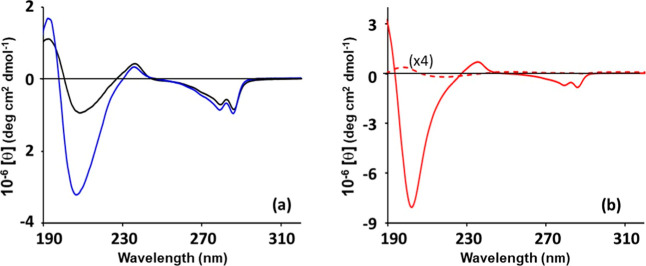
ECD spectra recorded on hydrogels of (a) derivatives **4** (0.6% w/v, black trace) and **6** (0.5% w/v, blue trace) after 18 h and (b) derivative **5** (1% w/v) after 3 h (red solid trace) and 18 h (red dashed trace, multiplied by a factor of 4). A 0.001 cm cell was used.

Compared to sample **6**, sample **4** exhibits a weaker negative band, suggestive of a lower degree of supramolecular order and stability, in agreement with the rheological data. In the near-UV region, between 300 and 250 nm, both systems display a pronounced negative band with vibronic structure, arising from the aromatic residues embedded in the chiral environment. As shown in Figure 9b, the spectrum of the **5** hydrogel after three hours (red solid line) displays spectral features comparable with **6** and **4**. However, in the far-UV region, the intense negative signal appears narrower and less symmetric and is shifted to 203 nm. In this case, after 18 h, only minimal optical activity is detected (red dashed line), indicating that the original arrangement is lost.

To complete the analysis of the metastable gelator Pim-[Tyr­(Bn)]_2_ **5**, single crystals were grown from methanol and analyzed by single-crystal x-ray diffraction to elucidate its structural features.

Compound Pim-[Tyr­(Bn)]_2_ **5** crystallizes in the monoclinic system with the *C*_2_ space group (see Table S2 for crystallographic details) and contains one molecule in the asymmetric unit (Figure 10). For this compound, the structure exhibits disorder involving both the carboxylic functions and the benzylic group. Within crystalline Pim-[Tyr­(Bn)]_2_ **5**, each molecule establishes homo-intermolecular hydrogen-bonding interactions between pairs of carboxylic functions and amide groups, giving rise overall to an extended three-dimensional supramolecular network. Specifically, the amide functions form hydrogen bonds [N_N–H_···O_C=O_ = 2.922(6) Å], while the carboxylic groups establish the characteristic cyclic dimers, marked as **I** and **II** in Figure 11 [O_COOH_···O_COOH_ (**I**) = 2.621(8) – 2.684(8) Å and O_COOH_···O_COOH_ (**II**) = 2.604(1) – 2.802(1) Å].

**Figure 10:**
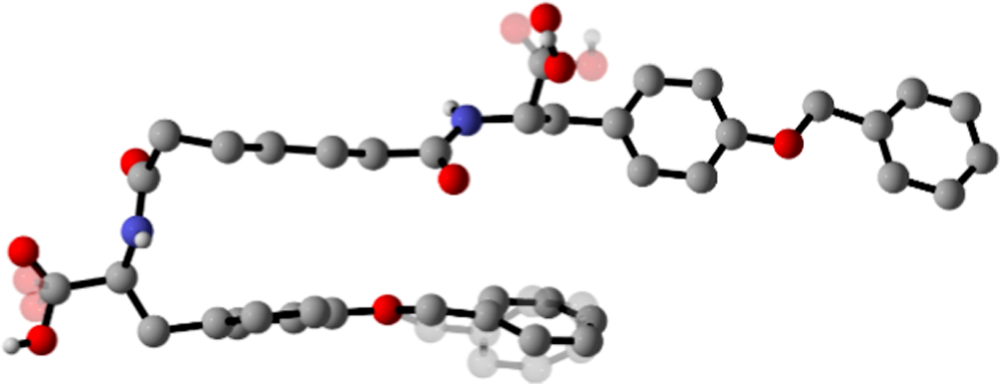
Molecular structure of Pim-[Tyr­(Bn)]_2_ **5,** as determined from SC-XRD data. Disorder affecting the carboxylic functions and benzyl group depicted with transparency and H_CH_ omitted for clarity.

**Figure 11:**
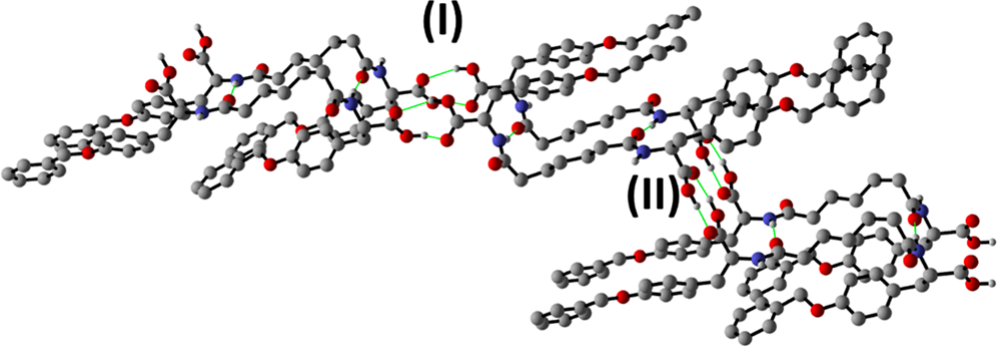
Intermolecular hydrogen bonding interactions detected within crystalline Pim-[Tyr­(Bn)]_2_ **5**. Hydrogen bonds are depicted in green, disorder is not shown, and H_CH_ is omitted for clarity. The two different cyclic dimers formed between the COOH are marked as **I** and **II**.

To gain structural insights into the supramolecular organization of the bolaamphiphilic gelators, aerogels obtained from Adi-[Tyr­(Bn)]_2_ **4**, Pim-[Tyr]_2_ **5**, and Aze-[Tyr]_2_ **6** were examined by scanning electron microscopy (SEM). Representative images are shown in Figure 12. As the metastable gel of Pim-[Tyr­(Bn)]_2_ **5** converts into two distinct macroscopic morphologies over time, the gel was collected and analyzed after 3 and 18 h from GdL addition.

**Figure 12:**
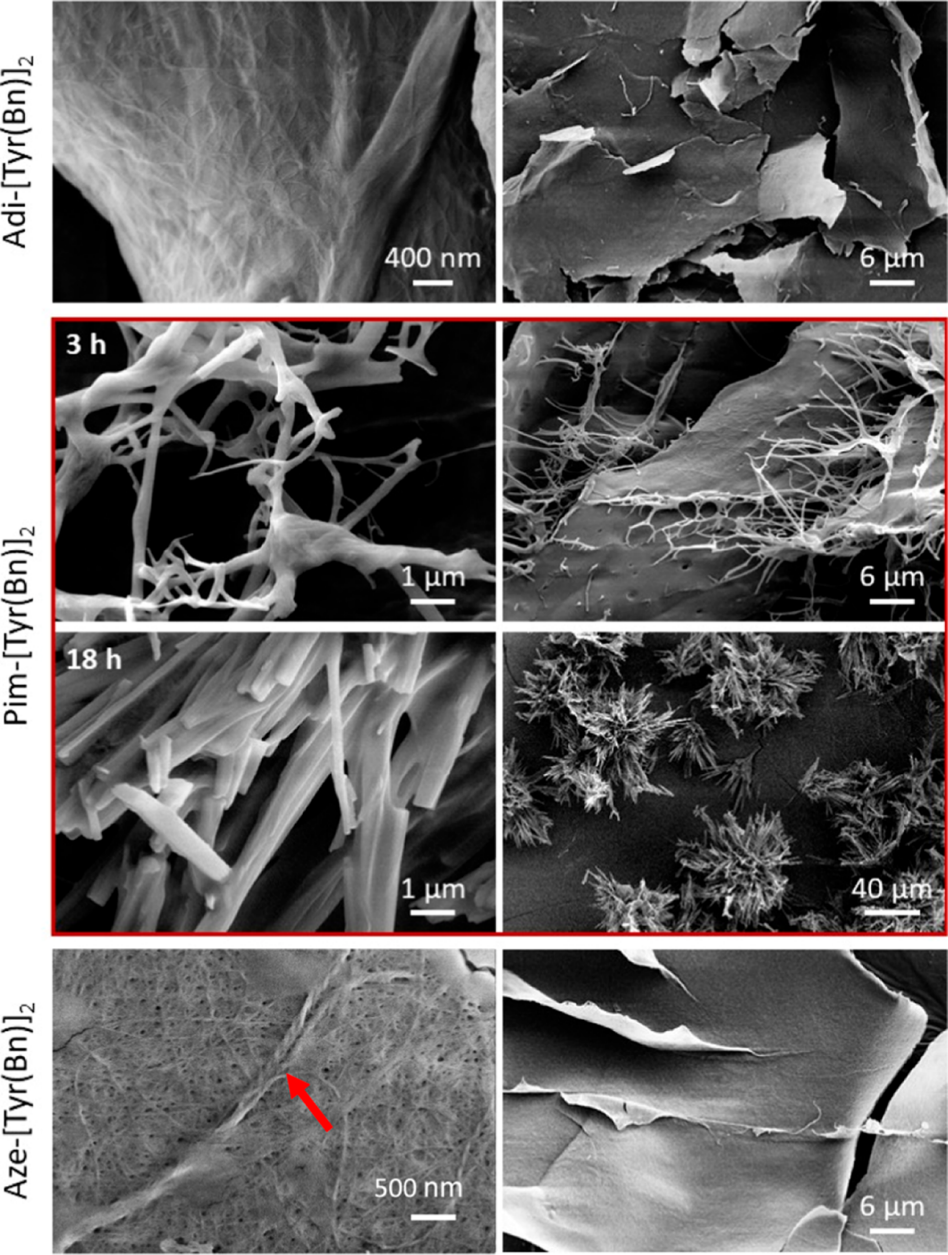
SEM images of freeze-dried gels obtained from bolaamphiphilic derivatives: (top) Adi-[Tyr­(Bn)]_2_ **4** (0.6% w/v); (middle) Pim-[Tyr­(Bn)]_2_ **5** (1.0% w/v) after 3 and 18 h from the trigger; (bottom) Aze-[Tyr­(Bn)]_2_ **6** (0.5% w/v). For each sample, an image at high (left) and low (right) magnification is reported. Magnification has been adjusted depending on the size of the features.

At high magnification, the gelators **4**, **5** (3 h), and **6** exhibit a nanofibrous morphology, while at low magnification, a thin sheet morphology is generally observed. A coiled-coil structure is clearly visible in the magnified image of compound **6** (red arrow).

Adi-[Tyr­(Bn)]_2_ **4** showed a compact nanofibrillar morphology with fibrils in the 4–45 nm range of thickness, reflecting a homogeneous but scarcely crystalline aggregation mode. Such a morphology correlates with its rheological profile, which revealed a soft yet persistent gel with moderate stiffness and strong viscoelastic character.

At 3 h, Pim-[Tyr­(Bn)]_2_ **5** shows a mixed morphology of compact nanofibril blankets and poorly interconnected thick fibrils in the 80–400 nm range of diameter, forming an open three-dimensional structure. Contrary to gelator **4**, the thin layers formed are much larger. This morphology is typical of soft, weakly interconnected supramolecular gels, which is in agreement with the rheological data.

At 18 h, gelator **5** changes the morphology drastically. The irregular but smooth fibrils observed at 3 h disappeared, leaving only wide and stiff ribbon-like structures with a crystalline appearance. These crystalline structures appear aggregated in spherulites of about 100 μm with no interconnections between one another, thus losing complete interconnectivity. Such a morphology cannot be reached by a mere structural rearrangement and requires a dissolution-reprecipitation process on a common nucleating center. This morphological transition confirms the gel-to-crystal conversion suggested by time-sweep rheology, highlighting the metastable nature of this system.

Finally, Aze-[Tyr­(Bn)]_2_ **6** shows an entangled fibrillar morphology with fibrils in the 4–14 nm range forming sheets of intermediate size compared to the other two gelators. Although these fibrils are the thinnest among the samples, the porous texture observed suggests that they extend over lengths longer than those produced by the previous two gelators. This morphology prevents tight fibrillar packing and results in a less compact fibrillar network but enables much higher interconnectivity in the gel. The longer and thinner fibrils observed are consistent with the formation of a robust and stable gel, as is visible by its high storage modulus. Notably, the magnified image of **6** reveals the presence of a coiled-coil structure, indicated by a red arrow. This observation is consistent with our previous ECD analysis, which indicated the formation of helical assemblies of collagen-inspired helices.

Overall, the SEM analysis confirms the correlation between the microstructure and macroscopic gel stability: while Adi-[Tyr­(Bn)]_2_ **4** and Aze-[Tyr­(Bn)]_2_ **6** form stable fibrillar networks typical of tough hydrogels, Pim-[Tyr­(Bn)]_2_ **5** evolves from a soft fibrous gel into a crystalline material, illustrating the delicate balance between supramolecular order, molecular flexibility, and long-term stability.

## 3 Conclusions

In this study, we successfully synthesized and characterized six novel lipoamino acids, establishing the bolaamphiphilic structure as the critical requirement for Tyr­(Bn)-based hydrogelation via pH change. In contrast, the simpler amphiphilic derivatives failed to gel, forming precipitates or crystalline materials.

The core requirement for a hydrogel is the formation of a stable, interconnected, three-dimensional network of fibers that traps the solvent. When the pH drops, the molecules protonate (COO^–^ → COOH), lose their water solubility, and spontaneously assemble.

The different behaviors of amphiphilic and bolaamphiphilic compounds may be explained by considering their structure.

Amphiphilic derivatives have a single Tyr head and a single long aliphatic tail that, likely, favor the irreversible aggregation into dense, nongelling precipitates or crystalline materials, without leaving enough voids or solvent–accessible interfaces to create a porous gel, i.e., they are too efficient at maximizing their own density.

In contrast, bolaamphiphilic derivatives have an aliphatic linker connecting two Tyr heads. With two Tyr units per molecule, they have twice the opportunity for π–π stacking and two COOH groups that can dimerize, as seen in the crystal structure of **5**. This doubling of stabilizing interactions allows the molecules to engage in a more effective fiber-to-fiber correlation and cross-linking. The aliphatic linker provides a spacer, preventing the molecules from collapsing into a dense precipitate immediately.

Crucially, within the successful bolaamphiphilic family **4–6**, the length and flexibility of the aliphatic linker dictated gelator efficacy and stability. The longest-chain derivative, Aze-[Tyr­(Bn)]_2_ **6**, demonstrated superior self-assembly in acidic conditions, yielding the most interconnected and mechanically robust hydrogel at the lowest concentration. This superior performance over gelators **4** and **5** suggests that the increased molecular flexibility of the azelaic linker in **6** permits better long-range structural correlation and stability during the final fiber-to-network assembly. The intermediate-chain derivative, Pim-[Tyr­(Bn)]_2_ **5**, serves as an important boundary condition, forming a highly delicate, metastable gel near the nongelling/crystalline threshold.

Interestingly, the ECD profiles of **4–6** suggest collagen-inspired architectures, a possibility that warrants further investigation due to its potential relevance for biomaterials. Given their robust and stable nature, the bolaamphiphilic derivatives, particularly **6**, open the door to exciting applications as controllable drug delivery systems, injectable scaffolds for tissue engineering, wound healing, hemostasis, and smart materials/sensors.

## 4 Experimental Section

### 4.1 General Remarks for the Synthetic Procedure

All reactions were carried out in dried glassware. The melting points of the compounds were determined in open capillaries and are uncorrected. All compounds were dried in vacuo, and all the sample preparations were performed in a nitrogen atmosphere. High-quality infrared spectra (64 scans) were obtained at 2 cm^–1^ resolution with an ATR-IR Agilent (Santa Clara, CA, USA) Cary 630 FTIR spectrometer. NMR spectra were recorded with a Varian (Palo Alto, CA, USA) Inova 400 spectrometer at 400 MHz (^1^H NMR) and 100 MHz (^13^C NMR). Chemical shifts are reported in δ values relative to the solvent peak. An Agilent (Santa Clara, CA, USA) 1260 Infinity II liquid chromatograph coupled to a Mass Spectrometer MSD/XT equipped with an electrospray ionization source and operating with a single quadrupole mass analyzer was used to check the purity of compounds. HPLC was equipped with a Phenomenex Gemini C18—3 μ—110 Å column (40 °C), and H_2_O/CH_3_CN with 0.2% formic acid was used as the solvent. The MS was used in positive ion mode, *m*/*z* = 50–2000, fragmentor 70 V. Milli-Q water (Millipore, resistivity = 18.2 mΩ cm) was used throughout. A Jasco (Mary’s Court, MD, USA) P-2000 Polarimeter was used to check the optical rotatory power of the compounds. Boc-l-Tyr­(Bn)-OH was purchased from Novabiochem. All the solvents were purchased from Sigma-Aldrich (St. Louis, MO, USA).

### 4.2 Methodology for Gel Preparation

The gels used for the rheological analysis and the MGC studies were prepared in 7.0 mL Sterilin Cups. The gels used for the IR spectra were directly prepared in 2 mL of HPLC glass vials. In both cases, we used the following procedure: the gelator was suspended in Milli-Q H_2_O, and 1 M aqueous NaOH was added (1.2 equiv for gelators **1**, **2,** and **3**; 2.2 equiv for gelators **4**, **5,** and **6**). The solution was stirred and sonicated until complete dissolution of the gelator. To trigger the formation of the gel, solid glucono-δ-lactone (GdL) (1.3 equiv for gelators **1**, **2,** and **3**; 2.3 equiv for gelators **4**, **5,** and **6**) was added, and the mixture was immediately gently swirled to allow the dissolution of the trigger. After rapid mixing and complete dissolution of GdL, the sample was allowed to stand quiescently until gel formation (overnight).

### 4.3 Methodology for the Determination of the Apparent p*K*_a_

A XS pH70 Vio Portable pHmeter (XS Instruments, Carpi (MO), Italy) with a 2-pore steel T electrode was employed for all pH measurements. The stated accuracy of the pH measurements is ±0.1. The pH meter was calibrated before each experiment to check the response of the electrode. Depending on the desired concentration, considering a total volume of 2 mL for each sample, the required amount of gelator was suspended into Milli-Q H_2_O. Sodium hydroxide (1 M aqueous solution) was added to the aqueous suspensions of gelator until the pH ≈11 was reached. The samples were vortexed and sonicated to fully dissolve the gelator. The p*K*_a_ values of gelator solutions were determined by titration via the addition of aliquots of a 0.1 M aqueous HCl solution in triplicate. During the titration process, the pH values were recorded until a stable value was reached after each addition. Due to the strong and constant agitation applied, samples were liquid at all times during the titration experiments.

### 4.4 Minimum Gelation Concentration Studies

The samples were prepared by adding Milli-Q water and aqueous 1 M NaOH (1.2 equiv. for gelators **1**, **2,** and **3**; 2.2 equiv. for gelators **4**, **5,** and **6**) to a 7 mL Sterilin Cup containing a known quantity of the compound in order to produce a certain final concentration of the gelator. The mixture was stirred and sonicated for about 1 h until the complete dissolution of the sample. The pH of this solution was measured to be between 10.6 and 11.6. Then, glucono-δ-lactone (GdL) (1.3 equiv. for gelators **1**, **2,** and **3**; 2.3 equiv for gelators **4**, **5,** and **6**) was added to the mixture. After a rapid mixing and complete dissolution of GdL, the sample was allowed to stand quiescently until gel formation (overnight). After 16 h, the samples were examined by a vial inversion test.

### 4.5 Critical Aggregation Concentration

To evaluate the aggregation propensity of the three bolaamphiphilic gelators **4–6** under basic conditions, the critical aggregation concentration (CAC) was determined using the Nile Red fluorescence assay. In this method, small amounts of Nile Red are added to solutions containing increasing concentrations of the gelator. The dye, which is nonfluorescent in polar environments, exhibits a marked fluorescence enhancement and a blue shift when partitioned into hydrophobic domains. The CAC is identified as the concentration at which a sudden increase in fluorescence intensity or a shift in the λ_max_ (emission maximum) occurs, indicating the formation of hydrophobic microenvironments such as micelles or pregel aggregates.

Fluorescence measurements were carried out by using a Jasco FP-8200 spectrofluorometer (Tokyo, Japan). A Nile Red stock solution was prepared by dissolving 1 mg of Nile Red in 1 mL of acetone. For each measurement, 3 mL of the sample solution was transferred into a 1 cm × 1 cm quartz cuvette with four transparent sides, and 2.5 μL of the Nile Red stock solution was added directly to the cuvette. The excitation wavelength was set at 552 nm, and emission spectra were recorded in the 580–750 nm range. The scan speed was 100 nm/min, with both excitation and emission bandwidths set to 5 nm and a data interval of 0.5 nm.

### 4.6 Dynamic Light Scattering

DLS measurements were performed using a Zetasizer Nano ZS instrument (Malvern Panalytical). Measurements were carried out at 25 °C using 1.5 mL semimicro disposable PMMA cuvettes, purchased from Brand (Wertheim, Germany). Each sample was analyzed in triplicate, and the results were reported as the *Z*-Average hydrodynamic diameter and the Polydispersity Index (PdI), as calculated by the instrument software.

To verify whether aggregation occurred at the micrometric level, the same alkaline aqueous solutions of Adi-[Tyr­(Bn)]_2_ **4**, Pim-[Tyr­(Bn)]_2_ **5**, and Az-[Tyr­(Bn)]_2_ **6** previously used for the determination of the CAC were analyzed by DLS. Starting from the lowest tested concentrations, the formation of nanosized aggregates was detected at 0.06 mM for **4**, 0.1 mM for **5**, and 0.2 mM for **6**. These values correspond to the minimum concentrations at which the presence of nanoparticles could be observed.

Specifically, the DLS analysis revealed that

- Adi-[Tyr­(Bn)]_2_ at 0.06 mM formed nanoaggregates with an average hydrodynamic diameter of 186.2 nm (PdI = 0.433);
- Pim-[Tyr­(Bn)]_2_ at 0.1 mM formed nanoaggregates with an average size of 256.5 nm (PdI = 0.347);
- Az-[Tyr­(Bn)]_2_ at 0.2 mM formed nanoaggregates with an average size of 181.7 nm (PdI = 0.317).

### 4.7 Single-Crystal X-ray Diffraction

XRD data for compounds: Lau-Tyr­(Bn) **2**, Pal-Tyr­(Bn) **3**, and Pim-[Tyr­(Bn)]_2_ **5** were collected at room temperature and at 293 K (RT) on an Oxford X’Calibur S CCD diffractometer equipped with a graphite monochromator (Mo Kα radiation, λ = 0.71073 Å).

All the structures were solved with SHELXT by intrinsic phasing^66^ and refined on F^2^ with SHELXL^67^ implemented in the Olex2 software^68^ by full-matrix least-squares refinement. For all compounds, H_NH_ or H_OH_ atoms were directly located from the density maps or added in calculated positions, while all H_CH_ atoms were added in calculated positions and refined by riding on their respective carbon atoms.

All non-hydrogen atoms were anisotropically refined, and the rigid-body RIGU restraints^69^ applied. Data collection and refinement details are listed in Table S2. The Mercury^70^ and CylView programs were used to calculate intermolecular interactions and for molecular graphics, respectively. Crystal data can be obtained free of charge via www.ccdc.cam.ac.uk/conts/retrieving.html (or from the Cambridge Crystallographic Data Centre, 12 Union Road, Cambridge CB21EZ, UK; fax: (+44)­1223-336-033; or e-mail: deposit@ccdc.cam.ac.uk). CCDC numbers 2503038–2503040.

### 4.8 ECD/UV Analysis

ECD/UV spectra were recorded at room temperature by using a Jasco J-715 spectropolarimeter. For measurements on solutions, 0.01, 0.1, or 1 cm quartz cells were used, as indicated in the figure captions. Spectra were acquired at 50 nm/min from 400 to 185 nm, and an average of three scans was taken. For the hydrogels, a 0.001 cm demountable sandwich cell was used. Each hydrogel sample was measured in triplicate, and each measurement was performed in different orientations (front, back, and 90° rotation). For each hydrogel, the average of all collected spectra is reported.

## Supplementary Material





## Notes

Crystal data and refinement details for compounds: Lau-Tyr­(Bn) 2, (B) Pal-Tyr­(Bn) 3, and Pim-[Tyr­(Bn)]­2 5. Accession codes: 2503038−2503040 contain the supplementary crystallographic data for this paper. These data can be obtained free of charge via the joint Cambridge Crystallographic Data Centre (CCDC). (CIF)
Synthesis and Characterization of gelators **1–6** and IR-ATR, ^1^H NMR, and ^13^C NMR spectra and HPLC-MS analysis of compounds **1–6** (PDF)
