# Supplementary material for: Fine Tuning of Tyrosine-Derived Amphiphilic and Bolaamphiphilic Gelators for the Formation of pH-Responsive Supramolecular Gels
Source: Precis Chem. 2026 Jan 21;4(4):368–79. doi: 10.1021/prechem.5c00326 (PMC13126391; doi:10.1021/prechem.5c00326)
Supplement: Supplementary file 2 [file pc5c00326_si_002.pdf]

SUPPORTING INFORMATION

Fine Tuning of Tyrosine Derived Amphiphilic and Bolaamphiphilic Gelators for the Formation of pH-Responsive Supramolecular Gels

Fabia Cenciarelli,[a] Demetra Giuri,[a] Silvia Pieraccini,[a] Sofia Chinelli,[a] Devis Montroni,[a] Simone D'Agostino,[a*] Claudia Tomasini[a*]

[a] *Dipartimento di Chimica Giacomo Ciamician, Università di Bologna, Via Piero Gobetti, 85, 40129 Bologna, Italy*

email: claudia.tomasini@unibo.it; simone.dagostiono@unibo.it

**Scheme S1.** Reagents and conditions for the synthesis of gelators **1-3**	Page S3
**Scheme S2.** Reagents and conditions for the synthesis of gelators **4-6**	Page S4
Synthesis and Characterization of gelators **1-6**	Pages S5-S12
IR-ATR, ^{1}H NMR, ^{13}C NMR spectra and HPLC-MS analysis of compounds **1-6**	Pages S13-S30
**Table S1.** Minimum gelation concentration (MGC) values and concentration ranges tested for gelators **1-6**, determined by vial inversion method.	Page S31
**Figure S1.** Photographs of the trials for the measurement of the MGC: Adi-[Tyr(OBn)]$_2$ **4** and Aze-[Tyr(OBn)]$_2$ **6**	Page S31
**Figure S2.** Determination of the CAC with the Nile Red Fluorescence assay for compounds **4-6**	Page S32
**Figure S3.** Distribution by intensity of the particles formed in basic aqueous solutions of gelators **4-6**	Page S33
**Figure S4.** DLS correlation coefficient, number and volume analysis of particles from gelators **4-6**	Page S34
**Table S2.** Crystal data and refinement details for Lau-Tyr(Bn) **2,** Pal-Tyr(Bn) **3**, and Pim-[Tyr(Bn)]$_2$ **5** collected at RT.	Page S35
**Figure S5.** ECD/UV spectra, recorded in methanol at 0.5% w/v, for derivatives **1-3** and **4-6**.	Page S36

**Figure S6.** ECD/UV spectra, recorded in methanol on dilution, for derivatives **1-3** and **4-6**.	Page S37
**Figure S7.** ECD/UV spectra recorded on derivatives **4-6** at 0.5% w/v in methanol and alkaline water.	Page S38
**Figure S8.** (top) ^{1}H NMR spectrum after the addition of GdL (t = 0), (middle) ^{1}H NMR spectrum of the basic solution before addition of GdL, (bottom) ^{1}H NMR spectrum after the addition of GdL (t = 18 h). Only the signals due to GdL are visible.	Page S39

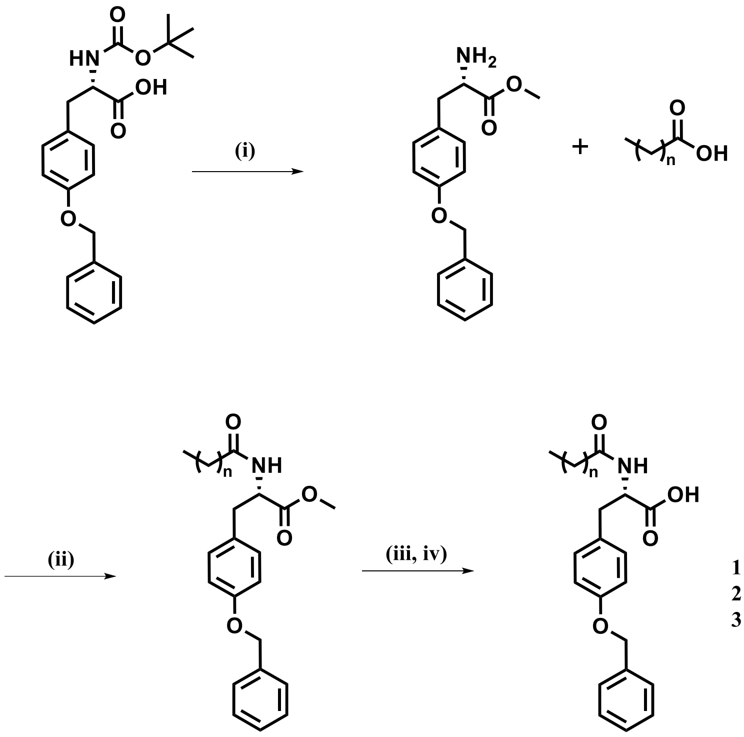

1: n = 6
2: n=10
3: n=14

1, 2, 3

Scheme S1. Reagents and conditions: (i) $SOCl_2$ (5.4 equiv.), MeOH, r.t., 24 h; (ii) fatty acid (n=6, 10, 14) (1 equiv.), HBTU (1.1 equiv.), DIEA (2.1 equiv.), dry ACN, r.t., 4 h; (iii) 1M NaOH (1.25 equiv.), MeOH/THF, r.t., 18 h; (iv) 1M HCl (1.35 equiv.), r.t., 10 min.

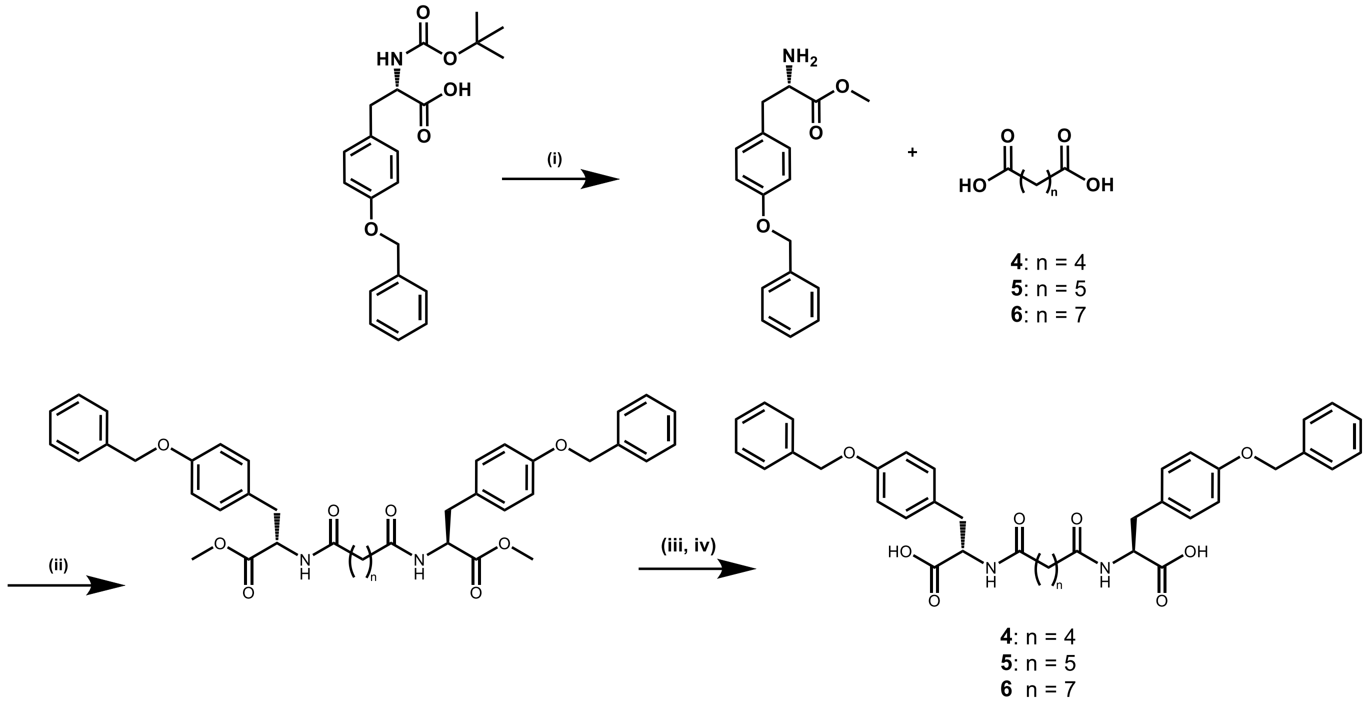

Scheme S2. Reagents and conditions: (i) $SOCl_2$ (5.4 equiv.), MeOH, r.t., 24 h; (ii) fatty acid (n= 4, 5, 7) (0.5 equiv.), HBTU (1.1 equiv.), DIEA (2.1 equiv.), dry ACN, r.t., 4 h; (iii) 1 M NaOH (2.5 equiv.), MeOH/THF, r.t., 18 h; (iv) 1 M HCl (2.7 equiv.), r.t., 10 min.

Synthesis of Cap-Tyr(Bn)-OMe - In a round-bottom flask containing methanol (MeOH) (2.6 mL) cooled to 0 °C, thionyl chloride ($SOCl_2$) (392 µL, 5.4 mmol) was added dropwise under continuous stirring. Following the complete addition of the thionyl chloride, Boc-L-Tyr(Bn)-OH (371.4 mg, 1 mmol) was introduced into the reaction mixture. The reaction was then allowed to proceed at room temperature for 24 h, ensuring constant stirring to maintain homogeneity throughout the reaction period. After 24 h, the reaction was complete, the solvent was removed under reduced pressure, and H-L-Tyr(Bn)-OMe was obtained in quantitative yield. In a three-neck round bottom flask the ottanoic acid (C8) (144.21 mg, 1 mmol) was dissolved in dry ACN (9.4 mL) and then *O*-(Benzotriazol-1-yl)-*N,N,N',N'*-tetramethyluronium hexafluorophosphate (HBTU) (416.9 mg, 1.1 mmol) was added. The mixture was stirred at room temperature for 10 minutes. A solution containing the H-L-Tyr(Bn)-OMe (285.4 mg, 1 mmol) and N,N-Diisopropylethylamine (DIEA) (0.530 mL, 3.1 mmol) in dry ACN (5.3 mL) was then added dropwise to the first one. The mixture was stirred at r.t. and under nitrogen atmosphere for 4h, then the solvent was removed under reduced pressure and replaced with CH_2Cl_2. The organic mixture was washed with H_2O, 1M aqueous HCl, aqueous $NaHCO_3$ sat., and brine, then it was dried over Na_2SO_4 and the solvent evaporated under vacuum. The residue was purified by silica gel column chromatography to give a white solid with a 83.9% yield. The column eluant was a mixture of cyclohexane:ethylacetate 4:1.White solid. Mp 79-82 °C; $[\alpha]_D$ +51.20 (c = 0.5 in DCM); IR-ATR: 3293, 2920, 2852, 1736, 1647, 1613, 1539, 1511 cm^{-1}; ^{1}H-NMR (600 MHz, $CDCl_3$) δ 0.87 (3H, t, J = 7.2 Hz, C8 CH_3), 1.25-1.31 (8H, m, C8 (CH_2)$_4$), 1.57-1.61 (2H, m, $COCH_2CH_2$), 2.16 (2H, t, J = 7.2 Hz, $COCH_2CH_2$), 3.06 (2H, ABX, J = 5.4, 14.4 Hz, Tyr CH_2Ph), 3.72 (3H, s, $COOCH_3$), 4.86 (1H, q, J = 7.8 Hz, COOC*H*), 5.03 (2H, s, Bn CH_2Ph), 5.83 (1H, d, J = 7.8 Hz, N*H*), 6.87-6.90 (2H, m, Tyr $H_{aromatic}$), 6.98-7.00 (2H, m, Tyr $H_{aromatic}$), 7.31-7.42 (5H, m, Bn $H_{aromatic}$); ^{13}C NMR (150 MHz, $CDCl_3$) δ 172.65, 172.28, 157.97, 136.95, 130.30, 128.61, 128.12, 128.01, 127.50, 114.93, 70.02, 53.01, 52.31, 37.10, 36.61, 31.68, 29.18, 28.99, 25.57, 22.62, 14.08; HPLC-MS(ESI): calc. 411.2; obs. 11.1 min; [(M+H$^+$)]: 412.3 m/z, [(M+Na$^+$)]: 434.3 m/z.

Synthesis of Cap-Tyr(Bn)-OH 1 - In a round bottom flask Cap-Tyr(Bn)-OMe (411.54 mg, 1 mmol) was dissolved in MeOH (1.7 mL) and THF (3.4 mL) under magnetic stirring. The whole system was placed in an ice bath in order to cool it down to 0 °C, then 1M aqueous solution of NaOH (1.25 mmol, 1.4 mL) was added. The reaction mixture was stirred at r.t. for 18h, then 1M aqueous solution of HCl (1.35 mmol, 1.5 mL) was added. The solvent was removed under reduced pressure and replaced with ethyl acetate. The organic mixture was washed with H_2O, then it was dried over Na_2SO_4 and the solvent evaporated under vacuum. A white solid was obtained (372.9 mg, 93.8% yield). Mp 127-131 °C; $[\alpha]_D$ +41.96 (c = 0.5 in EtOAc); IR-ATR: 3306, 2922, 2854, 1704, 1607, 1550, 1511 cm^{-1}; ^{1}H-NMR (600 MHz, $CDCl_3$) δ 0.87 (3H, t, J = 7.2 Hz, Pal CH_3), 1.25-1.30 (8H, m, C8 (CH_2)$_4$), 1.54-

1.59 (2H, m, $COCH_2CH_2$), 2.17 (2H, t, J = 7.8 Hz, $COCH_2CH_2$), 3.13 (2H, ABX, J = 6.0, 14.4 Hz, Tyr CH_2Ph), 4.80 (1H, q, J = 6.0 Hz, COOC*H*), 5.03 (2H, s, Bn CH_2Ph), 5.81 (1H, d, J = 7.2 Hz, N*H*), 6.90-6.93 (2H, m, Tyr $H_{aromatic}$), 7.07-7.09 (2H, m, Tyr $H_{aromatic}$), 7.31-7.42 (5H, m, Bn $H_{aromatic}$); ^{13}C NMR (150 MHz, CDCl3) δ 174.08, 173.29, 158.09, 136.88, 130.35, 128.61, 128.03, 127.80, 127.49, 115.08, 70.04, 53.40, 36.45, 36.19, 31.64, 29.10, 28.95, 25.48, 22.61, 14.08; HPLC-MS(ESI): calc. 397.2; obs. 9.0 min; [(M+H^+)]: 398.3 m/z, [(M+Na^+)]: 420.3 m/z.

Synthesis of Lau-Tyr(Bn)-OMe - In a round-bottom flask containing methanol (MeOH) (2.6 mL) cooled to 0 °C, thionyl chloride ($SOCl_2$) (392 μL, 5.4 mmol) was added dropwise under continuous stirring. Following the complete addition of the thionyl chloride, Boc-L-Tyr(Bn)-OH (371.4 mg, 1 mmol) was introduced into the reaction mixture. The reaction was then allowed to proceed at room temperature for 24 h, ensuring constant stirring to maintain homogeneity throughout the reaction period. After 24 h, the reaction was complete, the solvent was removed under reduced pressure, and H-L-Tyr(Bn)-OMe was obtained in quantitative yield. In a three-neck round bottom flask the lauric acid (Lau) (200 mg, 1 mmol) was dissolved in dry ACN (9 mL) and then *O*-(Benzotriazol-1-yl)-*N,N,N',N'*-tetramethyluronium hexafluorophosphate (HBTU) (416.9 mg, 1.1 mmol) was added. The mixture was stirred at room temperature for 10 minutes. A solution containing the H-L-Tyr(Bn)-OMe (285.4 mg, 1 mmol) and N,N-Diisopropylethylamine (DIEA) (0.560 mL, 3.3 mmol) in dry ACN (8 mL) was then added dropwise to the first one. The mixture was stirred at r.t. and under nitrogen atmosphere for 4 h, then the solvent was removed under reduced pressure and replaced with CH_2Cl_2. The organic mixture was washed with H_2O, 1M aqueous HCl, aqueous $NaHCO_3$ sat., and brine, then it was dried over Na_2SO_4 and the solvent evaporated under vacuum. The residue was purified by silica gel column chromatography to give a white solid with a 86.9% yield. The column eluant was a mixture of cyclohexane:ethylacetate 4:1. Mp 90-91°C; $[\alpha]_D$ +55.8° (c = 0.5 in DCM); IR-ATR: 3306, 2955, 2918, 2849, 1755, 1645, 1612, 1539, 1511, 1459 cm^{-1}; ^{1}H-NMR (400 MHz, $CDCl_3$) δ 0.86 (3H, t, J = 8.0 Hz, Lau CH_3), 1.24-1.28 (16H, m, Lau $(CH_2)_8$), 1.54-1.59 (2H, m, $COCH_2CH_2$), 2.15 (2H, t, J = 8.0 Hz, $COCH_2CH_2$), 3.04 (2H, ABX, J = 4.0, 8.0 Hz, Tyr CH_2Ph), 3.71 (3H, s, $COOCH_3$), 4.85 (1H, q, J = 8.0 Hz, COOC*H*), 5.01 (2H, s, Bn CH_2Ph), 5.87 (1H, d, J = 8.0 Hz, N*H*), 6.86-6.90 (2H, m, Tyr $H_{aromatic}$), 6.97-7.00 (2H, m, Tyr $H_{aromatic}$), 7.29-7.42 (5H, m, Bn $H_{aromatic}$); ^{13}C-NMR (100 MHz, $CDCl_3$) δ 172.63, 172.25, 157.93, 136.92, 130.26, 128.56, 128.11, 127.96, 127.46, 127.44, 114.89, 69.98, 53.00, 52.26, 37.07, 36.56, 31.89, 29.61, 29.59, 29.46, 29.32, 29.21, 25.55, 22.66, 14.10; HPLC-MS(ESI): calc. 467.3; obs. 15.1 min; [(M+H^+)]: 468.2, [(M+Na^+)]: 490.2 m/z.

Synthesis of Lau-Tyr(Bn)-OH, 2 - In a round bottom flask Lau-Tyr(Bn)-OMe (467.65mg, 1 mmol) was dissolved in MeOH (1.7 mL) and THF (3.5 mL) under magnetic stirring. The whole system was

placed in an ice bath in order to cool it down to 0 °C, then 1 M aqueous solution of NaOH (1.25 mmol, 1.4 mL) was added. The reaction mixture was stirred at r.t. for 18h, then 1 M aqueous solution of HCl (1.35 mmol, 1.5 mL) was added. The solvent was removed under reduced pressure and replaced with ethyl acetate. The organic mixture was washed with H_2O, then it was dried over Na_2SO_4 and the solvent evaporated under vacuum. A white solid was obtained with a 90.5% yield. Mp 122-124 °C; $[\alpha]_D$ +40.8° (c = 0.5 in EtOAc); IR-ATR: 3306, 2955, 2918, 2849, 1755, 1645, 1612, 1539, 1511, 1459, 1433 cm^{-1}; ^{1}H-NMR (400 MHz, $CDCl_3$) δ 0.86 (3H, t, J = 6.8 Hz, Lau CH_3), 1.24-1.28 (16H, m, Lau (CH_2)$_8$), 1.54-1.59 (2H, m, COCH$_2$CH_2), 2.13-2.19 (2H, m, COCH_2CH$_2$), 3.04 (2H, ABX, J = 5.6, 14.4 Hz, Tyr CH_2Ph), 3.71 (3H, s, COOCH_3), 4.85 (1H, q, J = 7.6 Hz, COOC*H*), 5.01 (2H, s, Bn CH_2Ph), 5.87 (1H, d, J = 7.6 Hz, N*H*), 6.86-6.90 (2H, m, Tyr $H_{aromatic}$), 6.97-7.00 (2H, m, Tyr $H_{aromatic}$), 7.29-7.42 (5H, m, Bn $H_{aromatic}$); ^{13}C-NMR (100 MHz, $CDCl_3$) δ 174.71, 174.50, 174.01, 158.00, 136.88, 130.39, 128.55, 127.96, 127.91, 127.46, 114.95, 69.97, 53.25, 36.45, 36.42, 31.89, 29.63, 29.61, 29.48, 29.33, 29.31, 29.17, 25.56, 22.66, 22.32, 14.10; HPLC-MS(ESI): calc. 453.2; obs. 12.4 min; [(M-H^+)]: 452.2 m/z.

Synthesis of Pal-Tyr(Bn)-OMe - In a round-bottom flask containing methanol (MeOH) (2.6 mL) cooled to 0°C, thionyl chloride ($SOCl_2$) (392 µL, 5.4 mmol) was added dropwise under continuous stirring. Following the complete addition of the thionyl chloride, Boc-L-Tyr(Bn)-OH (371.4 mg, 1 mmol) was introduced into the reaction mixture. The reaction was then allowed to proceed at room temperature for 24 h, ensuring constant stirring to maintain homogeneity throughout the reaction period. After 24 h, the reaction was complete, the solvent was removed under reduced pressure, and H-L-Tyr(Bn)-OMe was obtained in quantitative yield. In a three-neck round bottom flask the palmitic acid (Pal) (256.4 mg, 1 mmol) was dissolved in dry ACN (9 mL) and then *O*-(Benzotriazol-1-yl)-*N,N,N',N'*-tetramethyluronium hexafluorophosphate (HBTU) (416.9 mg, 1.1 mmol) was added. The mixture was stirred at room temperature for 10 minutes. A solution containing the H-L-Tyr(Bn)-OMe (285.4 mg, 1 mmol) and N,N-Diisopropylethylamine (DIEA) (0.560 mL, 3.3 mmol) in dry ACN (8 mL) was then added dropwise to the first one. The mixture was stirred at r.t. and under nitrogen atmosphere for 4h, then the solvent was removed under reduced pressure and replaced with CH_2Cl_2. The organic mixture was washed with H_2O, 1M aqueous HCl, aqueous $NaHCO_3$ sat., and brine, then it was dried over Na_2SO_4 and the solvent evaporated under vacuum. The residue was purified by silica gel column chromatography to give a white solid with a 87.9% yield. The column eluant was a mixture of cyclohexane:ethylacetate 4:1. White solid. Mp 93-94 °C; $[\alpha]_D$ +49.8° (c = 0.5 in DCM); IR-ATR: 3356, 2916, 2849, 1749, 1647, 1522, 1511, 1464 cm^{-1}; ^{1}H-NMR (400 MHz, $CDCl_3$) δ 0.86 (3H, t, J = 8.0 Hz, Pal CH_3), 1.24-1.28 (24H, m, Pal (CH_2)$_{12}$), 1.54-1.61 (2H, m, COCH$_2$CH_2), 2.15 (2H, t, J = 8.0 Hz, COCH_2CH$_2$), 3.05 (2H, ABX, J = 4.0, 8.0 Hz, Tyr CH_2Ph), 3.71 (3H, s, COOCH_3),

4.85 (1H, q, J = 8.0 Hz, COOC*H*), 5.01 (2H, s, Bn CH_2Ph), 5.90 (1H, d, J = 8.0 Hz, N*H*), 6.86-6.88 (2H, m, Tyr $H_{aromatic}$), 6.97-6.99 (2H, m, Tyr $H_{aromatic}$), 7.29-7.42 (5H, m, Bn $H_{aromatic}$); ^{13}C-NMR (100 MHz, $CDCl_3$) δ 172.67, 172.27, 157.91, 136.89, 130.27, 128.57, 128.10, 127.98, 127.47, 114.86, 69.95, 53.00, 52.28, 37.05, 36.56, 31.92, 29.70, 29.67, 29.65, 29.64, 29.49, 29.36, 29.35, 29.23, 25.57, 22.69, 22.51, 14.14; HPLC-MS(ESI): calc. 523.4; obs. 21.5 min [M+H$^+$]: 524.4 m/Z, [M+Na$^+$]: 547.1.

Synthesis of Pal-Tyr(Bn)-OH 3 - In a round bottom flask Pal-Tyr(Bn)-OMe (523.76 mg, 1 mmol) was dissolved in MeOH (1.7 mL) and THF (3.5 mL) under magnetic stirring. The whole system was placed in an ice bath in order to cool it down to 0 °C, then 1M aqueous solution of NaOH (1.25 mmol, 1.4 mL) was added. The reaction mixture was stirred at r.t. for 18h, then 1M aqueous solution of HCl (1.35 mmol, 1.5 mL) was added. The solvent was removed under reduced pressure and replaced with ethyl acetate. The organic mixture was washed with H_2O, then it was dried over Na_2SO_4 and the solvent evaporated under vacuum. A white solid was obtained with a 92.4% yield. Mp 118-120 °C; $[\alpha]_D$ +30.2° (c = 0.5 in EtOAc); IR-ATR: 3302, 2918, 2849, 1727, 1643, 1533, 1511, 1468, 1442 cm^{-1}; ^{1}H-NMR (400 MHz, $CDCl_3$) δ 0.86 (3H, t, J = 6.4 Hz, Pal CH_3), 1.24-1.28 (24H, m, Pal (CH_2)$_{12}$), 1.54-1.61 (2H, m, $COCH_2CH_2$), 2.15 (2H, t, J = 8.0 Hz, $COCH_2CH_2$), 3.05 (2H, ABX, J = 5.6, 14.0 Hz, Tyr CH_2Ph), 3.71 (3H, s, COOCH_3), 4.85 (1H, q, J = 6.8 Hz, COOC*H*), 5.01 (2H, s, Bn CH_2Ph), 5.90 (1H, d, J = 7.6 Hz, N*H*), 6.86-6.88 (2H, m, Tyr $H_{aromatic}$), 6.97-6.99 (2H, m, Tyr $H_{aromatic}$), 7.29-7.42 (5H, m, Bn $H_{aromatic}$) ; ^{13}C-NMR (100 MHz, $CDCl_3$) δ 174.76, 174.00, 158.00, 136.88, 130.39, 128.54, 127.95, 127.92, 127.45, 114.95, 69.97, 53.27, 36.45, 36.42, 31.91, 29.70, 29.68, 29.65, 29.50, 29.34, 29.32, 29.18, 25.56, 22.67, 22.25, 14.10; HPLC-MS(ESI): calc. 509.3; obs. 22.2 min; [(M-H$^+$)]: 508.3 m/z.

Synthesis of Adi-[Tyr(Bn)-OMe]$_2$ - In a round-bottom flask containing methanol (MeOH) (2.6 mL) cooled to 0 °C, thionyl chloride ($SOCl_2$) (392 μL, 5.4 mmol) was added dropwise under continuous stirring. Following the complete addition of the thionyl chloride, Boc-L-Tyr(Bn)-OH (371.4 mg, 1 mmol) was introduced into the reaction mixture. The reaction was then allowed to proceed at room temperature for 24 h, ensuring constant stirring to maintain homogeneity throughout the reaction period. After 24 h, the reaction was complete, the solvent was removed under reduced pressure, and H-L-Tyr(Bn)-OMe was obtained in quantitative yield. In a three-neck round bottom flask the adipic acid (C6) (73.1 mg, 0.5 mmol) was dissolved in dry ACN (7.3 mL) and then *O*-(Benzotriazol-1-yl)-*N,N,N',N'*-tetramethyluronium hexafluorophosphate (HBTU) (416.9 mg, 1.1 mmol) was added. The mixture was stirred at room temperature for 10 minutes. A solution containing the H-L-Tyr(Bn)-OMe (286 mg, 1 mmol) and N,N-Diisopropylethylamine (DIEA) (0.560 mL, 3.3 mmol) in dry ACN (5.2

mL) was then added dropwise to the first one. The mixture was stirred at r.t. and under nitrogen atmosphere for 4h, then the solvent was removed under reduced pressure and replaced with CH_2Cl_2. The organic mixture was washed with H_2O, 1M aqueous HCl, aqueous $NaHCO_3$ sat., and brine, then it was dried over Na_2SO_4 and the solvent evaporated under vacuum. The residue was purified by silica gel column chromatography to give a white solid (526.3 mg, 77.3% yield). The column eluant was a mixture of cyclohexane:ethylacetate 3:7. Mp 141-146 °C; $[\alpha]_D$ +46.69° (c = 0.5 in DCM); IR-ATR: 3309, 3066, 3030, 2945, 2928, 2858, 1731, 1720, 1636, 1612, 1540, 1534, 1511 cm^{-1}; ^{1}H-NMR (600 MHz, $CDCl_3$) δ 1.63-1.65 (4H, m, C6 2COCH$_2$C*H$_2$*), 2.19-2.22 (4H, m, 2COC*H$_2$*CH$_2$), 3.07 (4H, ABX, J = 6.0, 13.8 Hz, Tyr C*H$_2$*Ph), 3.74 (6H, s, 2COOC*H$_3$*), 4.84-4.88 (2H, m, 2COOC*H*), 5.05 (4H, s, Bn 2C*H$_2$*Ph), 6.07 (2H, d, J= 7.8 Hz ,2N*H*), 6.90-6.93 (4H, m, Tyr $H_{aromatic}$), 7.02-7.04 (4H, m, Tyr $H_{aromatic}$), 7.33-7.45 (10H, m, Bn $H_{aromatic}$); ^{13}C NMR (150 MHz, $CDCl_3$) δ 172.38, 172.30, 157.95, 136.96, 130.25, 128.60, 128.17, 127.99, 127.51, 114.97, 70.01, 53.20, 52.33, 37.00, 35.88, 24.66; HPLC-MS(ESI): calc. 680.3; obs. 10.3 min; [(M+H$^+$)]: 681.3 m/z, [(M+Na$^+$)]: 703.3 m/z.

Synthesis of Adi-[Tyr(Bn)-OH]$_2$ 4: In a round bottom flask Adi-[Tyr(Bn)-OMe]$_2$ (680.8 mg, 1 mmol) was dissolved in MeOH (1.7 mL) and THF (3.5 mL) under magnetic stirring. The whole system was placed in an ice bath in order to cool it down to 0 °C, then 1 M aqueous solution of NaOH (2.5 mmol,) was added. The reaction mixture was stirred at r.t. for 18 h, then 1 M aqueous solution of HCl (2.7 mmol) was added. The solvent was removed under reduced pressure and replaced with ethyl acetate. The organic mixture was washed with H_2O, then it was dried over Na_2SO_4 and the solvent evaporated under vacuum. A white solid was obtained (507.8 mg, 77.8% yield). Mp 169-172 °C; $[\alpha]_D$ +26.60° (c = 0.5 in MeOH); IR-ATR: 3344, 3324, 3036, 2939, 2922, 2862, 1727, 1701, 1609 1584, 1540, 1534, 1508 cm^{-1}; ^{1}H-NMR (600 MHz, DMSO) δ 1.32-1.34 (4H, m, C6 2COCH$_2$C*H$_2$*), 1.98-2.03 (4H, m, 2COC*H$_2$*CH$_2$), 2.87 (4H, ABX, J = 4.8, 13.8 Hz, Tyr C*H$_2$*Ph), 4.35-4.39 (2H, m, 2COOC*H*), 5.05 (4H, s, Bn 2C*H$_2$*Ph), 6.89-6.91 (4H, m, Tyr $H_{aromatic}$), 7.11-7.14 (4H, m, Tyr $H_{aromatic}$), 7.31-7.44 (10H, m, Bn $H_{aromatic}$), 8.04 (2H, d, J= 8.4 Hz ,2N*H*), 12.60 (2H, s, 2COO*H*); ^{13}C NMR (150 MHz, DMSO) δ 206.96, 173.71, 172.45, 157.46, 137.67, 130.56, 130.27, 128.87, 128.23, 128.13, 114.90, 69.58, 55.39, 54.00, 36.44, 35.25, 31.17, 25.12; HPLC-MS(ESI): calc. 652.3; obs. 8.5 min; [(M+H$^+$)]: 653.3 m/z, [(M+Na$^+$)]: 675.2 m/z.

Synthesis of Pim-[Tyr(Bn)-OMe]$_2$ - In a round-bottom flask containing methanol (MeOH) (2.6 mL) cooled to 0 °C, thionyl chloride ($SOCl_2$) (392 µL, 5.4 mmol) was added dropwise under continuous stirring. Following the complete addition of the thionyl chloride, Boc-L-Tyr(Bn)-OH (371.4 mg, 1 mmol) was introduced into the reaction mixture. The reaction was then allowed to proceed at room temperature for 24 h, ensuring constant stirring to maintain homogeneity throughout the reaction

period. After 24 h, the reaction was complete, the solvent was removed under reduced pressure, and H-L-Tyr(Bn)-OMe was obtained in quantitative yield. In a three-neck round bottom flask the pimelic acid (C7) (80.1 mg, 0.5 mmol) was dissolved in dry ACN (9.4 mL) and then *O*-(Benzotriazol-1-yl)-*N,N,N',N'*-tetramethyluronium hexafluorophosphate (HBTU) (416.9 mg, 1.1 mmol) was added. The mixture was stirred at room temperature for 10 minutes. A solution containing the H-L-Tyr(Bn)-OMe (285.4 mg, 1 mmol) and N,N-Diisopropylethylamine (DIEA) (0.560 mL, 3.3 mmol) in dry ACN (5.2 mL) was then added dropwise to the first one. The mixture was stirred at r.t. and under nitrogen atmosphere for 4h, then the solvent was removed under reduced pressure and replaced with CH_2Cl_2. The organic mixture was washed with H_2O, 1M aqueous HCl, aqueous $NaHCO_3$ sat., and brine, then it was dried over Na_2SO_4 and the solvent evaporated under vacuum. The residue was purified by silica gel column chromatography to give a white solid (565.6 mg, 81.4% yield). The column eluant was a mixture of cyclohexane:ethylacetate 3:7. Mp 133-137 °C; $[\alpha]_D$ +60.88° (c = 0.5 in DCM); IR-ATR: 3308, 2930, 2861, 1733, 1639, 1610, 1530, 1510 cm^{-1}; ^1H-NMR (600 MHz, $CDCl_3$) δ 1.27-1.32 (2H, m, C7 (C*H*₂)), 1.59-1.63 (4H, m, 2COCH₂C*H*₂), 2.17 (4H, t, J = 7.2 Hz, 2COC*H*₂CH₂), 3.04 (4H, ABX, J = 4.8, 13.8 Hz, Tyr 2C*H*₂Ph), 3.71 (6H, s, 2COOC*H*₃), 4.85 (2H, q, J = 7.8 Hz, 2COOC*H*), 5.03 (4H, s, Bn 2C*H*₂Ph), 5.94 (2H, d, J = 8.4 Hz, 2N*H*), 6.88-6.90 (4H, m, Tyr $H_{aromatic}$), 6.99-7.01 (4H, m, Tyr $H_{aromatic}$), 7.31-7.43 (10H, m, Bn $H_{aromatic}$); ^{13}C NMR (150 MHz, $CDCl_3$) δ 172.49, 172.34, 157.95, 136.95, 130.27, 128.60, 128.15, 128.00, 127.51, 114.97, 70.02, 53.07, 52.32, 37.07, 36.18, 28.58, 25.01; HPLC-MS(ESI): calc.694.3; obs. 10.5 min; [(M+H^+)]: 695.3 m/z, [(M+Na^+)]: 717.3 m/z.

Synthesis of Pim-[Tyr(Bn)-OH]$_2$ 6 - In a round bottom flask Pim-[Tyr(Bn)-OMe]$_2$ (694.8 mg, 1 mmol) was dissolved in MeOH (1.8 mL) and THF (3.5 mL) under magnetic stirring. The whole system was placed in an ice bath in order to cool it down to 0 °C, then 1 M aqueous solution of NaOH (2.5 mmol, 2.8 mL) was added. The reaction mixture was stirred at r.t. for 18 h, then 1 M aqueous solution of HCl (2.7 mmol, 3.0 mL) was added. The solvent was removed under reduced pressure and replaced with ethyl acetate. The organic mixture was washed with H_2O, then it was dried over Na_2SO_4 and the solvent evaporated under vacuum. A white solid was obtained (595.4 mg, 89.3% yield). Mp 174-178 °C; $[\alpha]_D$ +25.10° (c = 0.5 in MeOH); IR-ATR: 3313, 2942, 2851, 1709, 1646, 1611, 1583, 1529, 1512 cm^{-1}; ^1H-NMR (600 MHz, DMSO) δ 1.04-1.09 (2H, m, C7 (C*H*₂)), 1.37 (4H, p, J= 7.2 Hz, 2COCH₂C*H*₂), 2.01 (4H, t, J = 7.4 Hz, 2COC*H*₂CH₂), 2.87 (4H, ABX, J = 4.8, 13.8 Hz, Tyr C*H*₂Ph), 4.34-4.38 (2H, m, 2COOC*H*), 5.03 (4H, s, Bn 2C*H*₂Ph), 6.89-6.91 (4H, m, Tyr $H_{aromatic}$), 7.12-7.14 (4H, m, Tyr $H_{aromatic}$), 7.31-7.44 (10H, m, Bn $H_{aromatic}$), 8.04 (2H, d, J= 8.2 Hz, 2N*H*), 12.60 (2H, s, 2COO*H*); ^{13}C NMR (150 MHz, DMSO) δ 173.74, 172.58, 157.45, 137.66, 130.56, 130.31,

128.87, 128.23, 128.13, 114.88, 69.58, 54.00, 36.43, 35.43, 28.53, 25.41; HPLC-MS(ESI): calc.666.3; obs. 8.6 min; $[(M+H^+)]$: 667.2 m/z, $[(M+Na^+)]$: 689.3 m/z.

Synthesis of Aze-[Tyr(Bn)-OMe]$_2$ - In a round-bottom flask containing methanol (MeOH) (2.6 mL) cooled to 0 °C, thionyl chloride ($SOCl_2$) (392 µL, 5.4 mmol) was added dropwise under continuous stirring. Following the complete addition of the thionyl chloride, Boc-L-Tyr(Bn)-OH (371.4 mg, 1 mmol) was introduced into the reaction mixture. The reaction was then allowed to proceed at room temperature for 24 h, ensuring constant stirring to maintain homogeneity throughout the reaction period. After 24 h, the reaction was complete, the solvent was removed under reduced pressure, and H-L-Tyr(Bn)-OMe was obtained in quantitative yield. In a three-neck round bottom flask the azelaic acid (Az) (94.1 mg, 0.5 mmol) was dissolved in dry ACN (9 mL) and then *O*-(Benzotriazol-1-yl)-*N,N,N',N'*-tetramethyluronium hexafluorophosphate (HBTU) (416.9 mg, 1.1 mmol) was added. The mixture was stirred at room temperature for 10 minutes. A solution containing the H-L-Tyr(Bn)-OMe (285.4 mg, 1 mmol) and N,N-Diisopropylethylamine (DIEA) (0.560 mL, 3.3 mmol) in dry ACN (8 mL) was then added dropwise to the first one. The mixture was stirred at r.t. and under nitrogen atmosphere for 4h, then the solvent was removed under reduced pressure and replaced with CH_2Cl_2. The organic mixture was washed with H_2O, 1M aqueous HCl, aqueous $NaHCO_3$ sat., and brine, then it was dried over Na_2SO_4 and the solvent evaporated under vacuum. The residue was purified by silica gel column chromatography to give a white solid with a 86.9% yield. The column eluant was a mixture of cyclohexane:ethylacetate 1:1. Mp 136-137 °C; $[\alpha]_D$ +66.0° (c = 0.5 in DCM); IR-ATR: 3309, 2924, 2851, 1738, 1645, 1511, 2344, 1457 cm^{-1}; ^{1}H-NMR (400 MHz, $CDCl_3$) δ 1.24-1.27 (6H, m, Az $(CH_2)_3$), 1.53-1.58 (4H, m, $2COCH_2CH_2$), 2.14 (4H, t, J = 8.0 Hz, $2COCH_2CH_2$), 3.03 (4H, ABX, J = 4.0, 8.0 Hz, Tyr $2CH_2Ph$), 3.69 (6H, s, $2COOCH_3$), 4.83 (2H, q, J = 8.0 Hz, 2COOC*H*), 5.01 (4H, s, Bn $2CH_2Ph$), 5.96 (2H, d, J = 8.0 Hz, 2N*H*), 6.86-6.89 (4H, m, Tyr $H_{aromatic}$), 6.97-7.01 (4H, m, Tyr $H_{aromatic}$), 7.28-7.41 (10H, m, Bn $H_{aromatic}$); ^{13}C-NMR (100 MHz, $CDCl_3$) δ 172.59, 172.28, 157.91, 136.93, 130.25, 128.57, 128.15, 127.96, 127.46, 114.90, 69.97, 53.05, 52.27, 37.06, 36.36, 29.67, 28.89, 25.36, 22.76, 22.74; HPLC-MS(ESI): calc. 722.3; obs. 10.5 min; $[(M+K^++H^+)/2]$: 381.2 m/z.

Synthesis of Aze-[Tyr(Bn)-OH]$_2$ 6 - In a round bottom flask Aze-[Tyr(Bn)-OMe]$_2$ (722.88 mg, 1 mmol) was dissolved in MeOH (1.8 mL) and THF (3.5 mL) under magnetic stirring. The whole system was placed in an ice bath in order to cool it down to 0 °C, then 1M aqueous solution of NaOH (2.5 mmol, 2.8 mL) was added. The reaction mixture was stirred at r.t. for 18h, then 1M aqueous solution of HCl (2.7 mmol, 3.0 mL) was added. The solvent was removed under reduced pressure and replaced with ethyl acetate. The organic mixture was washed with H_2O, then it was dried over

Na_2SO_4 and the solvent evaporated under vacuum. A white solid was obtained with a 94.5% yield. Mp 160-162 °C; $[\alpha]_D$ +26.9° (c = 0.5 in MeOH); IR-ATR: 3321, 2927, 2853, 1707, 1647, 1509, 1453 cm^{-1}; ^{1}H-NMR (400 MHz, CD_3OD) δ 1.09-1.19 (6H, m, Az $(CH_2)_3$), 1.40-1.47 (4H, m, $2COCH_2CH_2$), 2.10 (4H, t, J = 7.2 Hz, $2COCH_2CH_2$), 2.83 (2H, ABX, J = 4.4, 14.0 Hz, Tyr CH_2Ph), 3.12 (2H, ABX, J = 4.8, 14.0 Hz, Tyr CH_2Ph), 4.60 (2H, q, J = 4.8 Hz, 2COOC*H*), 4.97 (4H, s, Bn $2CH_2$Ph), 6.84-6.87 (4H, m, Tyr $H_{aromatic}$), 7.01-7.10 (4H, m, Tyr $H_{aromatic}$), 7.22-7.37 (10H, m, Bn $H_{aromatic}$); ^{13}C-NMR (100 MHz, CD_3OD) δ 174.67, 173.53, 157.69, 137.34, 129.85, 129.33, 128.07, 127.41, 127.10, 114.46, 69.54, 53.60, 47.38, 47.17, 46.96, 36.20, 35.31, 28.54, 28.40, 25.34, 18.21; HPLC-MS(ESI): calc. 694.3; obs. 8.5 min; $[(M-H^+)]$: 693.3 m/z.

IR spectrum of **Cap-Tyr(Bn) 1**

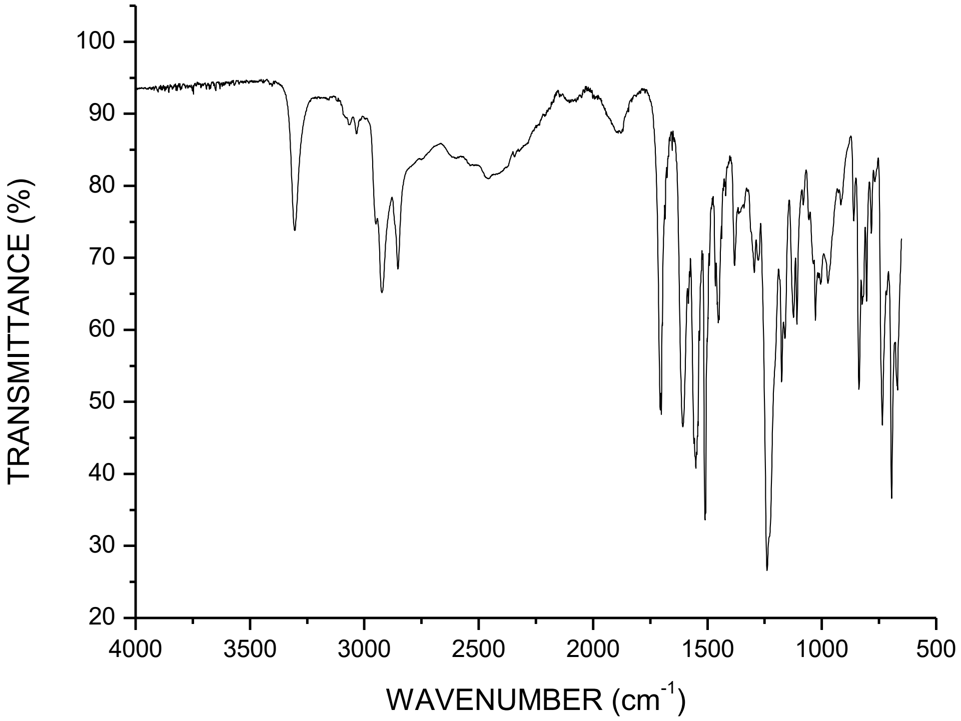

^{1}H NMR spectrum of **Cap-Tyr(Bn) 1** ($CDCl_3$)

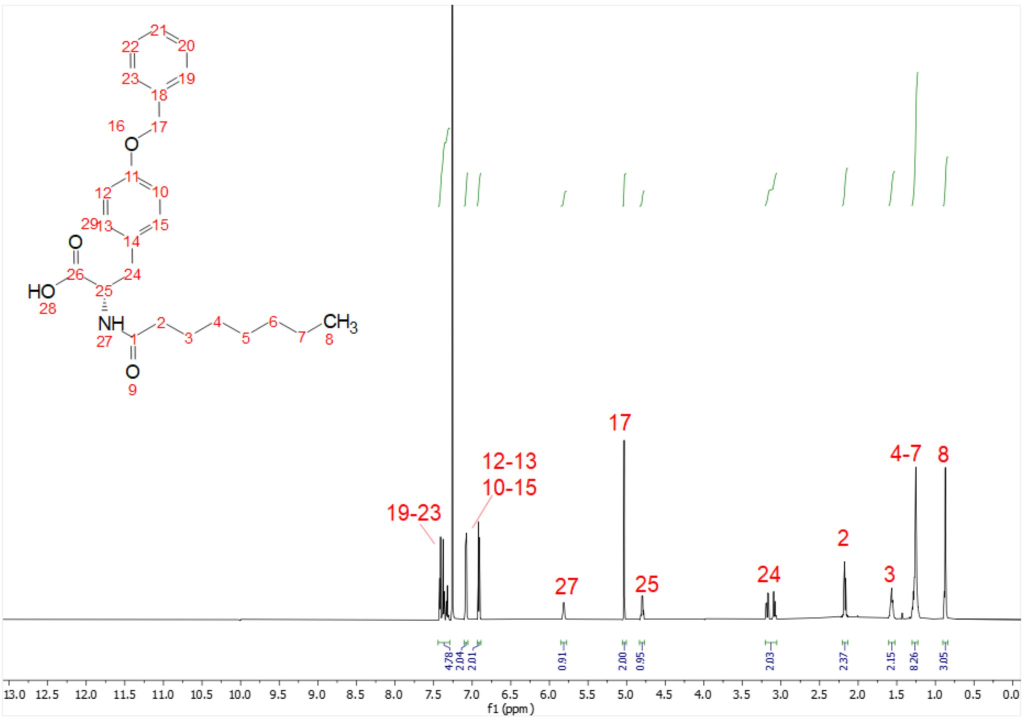

^{13}C NMR spectrum of **Cap-Tyr(Bn) 1** ($CDCl_3$)

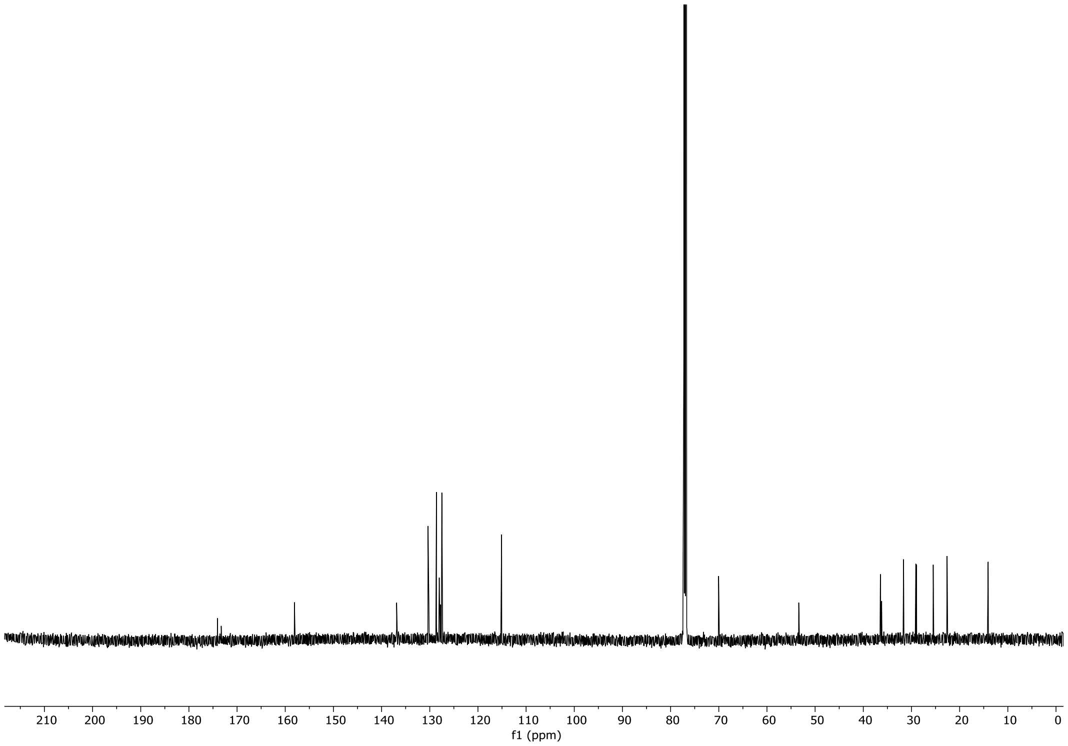

COSY spectrum of **Cap-Tyr(Bn) 1**

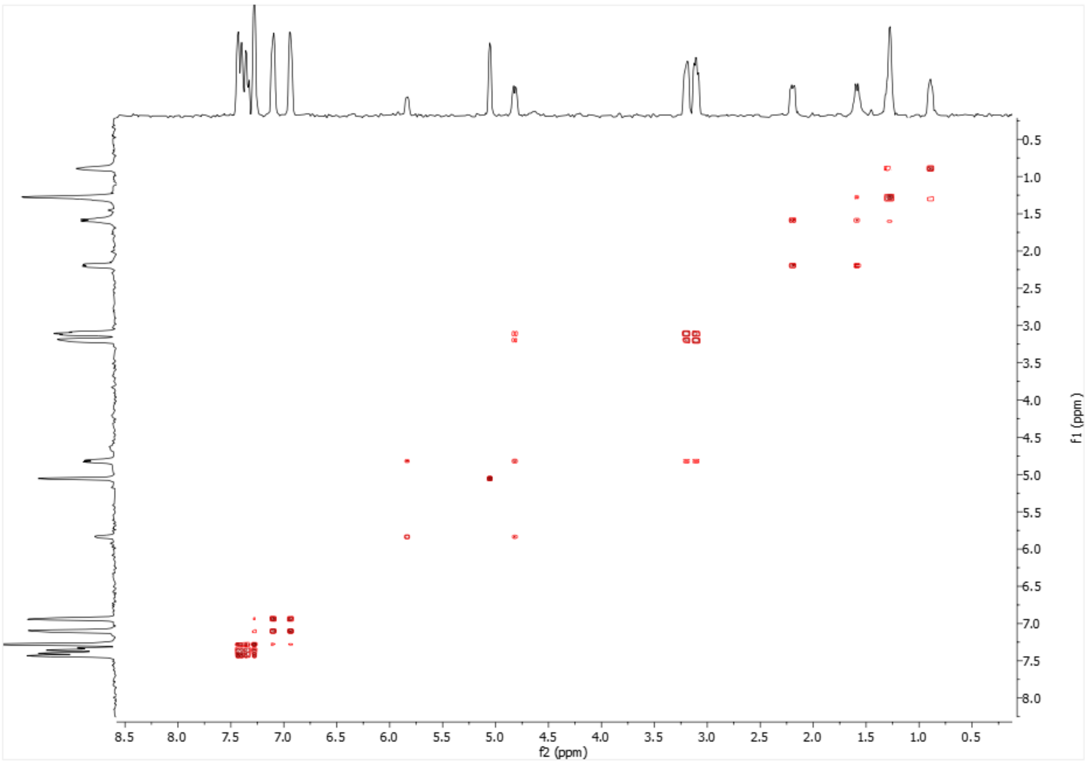

HPLC-MS of **Cap-Tyr(Bn) 1**

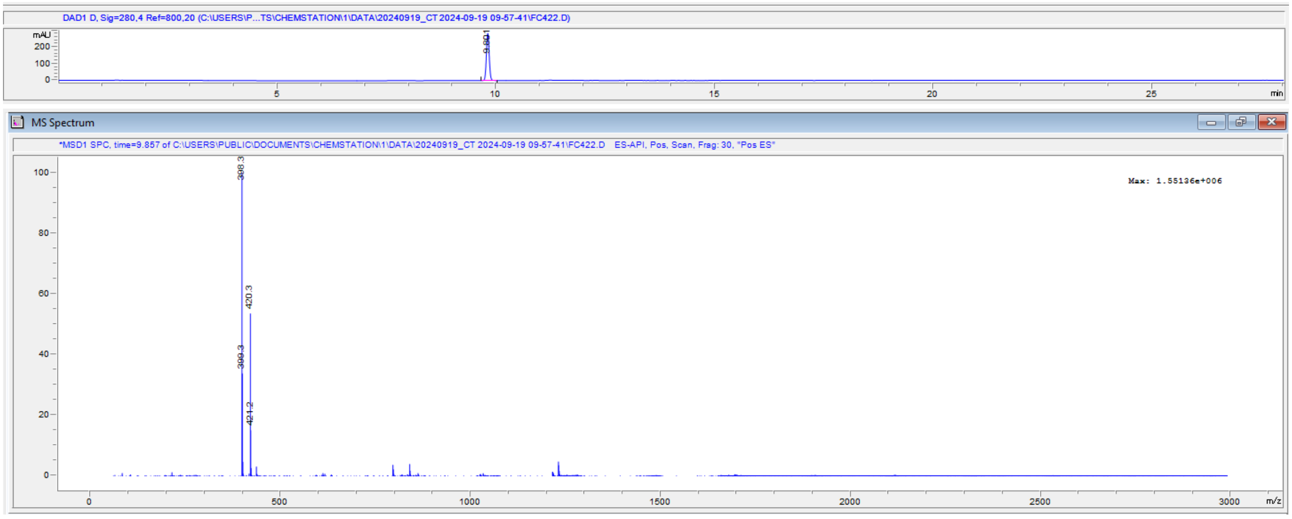

IR spectrum of **Lau-Tyr(Bn) 2**

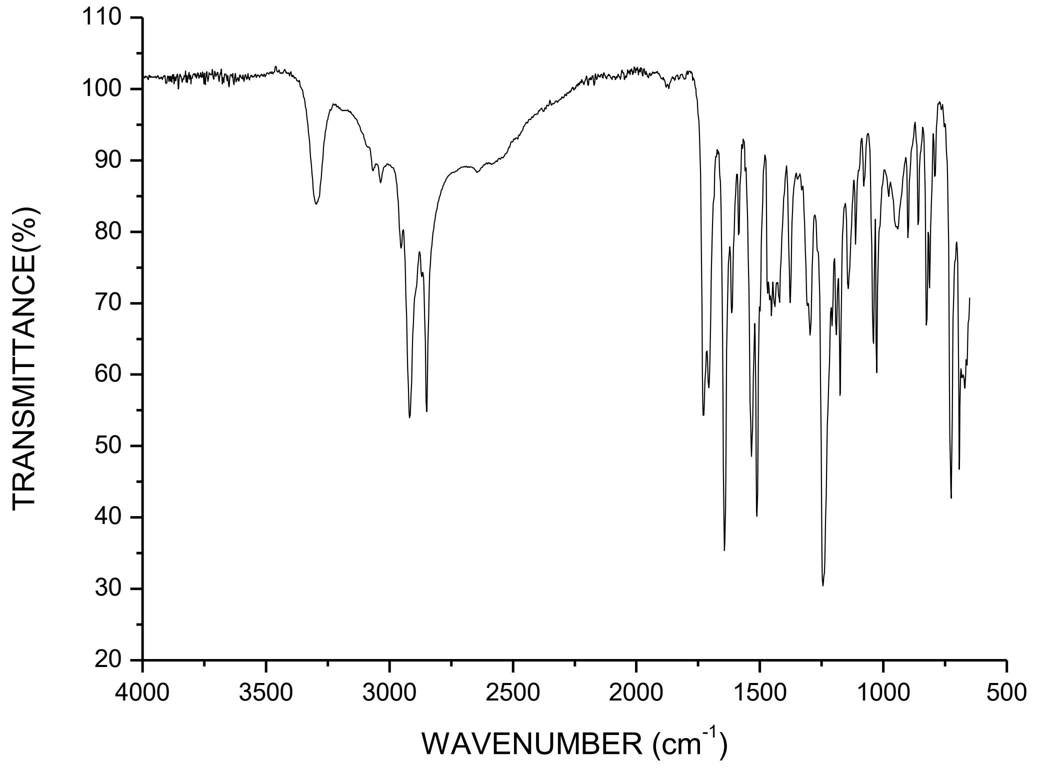

^{1}H-NMR spectrum of **Lau-Tyr(Bn) 2** ($CDCl_3$)

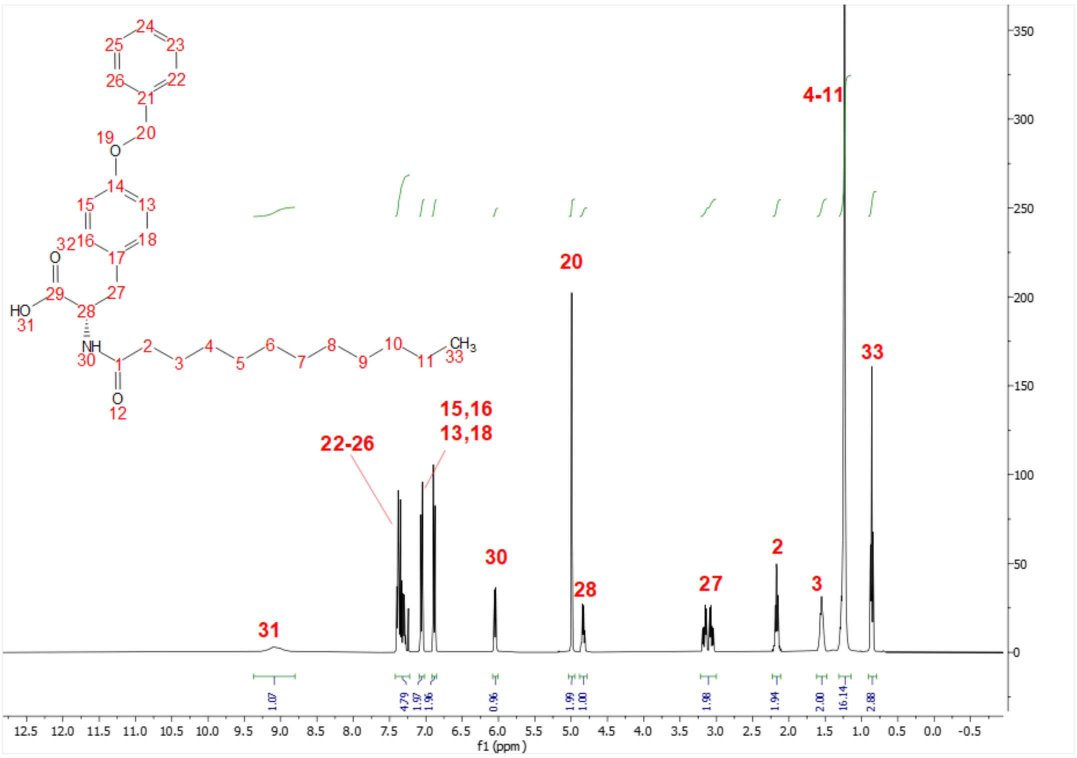

^{13}C-NMR spectrum of **Lau-Tyr(Bn) 2** ($CDCl_3$)

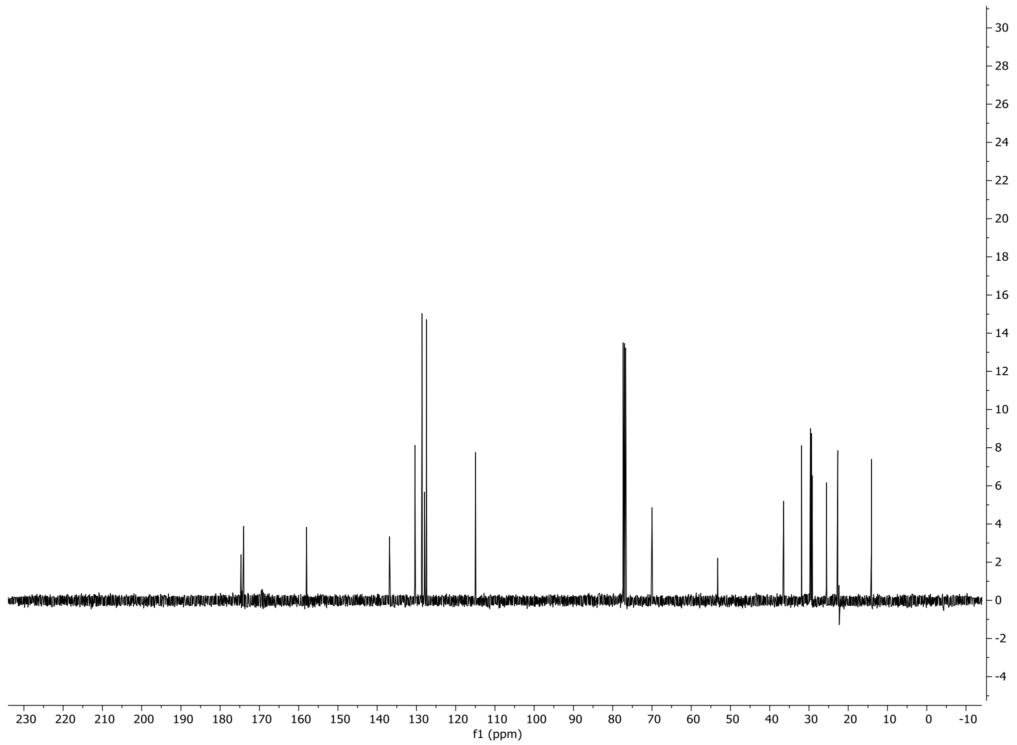

COSY spectrum of **Lau-Tyr(Bn) 2** ($CDCl_3$)

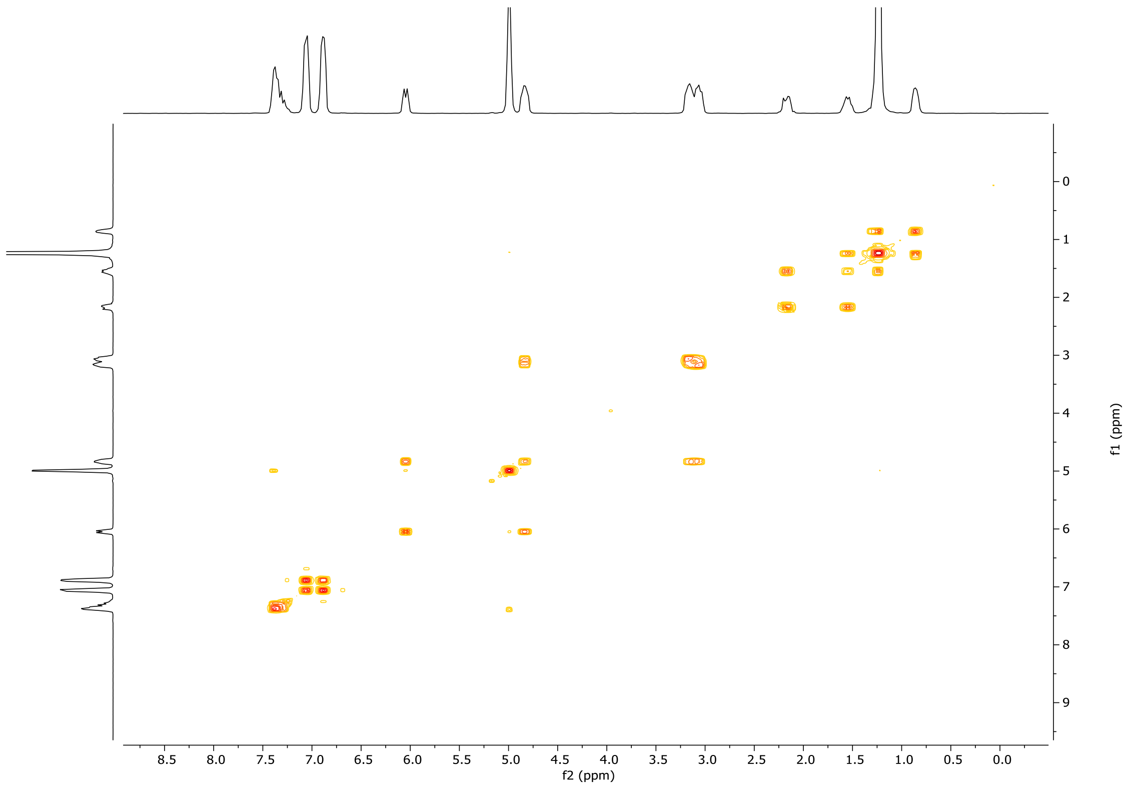

HPLC-MS of **Lau-Tyr(Bn) 2**

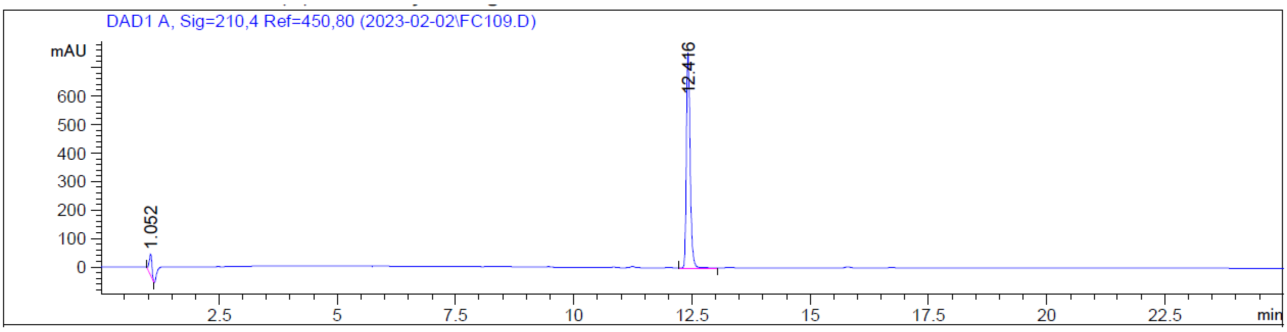

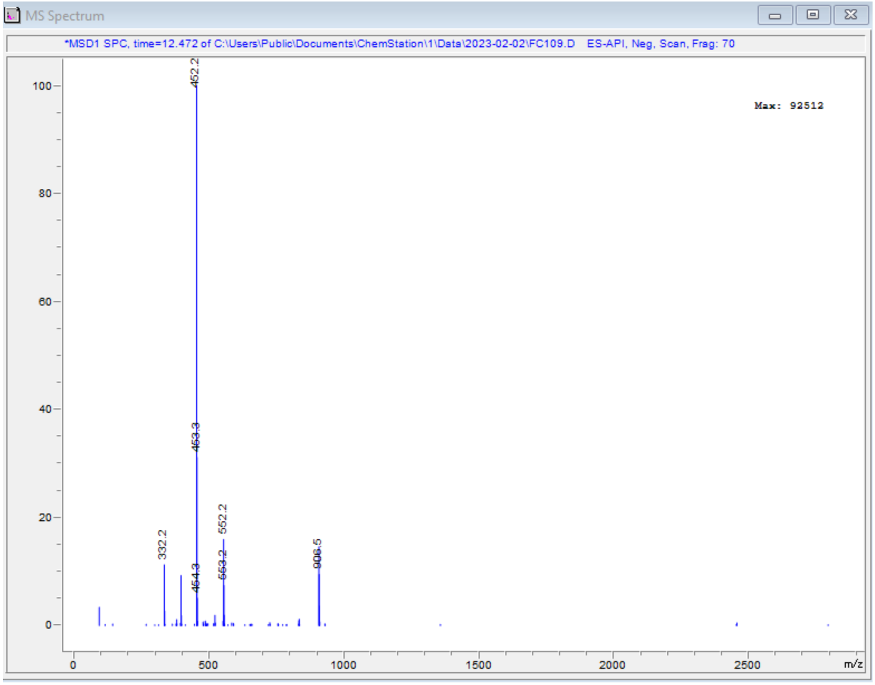

IR spectrum of **Pal-Tyr(Bn) 3** ($CDCl_3$)

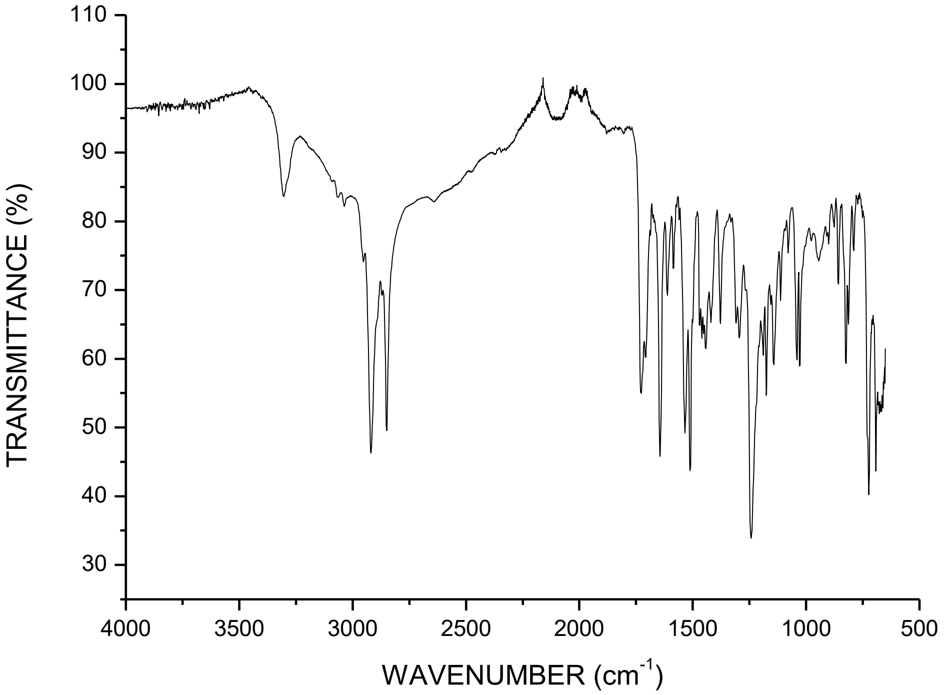

^{1}H-NMR spectrum of **Pal-Tyr(Bn) 3** ($CDCl_3$)

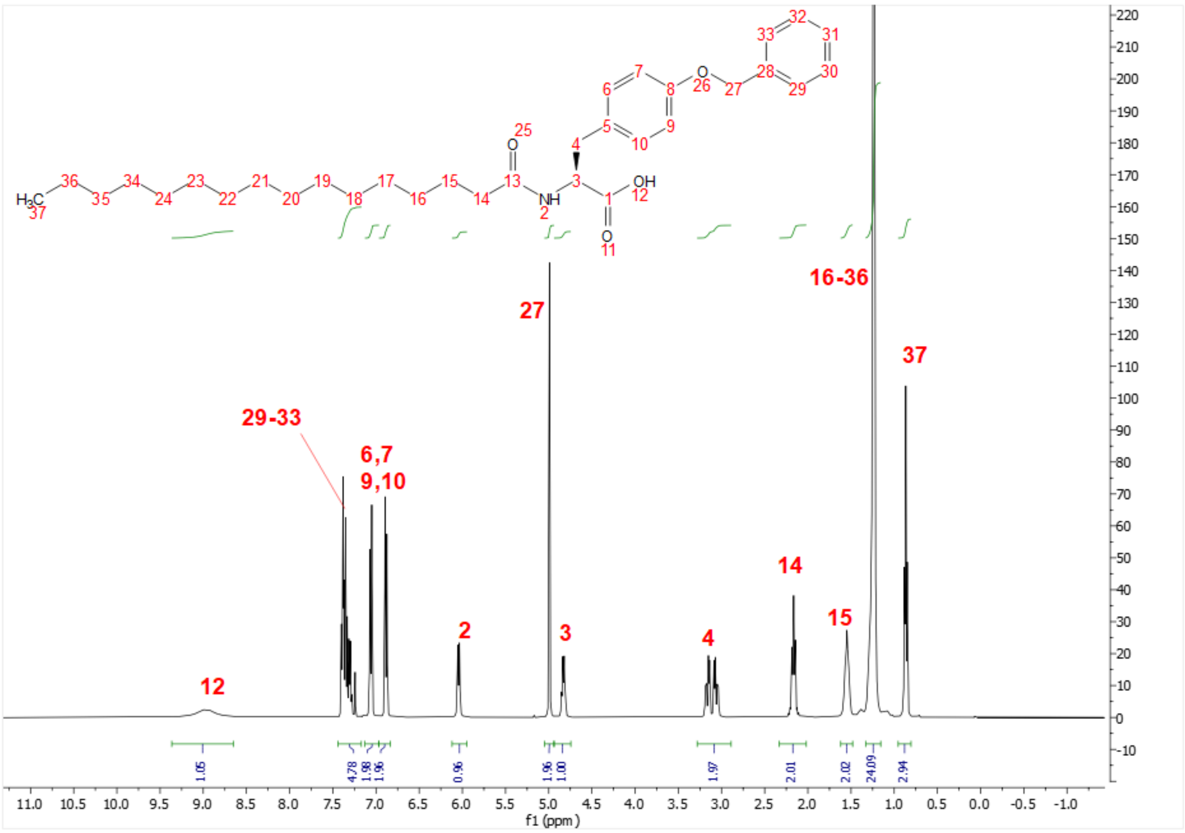

^{13}C-NMR spectrum of **Pal-Tyr(Bn) 3** (CDCl$_3$)

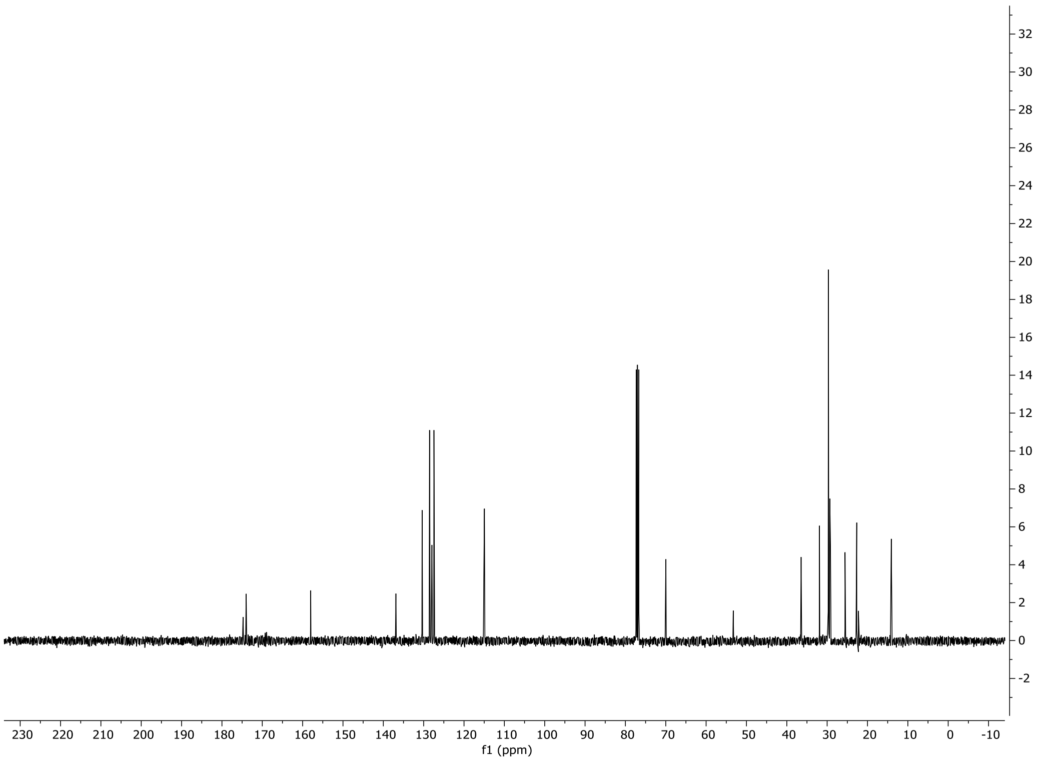

COSY spectrum of **Pal-Tyr(Bn) 3** (CDCl$_3$)

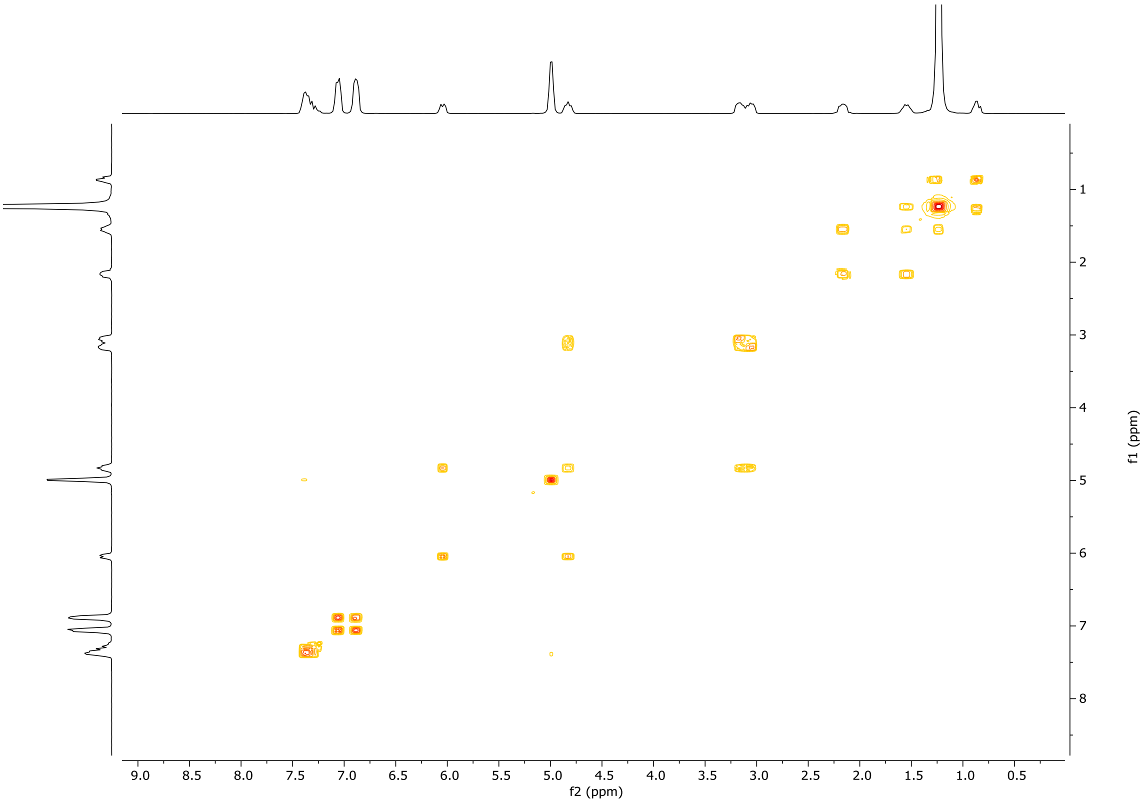

HPLC-MS of **Pal-Tyr(Bn) 3**

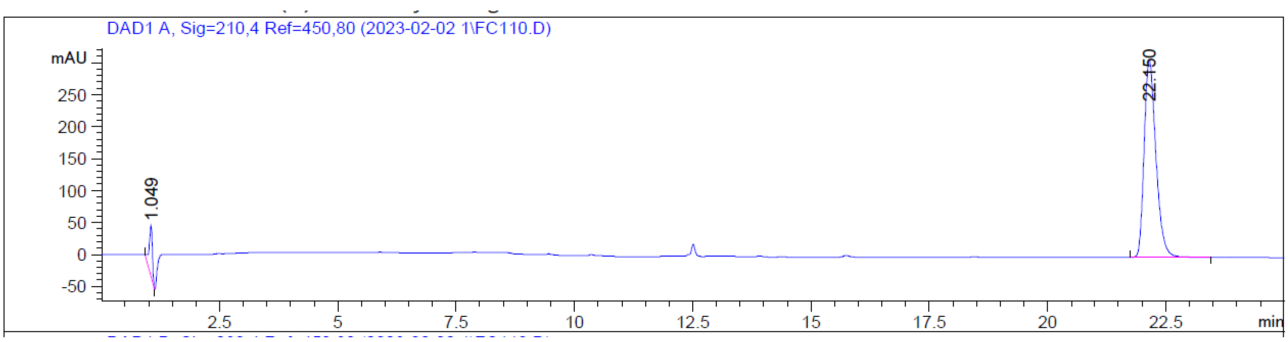

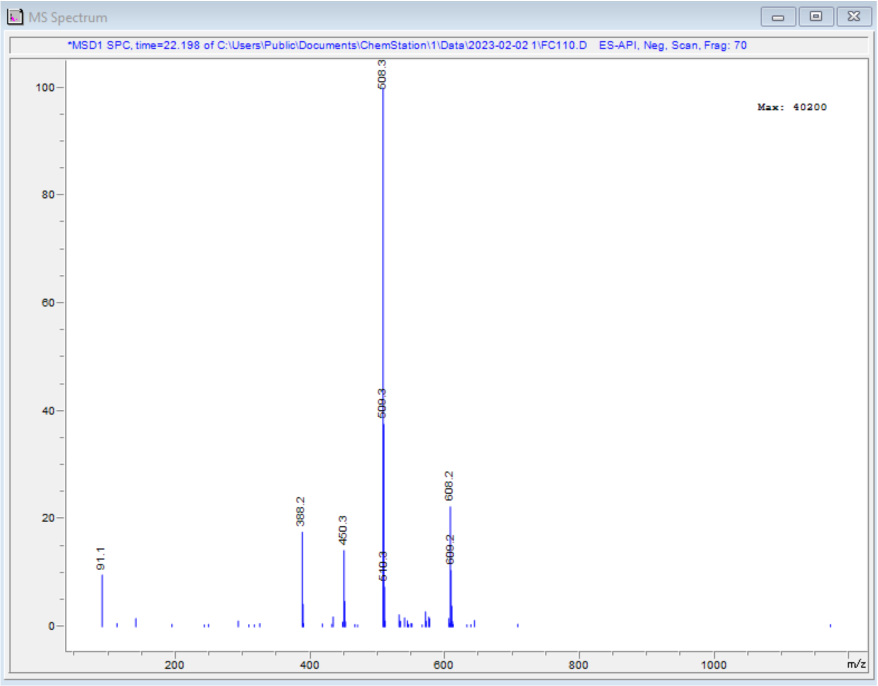

IR spectrum of **Adi-[Tyr(Bn)]$_2$ 4**

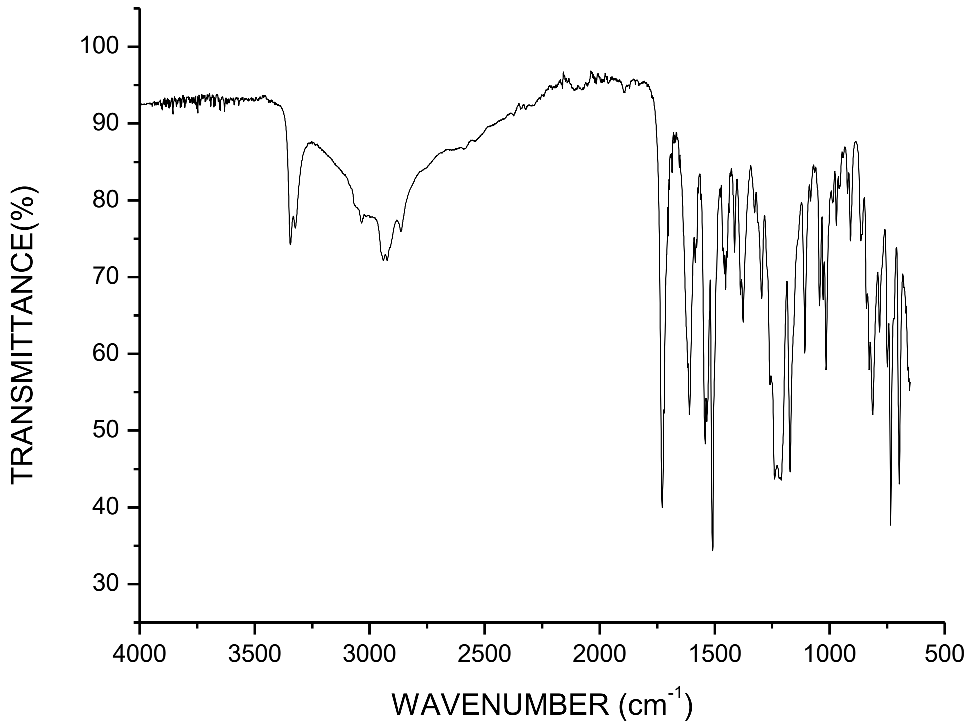

^{1}H NMR spectrum of **Adi-[Tyr(Bn)]$_2$ 4** (DMSO)

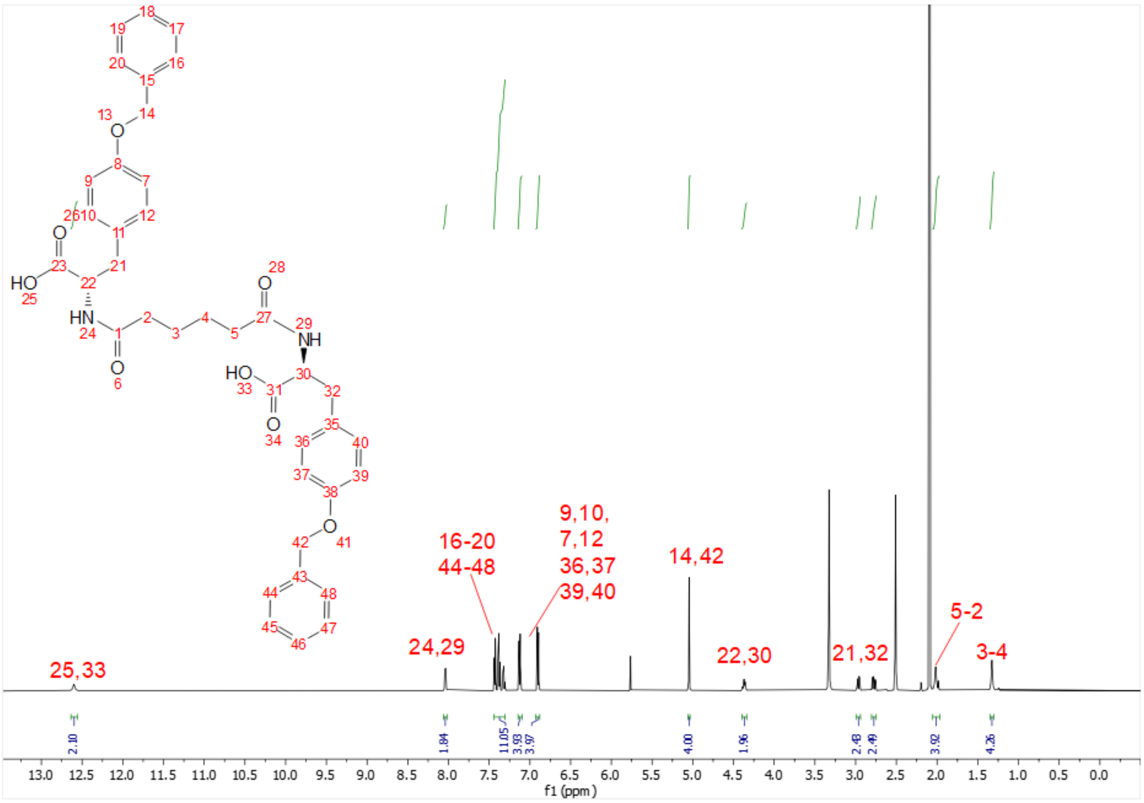

^{13}C NMR spectrum of **Adi-[Tyr(Bn)]$_2$ 4** (DMSO)

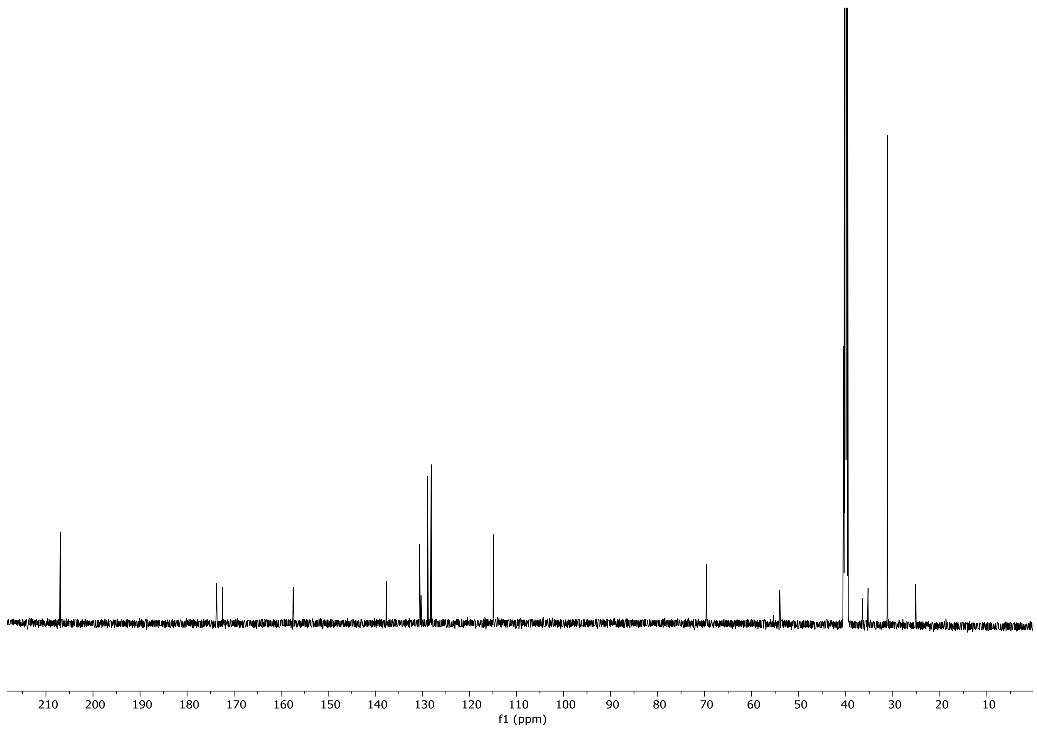

COSY spectrum of **Adi-[Tyr(Bn)]$_2$ 4** (DMSO)

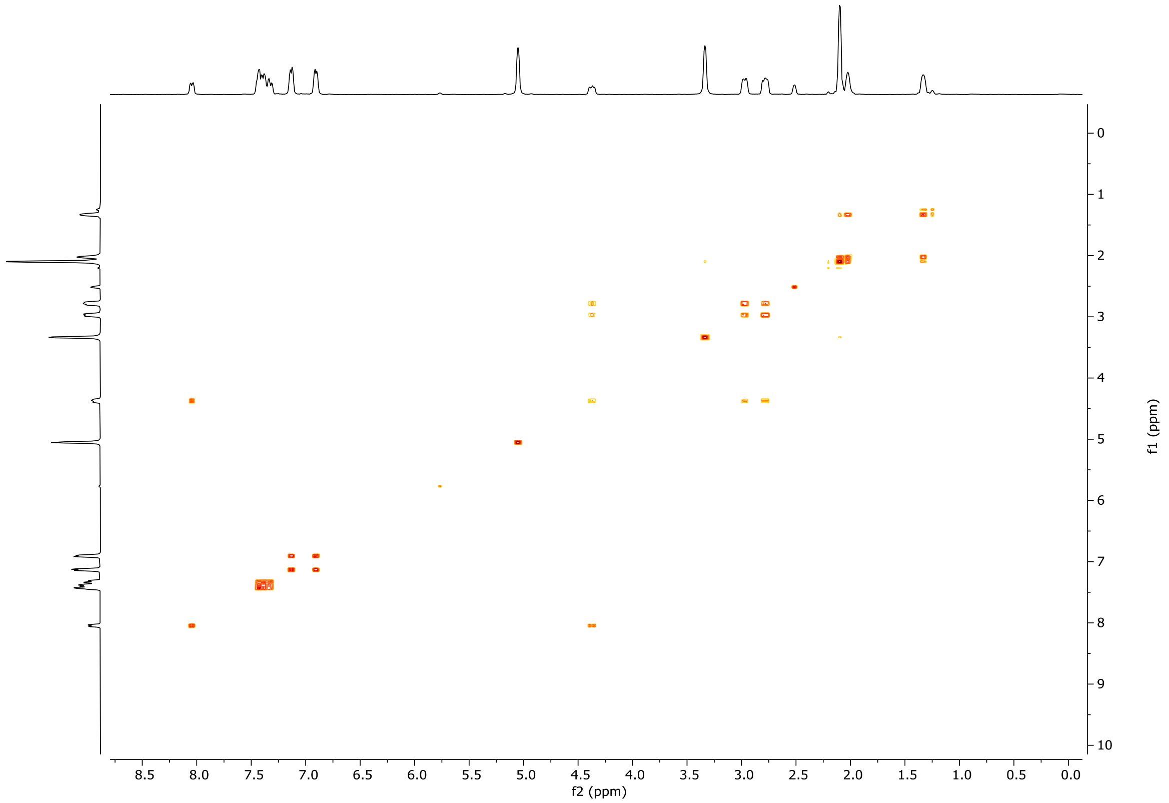

HPLC-MS of **Adi-[Tyr(Bn)]$_2$ 4**

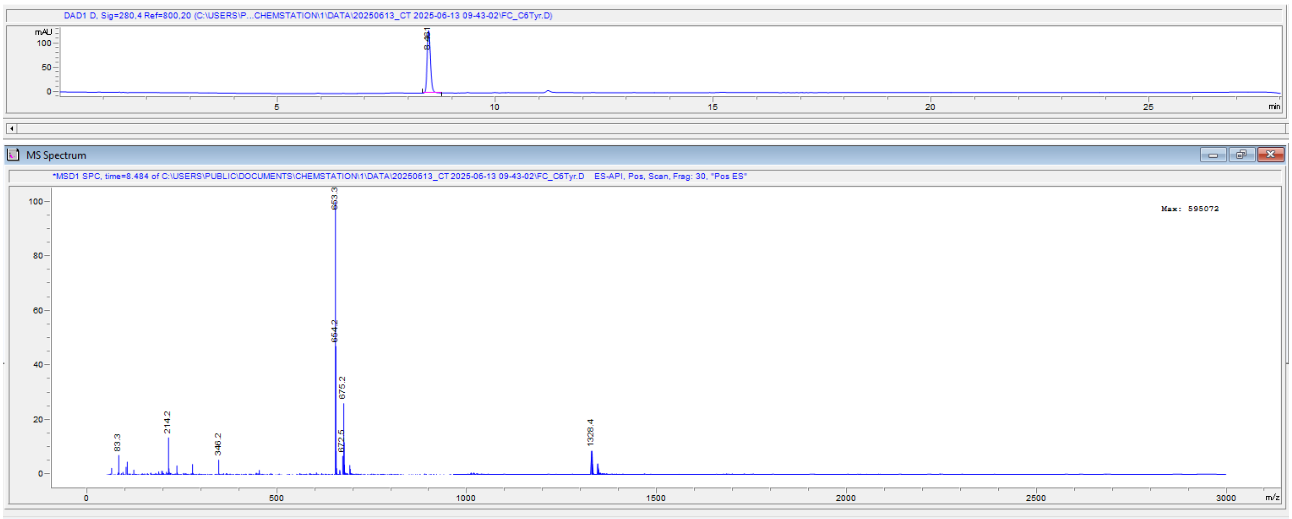

IR spectrum of **Pim-[Tyr(Bn)]$_2$ 5**

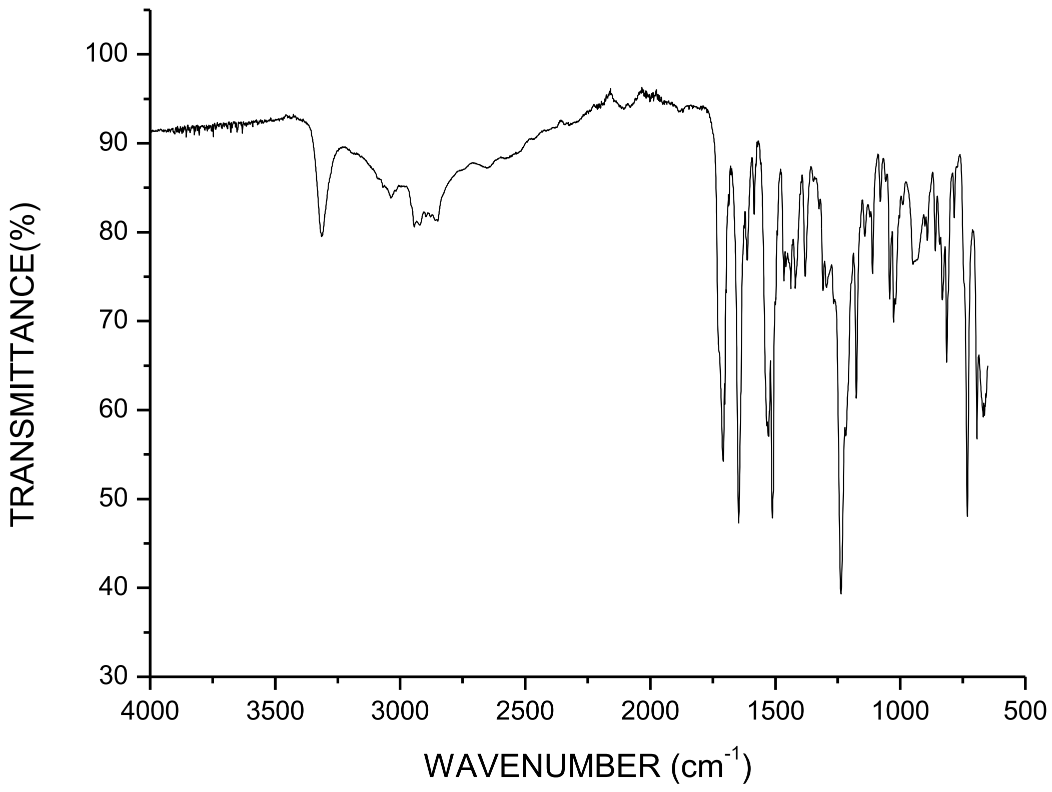

^{1}H NMR spectrum of **Pim-[Tyr(Bn)]$_2$ 5** (DMSO)

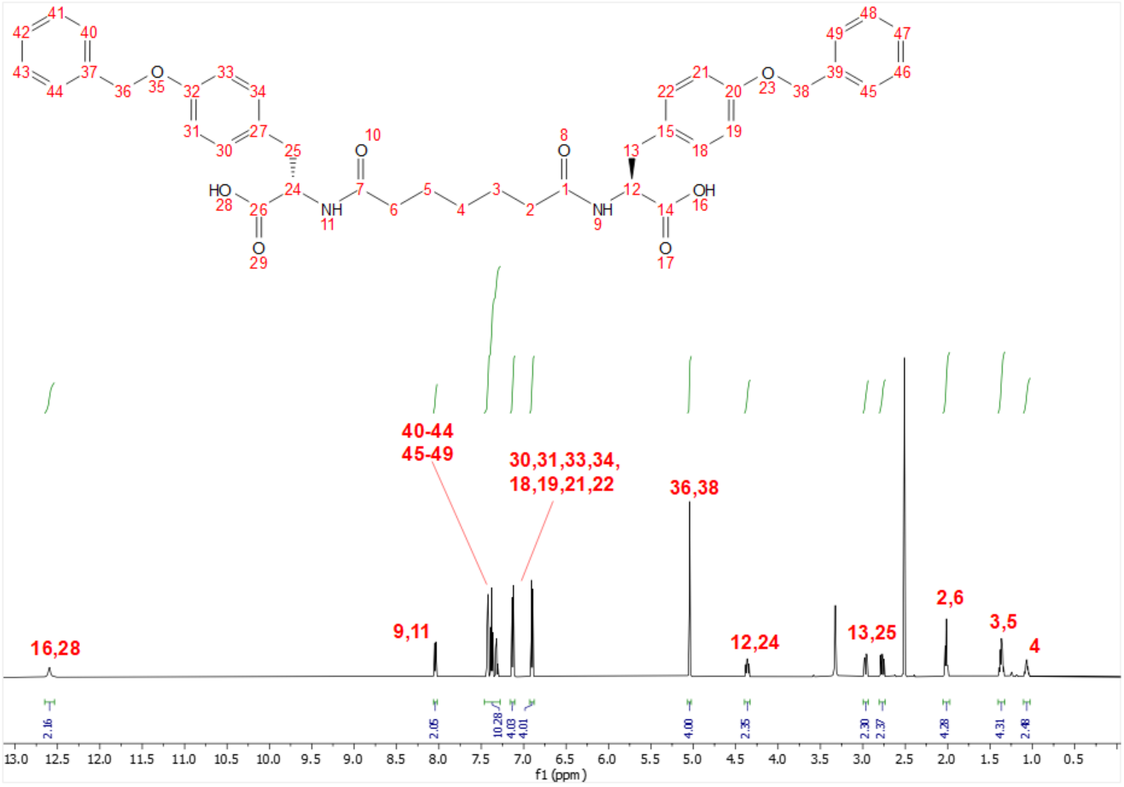

^{13}C NMR spectrum of **Pim-[Tyr(Bn)]$_2$ 5** (DMSO,d_6)

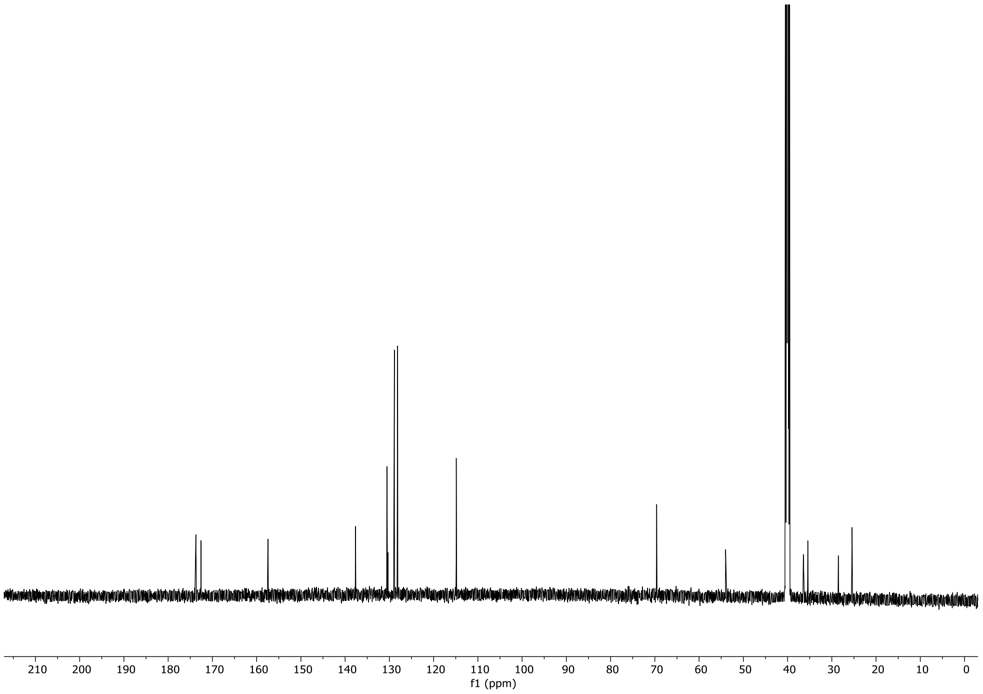

COSY spectrum of **Pim-[Tyr(Bn)]$_2$ 5** (DMSO,d_6)

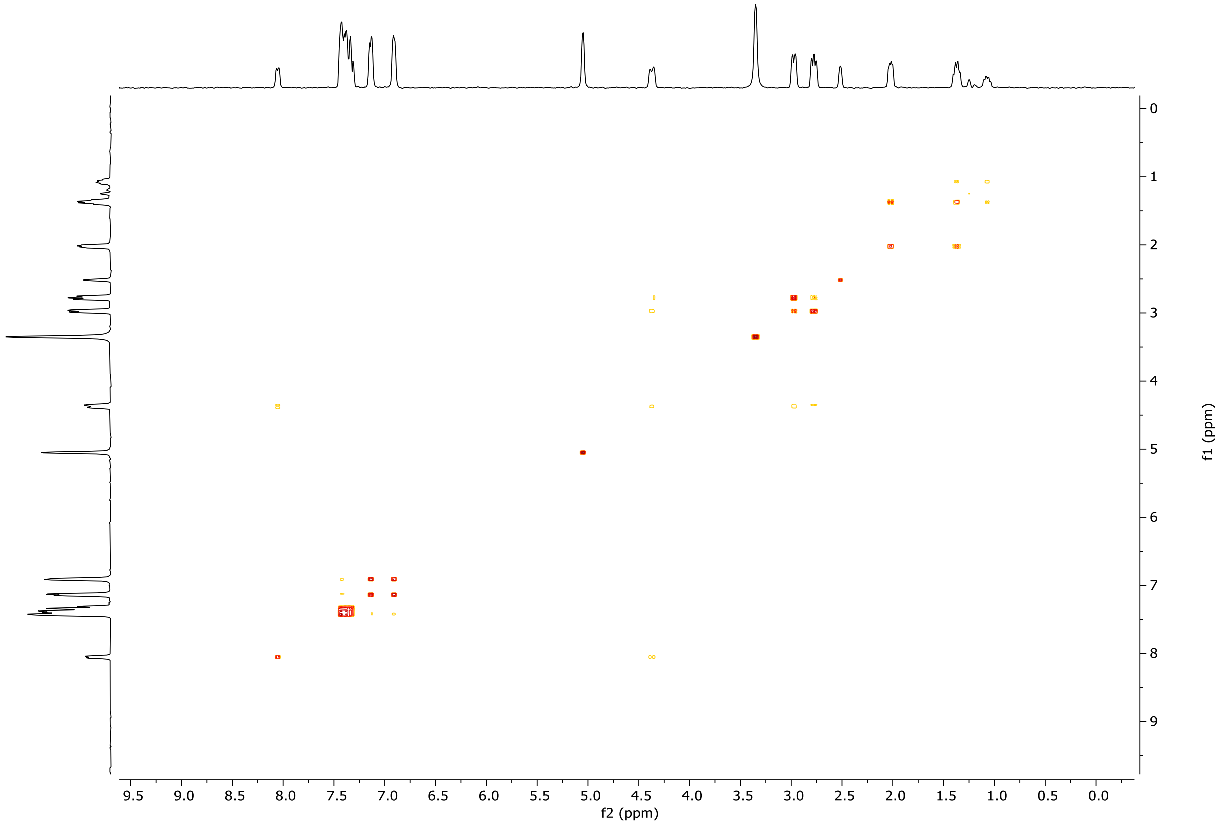

HPLC-MS of **Pim-[Tyr(Bn)]$_2$ 5**

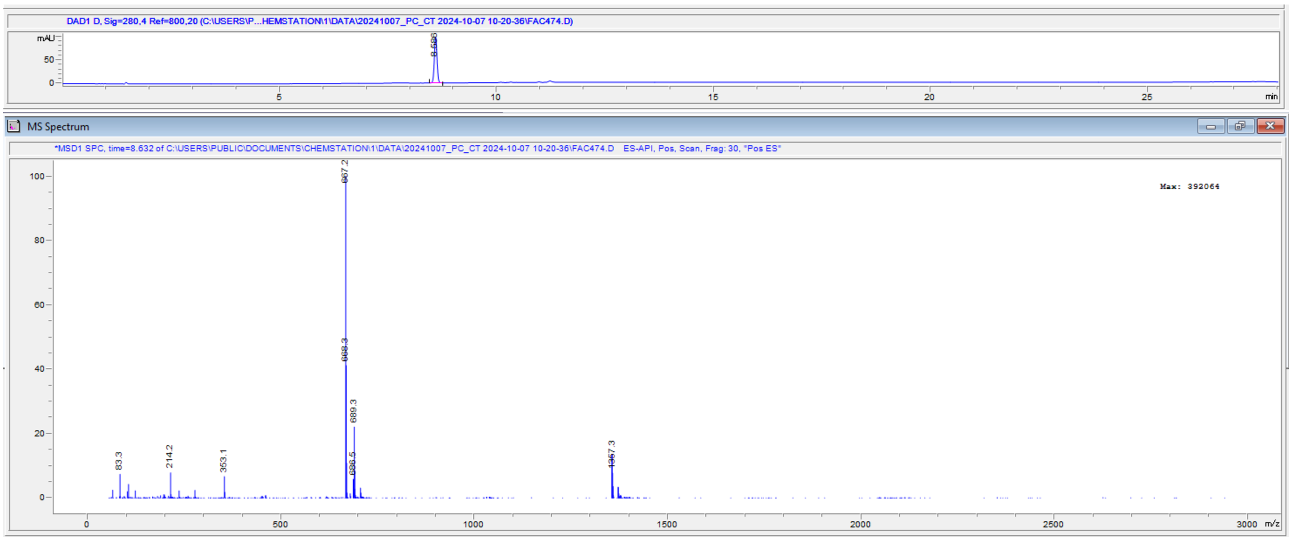

IR spectrum of **Aze-[Tyr(Bn)]$_2$ 6**

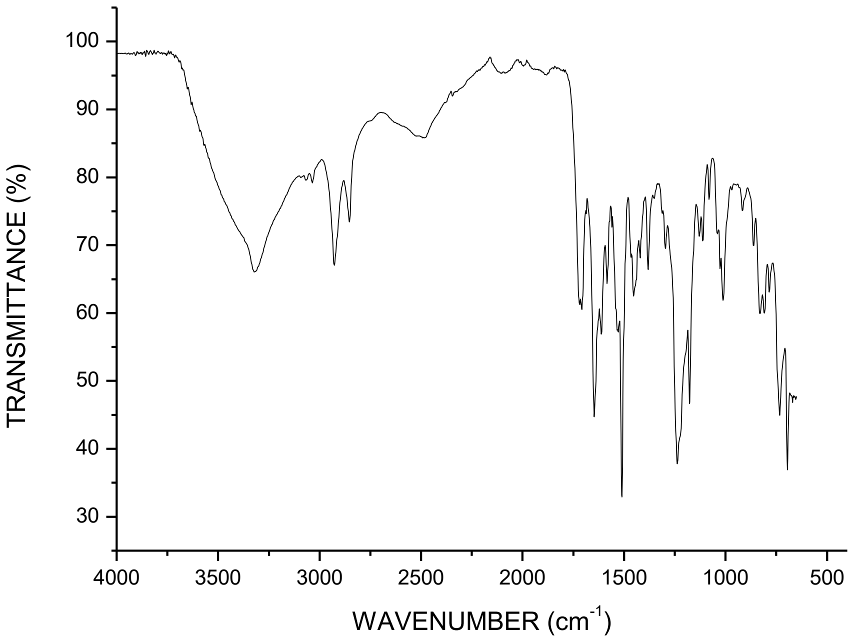

^{1}H-NMR spectrum of **Aze-[Tyr(Bn)]$_2$ 6** (CD_3OD)

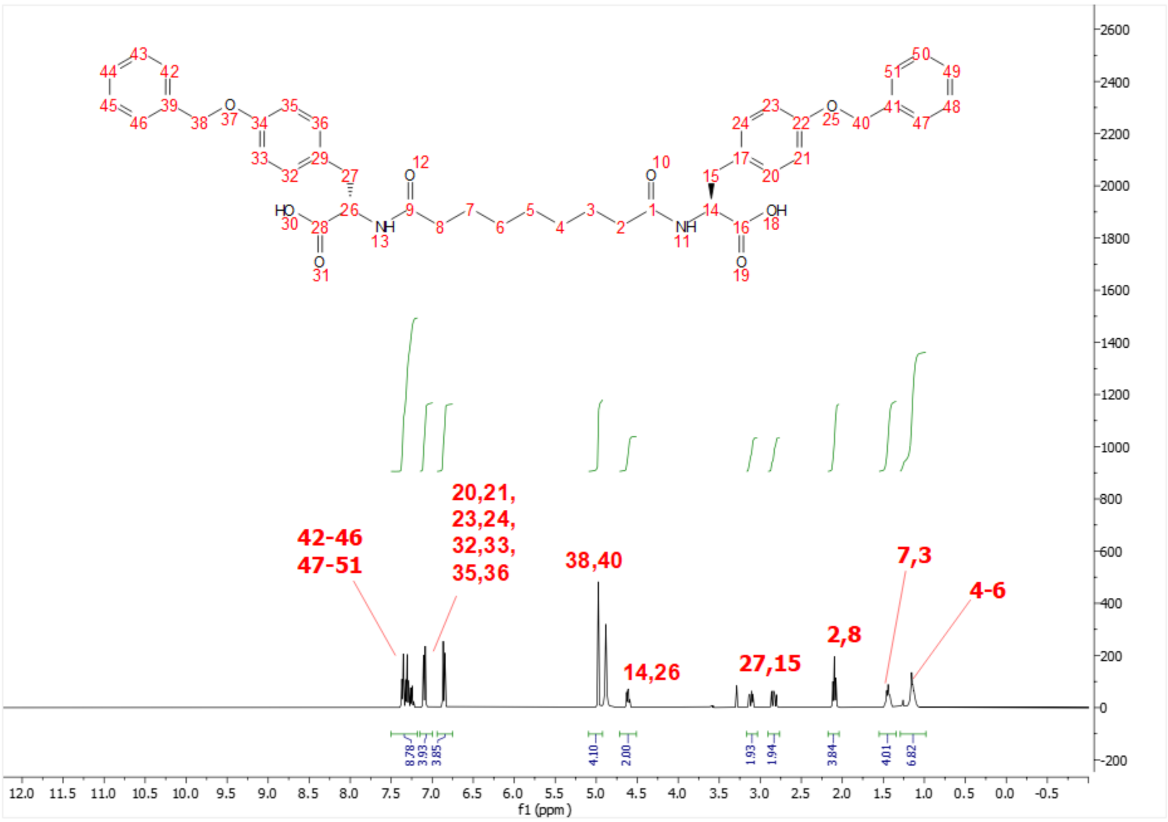

^{13}C-NMR spectrum of **Aze-[Tyr(Bn)]$_2$ 6** (CD_3OD)

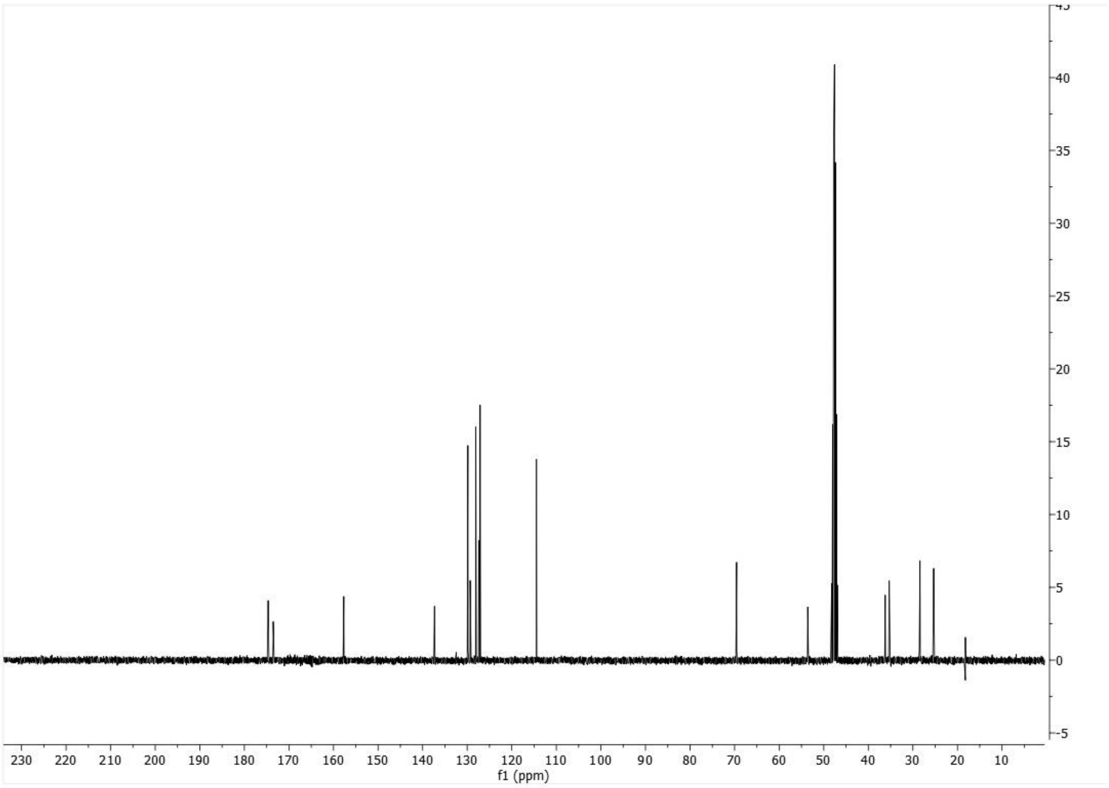

COSY spectrum of **Aze-[Tyr(Bn)]$_2$ 6** (CD_3OD)

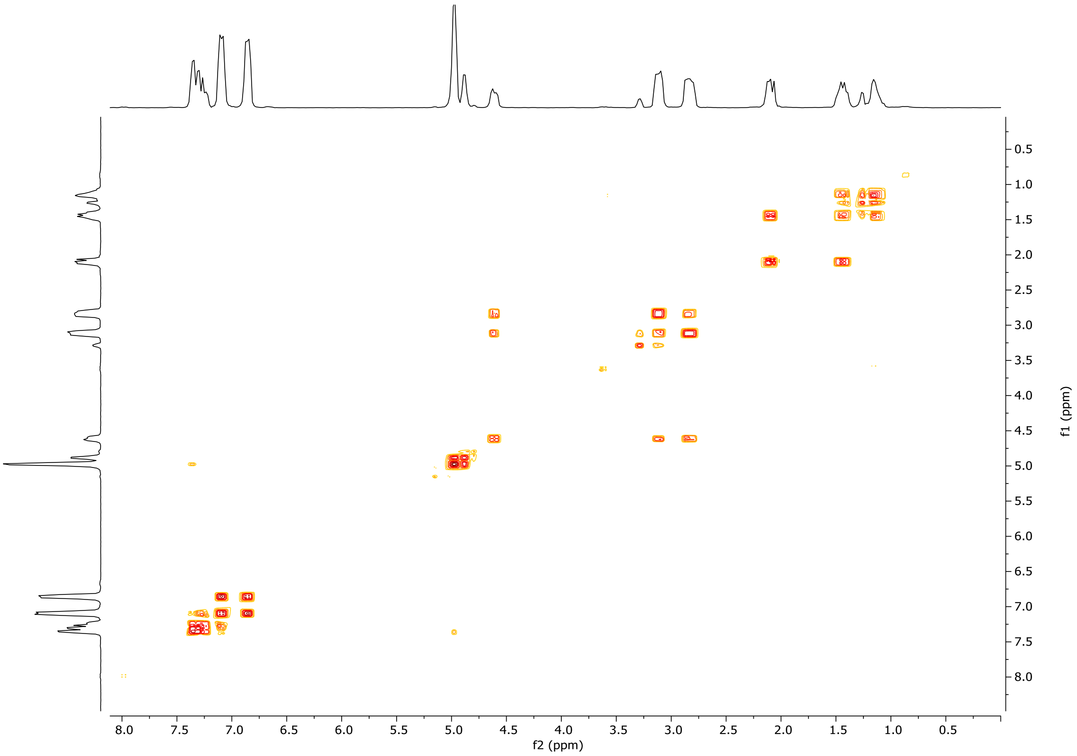

HPLC-MS of **Aze-[Tyr(Bn)]$_2$ 6**

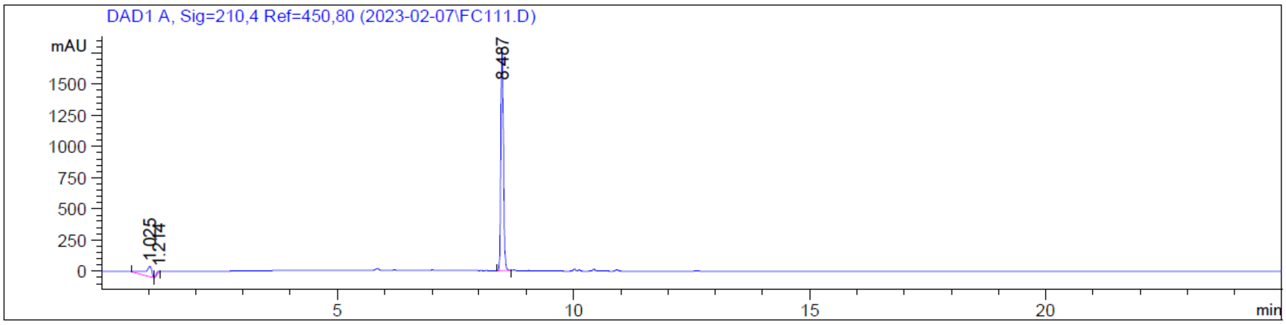

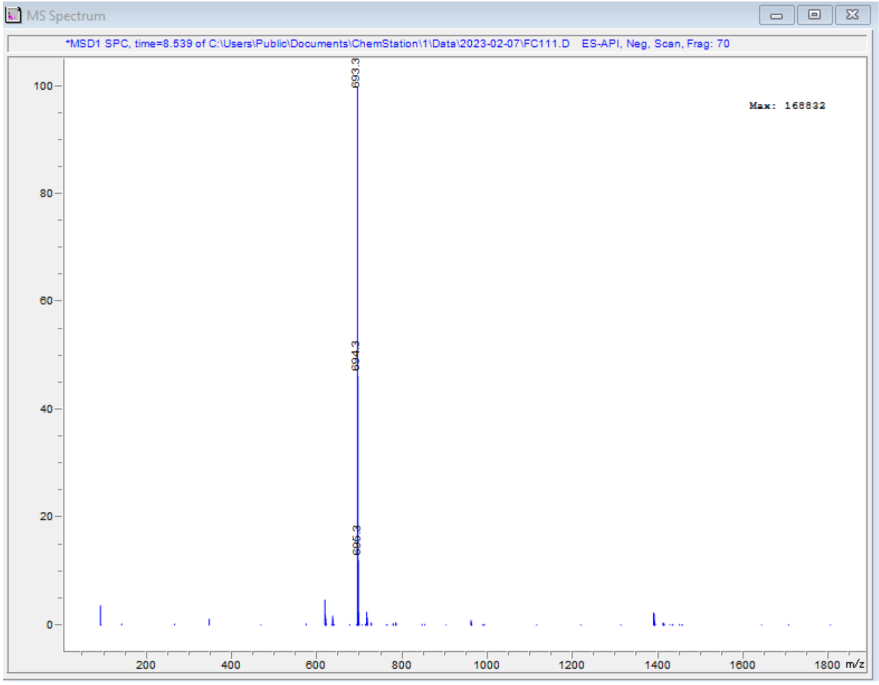

Table S1. Minimum gelation concentration (MGC) values and concentration ranges tested for Cap-Tyr(OBn) **1**, Lau-Tyr(OBn) **2**, Pal-Tyr(OBn) **3**, Adi-[Tyr(OBn)$_2$] **4**, Pim-[Tyr(Bn)]$_2$ **5**, and Aze-[Tyr(Bn)]$_2$ **6**, determined by vial inversion method.

Gelator	MGC (w/V)	Trials (w/V)
Cap-Tyr(OBn) **1**	/	2.0-1.0-0.8%
Lau-Tyr(OBn) **2**	/	2-1-0.5-0.4-0.3-0.2%
Pal-Tyr(OBn) **3**	/	2-1-0.5-0.4-0.3-0.2%
Adi-[Tyr(OBn)$_2$] **4**	0.6%	1-0.8-0.6-0.5%
Pim-[Tyr(Bn)]$_2$ **5**	/	1.0-0.8-0.5%
Aze-[Tyr(Bn)]$_2$ **6**	0.1%	0.5-0.3-0.2-0.1-0.05%

A)

B)

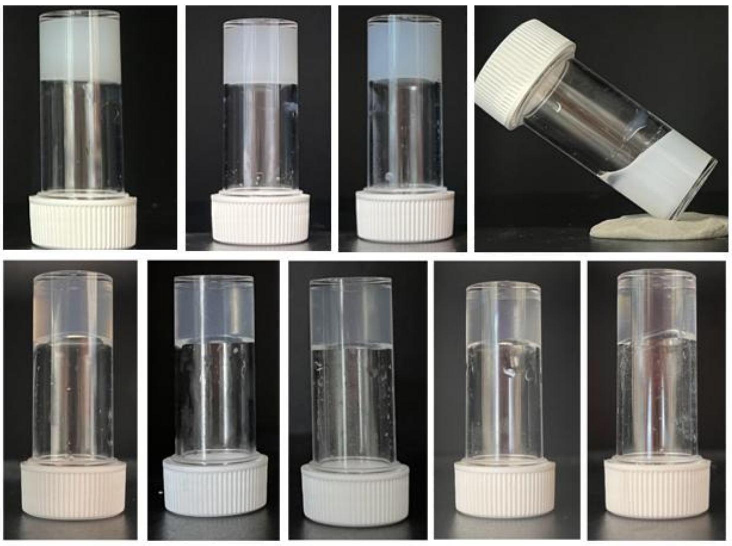

Figure S1. Photographs of the trials for the measurement of the MGC: **A)** Adi-[Tyr(OBn)]$_2$ **4**: 1.0-0.8-0.6-0.5% w/v (MGC 0.6% w/v); **B)** Aze-[Tyr(OBn)]$_2$ **6**: 0.5-0.3-0.2-0.1-0.05% w/v (MGC 0.1% w/v).

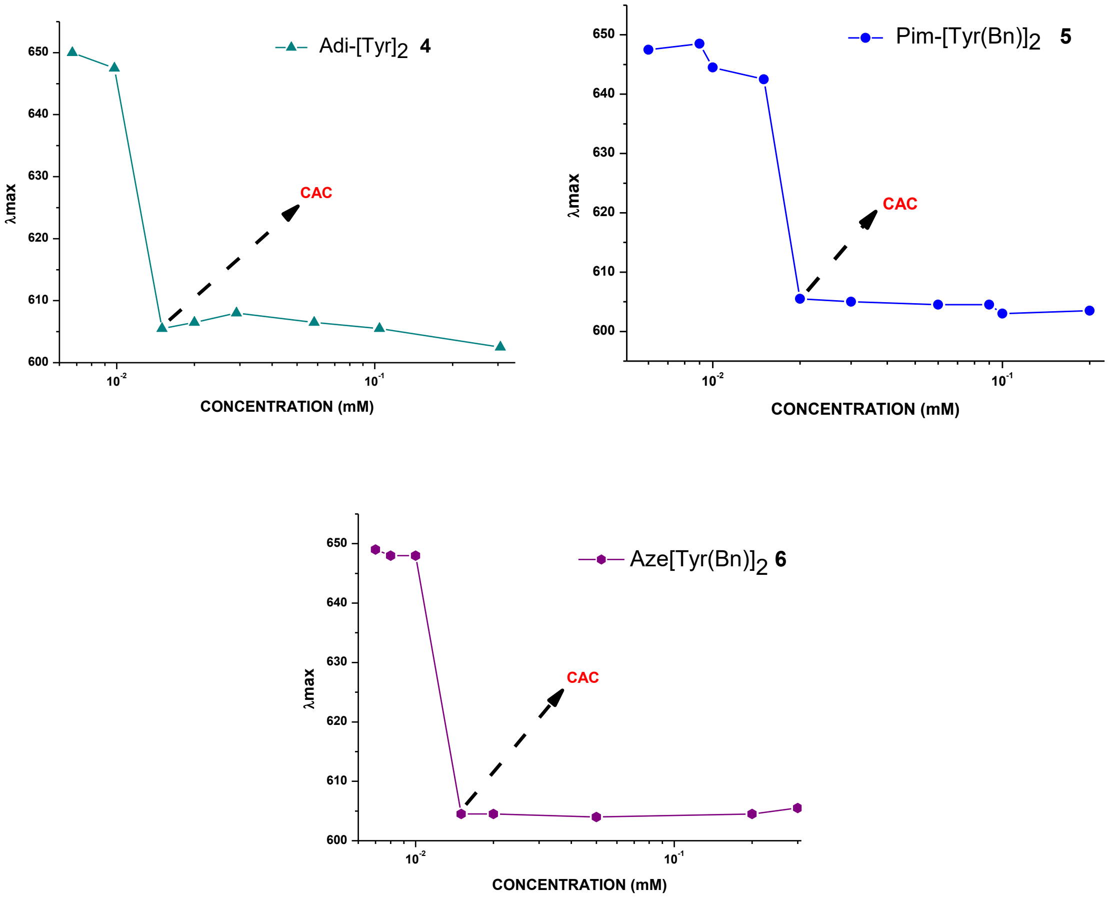

Figure S2. Determination of the CAC with the Nile Red Fluorescence assay for compounds Adi-[Tyr(OBn)]$_2$ **4**, Pim-[Tyr(OBn)]$_2$ **5** and Az-[Tyr(OBn)]$_2$ **6.**

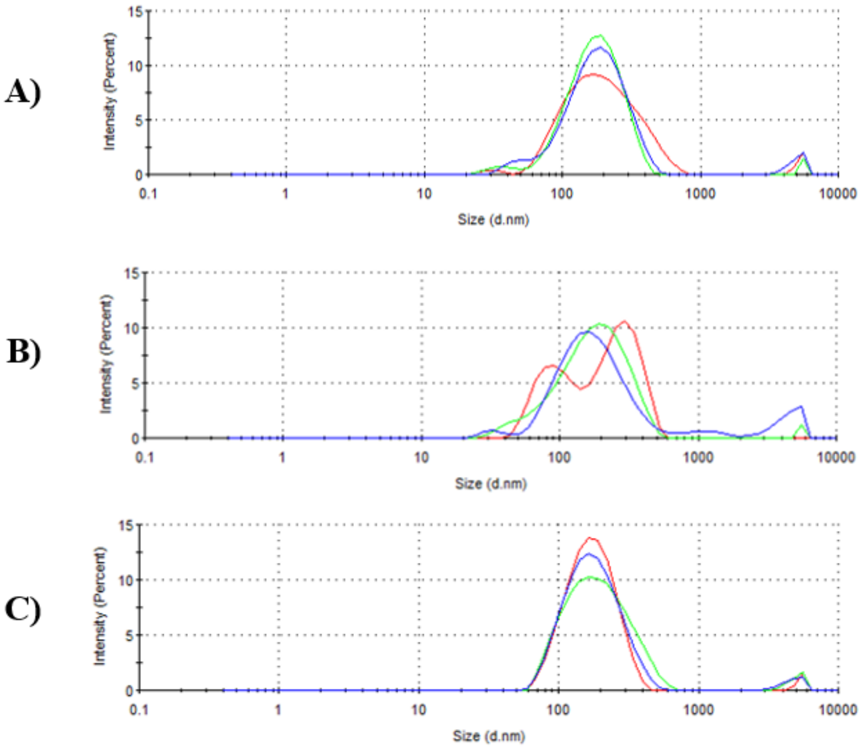

Figure S3. Size distribution by intensity of the particles formed in basic aqueous solutions of gelators: (**A**) Adi-[Tyr(OBn)]$_2$ **4** (0.06 mM); (**B**) Pim-[Tyr(OBn)]$_2$ **5** (0.1mM) and (**C**) Az-[Tyr(OBn)]$_2$ **6** (0.2 mM).

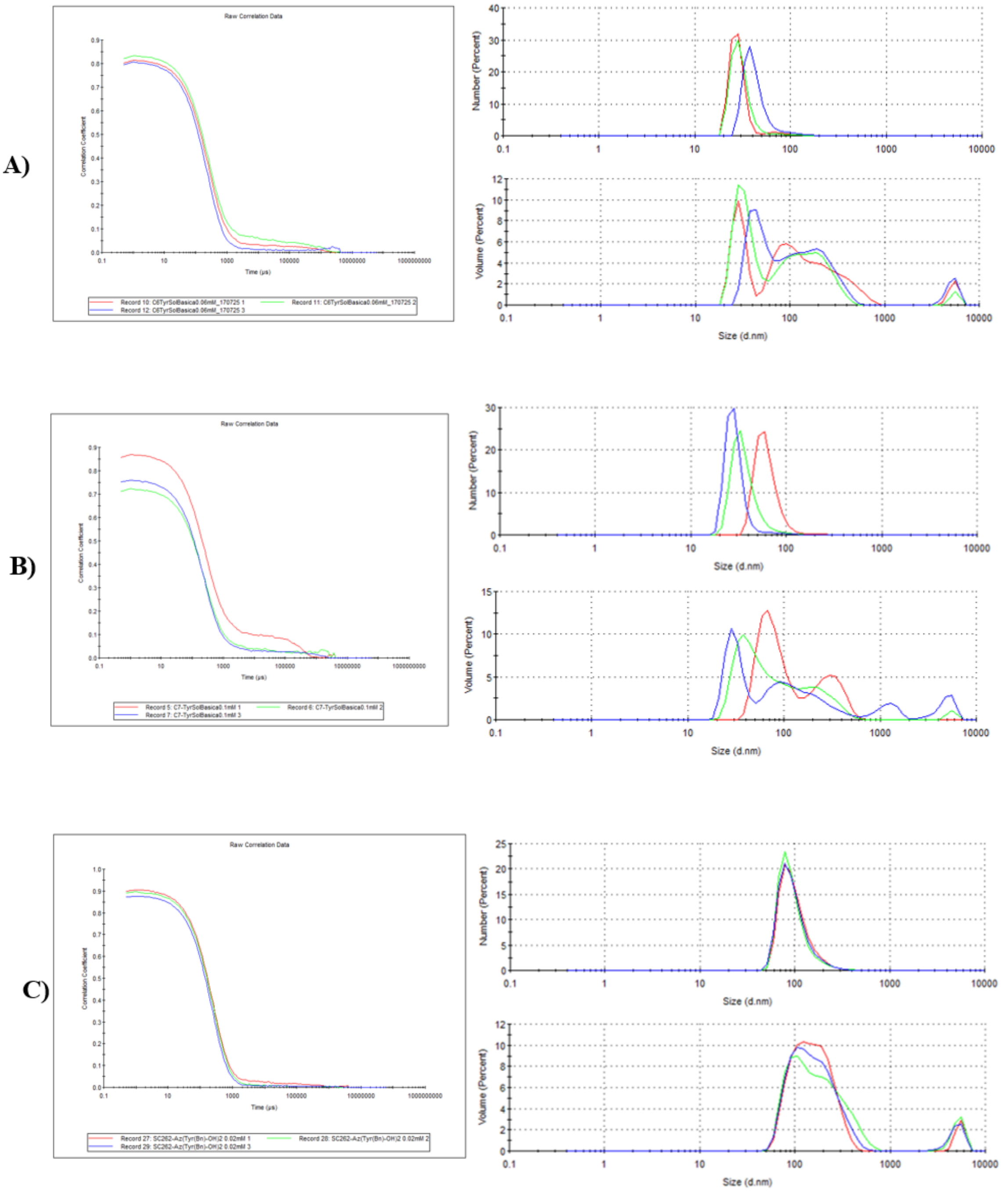

Figure S4. From top to bottom, DLS correlation coefficient, number and volume analysis of particles: (**A**) Adi-[Tyr(OBn)]$_2$ **4** (0.06 mM); (**B**) Pim-[Tyr(OBn)]$_2$ **5** (0.1mM) and (**C**) Az-[Tyr(OBn)]$_2$ **6** (0.2 mM).

Table S2. Crystal data and refinement details for Lau-Tyr(Bn) **2,** Pal-Tyr(Bn) **3**, and Pim-[Tyr(Bn)]$_2$ **5** collected at RT.

	**Lau-Tyr(Bn) 2**	**Pal-Tyr(Bn) 3**	**Pim-[Tyr(Bn)]$_2$ 5**
**Temperature (K)**	293	293	293
**Formula**	$C_{28}H_{39}NO_4$	$C_{32}H_{47}NO_4$	$C_{39}H_{42}N_2O_8$
**FW (g/mol)**	453.60	509.70	666.74
**Crystal System**	monoclinic	monoclinic	monoclinic
**Space Group**	$P2_1$	C2	C2
**a (Å)**	5.810(3)	18.724(3)	29.960(3)
**c (Å)**	9.616(3)	5.0607(5)	5.1023(7)
**c (Å)**	23.863(9)	32.989(5)	24.285(2)
**α (°)**	90	90	90
**β (°)**	93.62(3)	98.190(14)	110.626(10)
**γ (°)**	90	90	90
**Volume (Å^3)**	1330.6(9)	3094.0(7)	3474.4(7)
**Z**	2	4	4
**ρ_{calc} (g/cm^3)**	1.132	1.094	1.275
**μ (mm^{-1})**	0.075	0.071	0.089
**Meas. Ref.**	6671	12602	8283
**Indep. Ref.**	5221	6792	5471
**Largest diff. peak/hole (e/Å^3)**	0.16/-0.17	0.13/-0.15	0.21/-0.20
**R_1**	0.0964	0.0671	0.0804
**wR_2**	0.3456	0.2202	0.2286

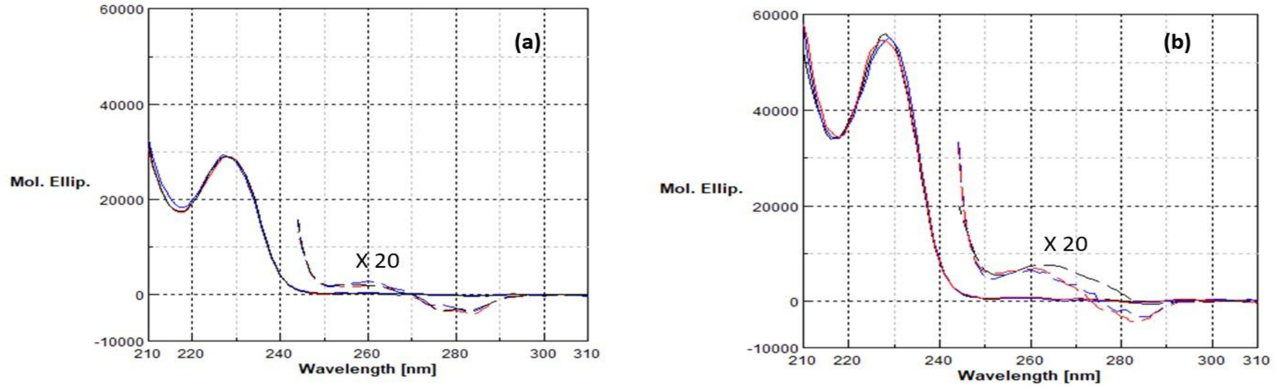

Figure S5. ECD spectra recorded in methanol at 0.5% w/v. Panel (a) shows the spectra of the amphiphilic derivatives **1** (12.6 mM, black traces), **2** (11.0 mM, blue traces), and **3** (9.80 mM, red traces). Panel (b) shows the spectra of the bolaamphiphilic derivatives **4** (7.66 mM, black traces), **5** (7.49 mM, blue traces), and **6** (7.19 mM, red traces). Measurements were performed in a 0.01 cm cell (solid lines) and in a 0.1 cm cell (dashed lines, multiplied by a factor of 20 for clarity). Ellipticity values are expressed as molar ellipticity (deg cm^2 dmol^{-1}).

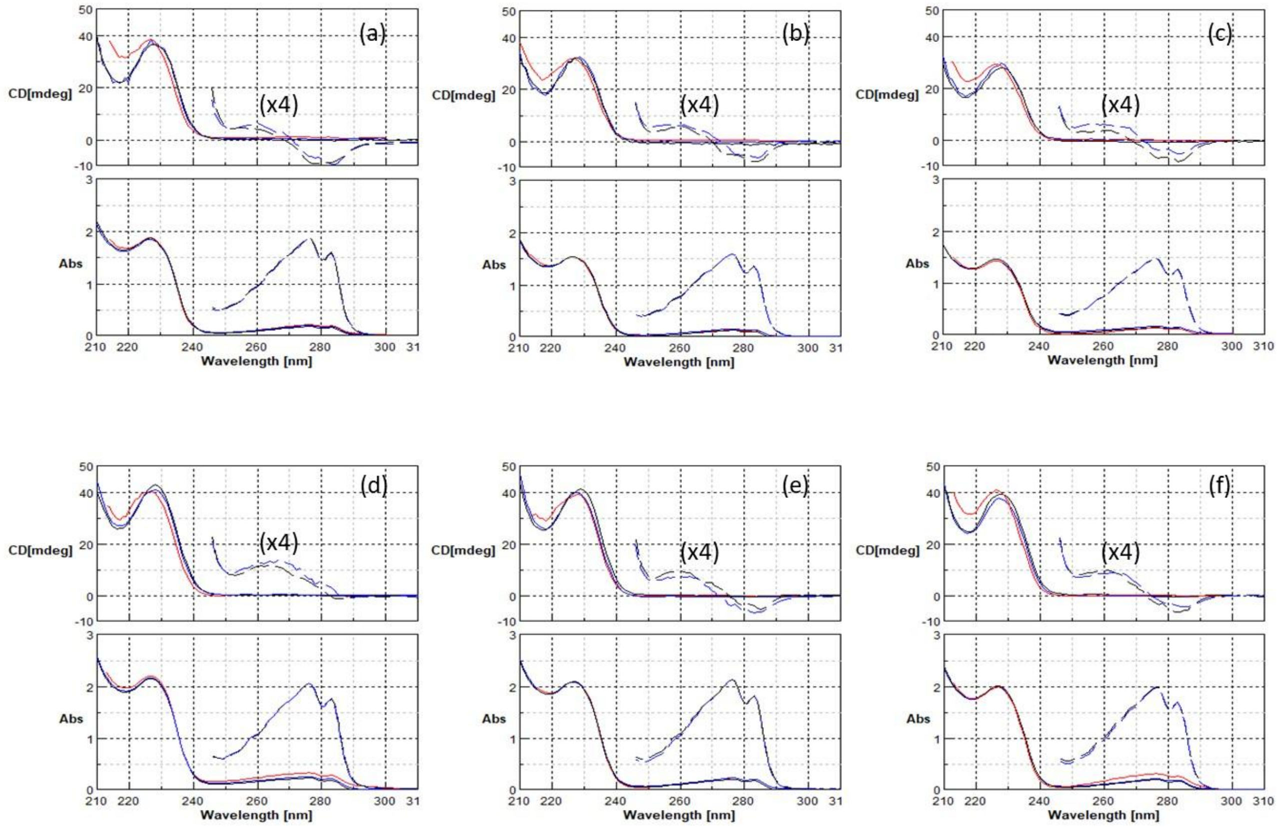

Figure S6. ECD/UV spectra, recorded in methanol on dilution, of amphiphilic derivatives **1** (a), **2** (b), **3** (c), and bolaamphiphilic derivatives **4** (d), **5** (e), and **6** (f). Investigated concentrations were 0.5% w/v depicted with black traces (pathlength (p.l.) 0.01 cm solid lines, p.l. 0.1 cm dashed lines), 0.05% w/v depicted with blue traces (p.l. 0.1 cm solid lines, p.l. 1 cm dashed lines), and 0.005% w/v depicted with red solid lines (p.l. 1 cm). Ellipticities are expressed as millidegrees, and the ECD signals corresponding to the black and blue dashed lines are multiplied by a factor of 4 for clarity.

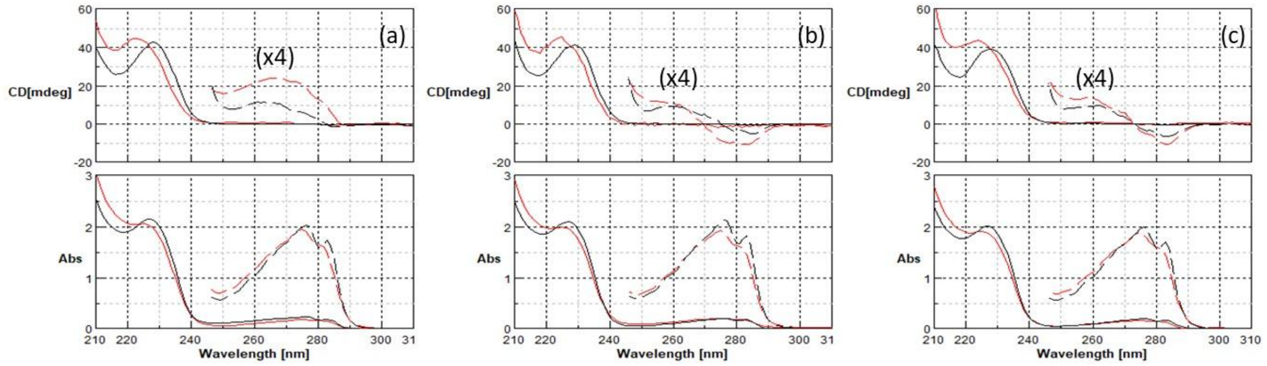

Figure S7. ECD/UV spectra recorded on bolaamphiphilic derivatives **4** (a), **5** (b), and **6** (c) at 0.5% w/v in methanol (black traces) and alkaline water (red traces). Solid lines correspond to measurements in a 0.01 cm cell, while dashed lines correspond to measurements in a 0.1 cm cell and have been multiplied by a factor of 4 for clarity. Ellipticities are expressed as millidegrees.

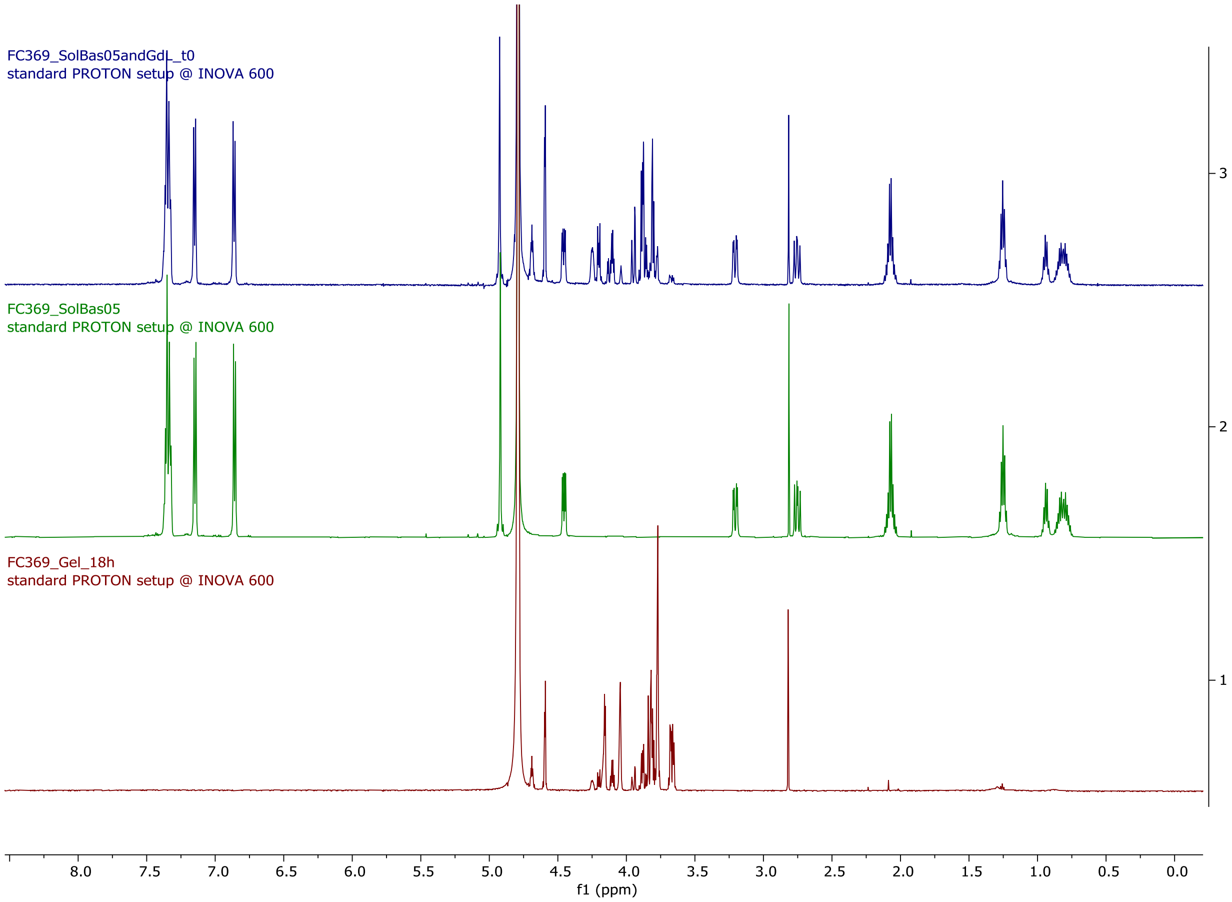

Figure S8. (top) ^{1}H NMR spectrum after the addition of GdL (t = 0), (middle) ^{1}H NMR spectrum of the basic solution before addition of GdL, (bottom) ^{1}H NMR spectrum after the addition of GdL (t = 18 h). Only the signals due to GdL are visible.
